# Vaccination strategies, public health impact and cost-effectiveness of dengue vaccine TAK-003: A modeling case study in Thailand

**DOI:** 10.1371/journal.pmed.1004631

**Published:** 2025-06-17

**Authors:** Jing Shen, Elizaveta Kharitonova, Anna Tytula, Justyna Zawieja, Samuel Aballea, Shibadas Biswal, Mayuri Sharma, Supattra Rungmaitree, Rosarin Sruamsiri, Derek Wallace, Riona Hanley

**Affiliations:** 1 Takeda Pharmaceuticals International AG, Zürich, Switzerland; 2 External Contractor, Putnam, Paris, France; 3 Putnam, Krakow, Poland; 4 Putnam, Paris, France; 5 Takeda Vaccines, Inc., Cambridge, Massachusetts, United States of America; 6 Department of Pediatrics, Faculty of Medicine Siriraj Hospital, Mahidol University, Bangkok, Thailand; 7 Takeda Thailand Ltd, Bangkok, Thailand; Universitair Medisch Centrum Utrecht, NETHERLANDS, KINGDOM OF THE

## Abstract

**Background:**

Dengue is an increasing global problem associated with negative health and economic impacts. Vaccination is an important measure to reduce the significant public health and economic burden caused by dengue. Our study assesses the public health impact and cost-effectiveness of a new dengue vaccine, TAK-003, using Thailand as a case study.

**Methods and findings:**

We developed a dynamic transmission model with both host and vector populations, 4 serotype-specific infections, seasonality, and other key elements of dengue natural history. We estimated efficacy of TAK-003 from the DEN-301 trial. We first used the model to determine the optimal cohort age for different vaccination strategies with TAK-003, based on Thai dengue epidemiology. Secondly, we assessed the public health impact of a pragmatic strategy integrating TAK-003 into an existing national immunization program in Thailand. Cost-effectiveness was evaluated from a societal perspective using disability-adjusted life-years (DALYs) over a 20-year horizon.

TAK-003 is estimated to prevent 41%−57% of symptomatic cases and 47%−70% of hospitalizations, with the greatest impact observed when routinely vaccinating children aged 6 years with 10 additional catch-up cohorts. This strategy resulted in 104,415 fewer DALYs and savings of US$1,786 million. If introduced into the national immunization program at 11 years of age (alongside the existing human papillomavirus vaccine), TAK-003 is estimated to prevent 44% of symptomatic cases and 53% of hospitalizations. This strategy prevented 87,715 DALYs and saved US$1,346 million. Sensitivity analyses demonstrated that the results were robust. The main limitations were inherent to the assumptions and simplifications made in the model, which are unavoidable when approximating the impact of vaccination in the real world.

**Conclusions:**

TAK-003 can considerably reduce dengue burden and lead to cost savings in Thailand. These benefits can be maximized by identifying optimal age cohorts for vaccination and adding catch-up programs. Our model can be used to assess the vaccination impact in other dengue-endemic countries.

## Author summary

**Why was this study done?**

- The World Health Organization has identified dengue as a significant global threat.
- There is no adequate treatment for dengue and prevention is key; TAK-003 is the only licensed vaccine that can be used for individuals both with and without a previous dengue infection.
- Our work uses Thailand as an example to demonstrate optimal design of an efficient dengue vaccination program using mathematical modeling.

**What did the researchers do and find?**

- We developed a dynamic transmission model of dengue for TAK-003 to determine the age groups for a vaccination program that would have the maximum impact on reducing symptomatic cases and hospitalizations, based on the patterns of disease in a country, and estimate associated public health impact and cost-effectiveness of the TAK-003 vaccination program.
- Across a range of vaccination strategies explored, it is estimated that TAK-003 could prevent 41%–57% of disease cases and 47%–70% of hospitalizations over 20 years; among the explored strategies, routine vaccination of children aged 6 years with 10 additional catch-up cohorts was found to be most cost-effective and would save US$1,786 million.
- Considering a pragmatic introduction of TAK-003 into the Thai immunization program by co-administration with the existing human papillomavirus vaccination program at age 11, it is estimated that 44% of cases and 53% of hospitalizations could be prevented, and US$1,346 million could be saved over 20 years.

**What do these findings mean?**

- To our knowledge, this is the first study to evaluate the impact of TAK-003 in a national immunization program.
- Our findings demonstrate that the introduction of TAK-003 into Thailand’s national immunization program could reduce the burden of dengue, whilst also delivering cost savings to the government across the healthcare system and beyond.
- Each country should design vaccination programs that are adapted to their pattern of dengue disease, which may mean different target vaccination ages; should a country wish to include catch-up cohorts to accelerate the speed of dengue reduction, this should be modeled during vaccination program design, as optimal target age may differ from that of a program without catch-up.
- A limitation of our study is that not all dengue-related costs could be incorporated into the model, the estimate on the cost effectiveness of TAK-003 is therefore likely to be conservative.

## Abstract 3

In a case study of Thailand, modeling vaccination with the TAK-003 dengue vaccine can help optimize strategies to reduce health and economic burdens of dengue disease.

## Introduction

Dengue is the world’s fastest-spreading vector-borne viral disease [1] and has been identified by the World Health Organization (WHO) as one of the top 10 threats to human life [2]. The worldwide incidence of dengue has increased significantly in the past 50 years. Before 1970, only 9 countries had experienced severe dengue epidemics; however, the disease is now endemic in >125 countries [3,4]. Its spread is likely to continue with growing urbanization, shifting migration patterns, and climate change [5,6]. According to WHO’s global dengue surveillance system, in 2024 there were approximately 14.5 million reported cases (almost 52,000 of them severe) and just under 11,000 dengue-related reported deaths worldwide [7]. Furthermore, in 2024 Thailand reported approximately 103,000 cases including 84 deaths, representing a decrease compared to 2023’s figures (approximately 156,000 cases and 175 deaths) [8]; however, these data may be underestimated [9]. Additionally, dengue is the leading cause of febrile illness in travelers [10,11]. Dengue poses a significant burden on healthcare systems and has necessitated the introduction of field hospitals in underserved regions [12].

Dengue is primarily transmitted via the mosquitoes *Aedes aegypti* and *Aedes albopictus* and is caused by 4 distinct viral serotypes (DENV-1, DENV-2, DENV-3, and DENV-4) [13,14]. Infection with 1 dengue virus (DENV) serotype is associated with long-term protection against the same serotype and a period of temporary protection against infection with other serotypes, in a process known as heterotypic cross-protection [15]. Because infection does not induce long-lived immunity against the other serotypes, a person can be serially infected with all 4 serotypes. The risk of severe dengue is highest in those who have had a previous dengue infection and subsequently become infected with a different serotype (heterotypic reinfection) [16].

Dengue clinical presentations range in symptoms, from a mild flu-like illness to severe, debilitating disease that lasts for several days to weeks. Some individuals also develop persistent post dengue syndrome [17,18]. Approximately 25% of dengue infections lead to clinically apparent disease that manifests as an acute febrile illness [18], and of these, about 5% progress to severe disease [19]. In the most severe form, the infection manifests as dengue hemorrhagic fever or dengue shock syndrome, where plasma leakage and thrombocytopenia occur, leading to hypovolemic shock, major bleeding, organ failure, and possible death [4,20].

Dengue has earned the colloquial description “break-bone fever” due to the high temperatures and severe limb pain experienced by some people in the acute phase [18]. It has the potential to have a substantial impact on an individual’s quality of life at all stages of infection [21–23]. Persistent dengue can last for up to 2 years, with chronic symptoms, including fatigue and depression [24–27].

There is no proven effective antiviral treatment for dengue, with individuals with milder forms of dengue requiring symptomatic care (e.g., fluid intake and analgesia), and patients with severe dengue requiring hospitalization [28]. Instead, the emphasis has been on prevention. One form of prevention is vector control, but there is a lack of evidence on the effectiveness of conventional vector control methods, especially in the long term [29]. More recently, there has been some success with the release of vectors infected with *Wolbachia* [30], and further research is required on the scalability, deployment, long-term sustainability, and overall effectiveness of this intervention [31]. The other tenet of prevention is vaccination. Use of the first licensed tetravalent vaccine CYD-TDV (Dengvaxia) is limited owing to it increasing the risk of severe infection in dengue-naive individuals and consequently requiring prior serotesting [32]. This is due to the phenomenon of ADE (Antibody-Dependent Enhancement) associated with dengue infections. ADE is also the reason that in dengue vaccine development, dengue seronegative individuals are a population that requires special consideration [33].

A new vaccine, TAK-003 (developed by Takeda under the proprietary name Qdenga), has been designed to prevent all dengue serotypes, regardless of previous exposure. TAK-003 is a live-attenuated dengue vaccine that incorporates the DENV-2 backbone and activates a broad, dengue-specific immune response directed against multiple components of the vaccine virus. It works through both humoral and cell-mediated immunity [34–38].

The efficacy and safety of TAK-003 have been demonstrated in a large phase 3 randomized controlled trial (DEN-301), also known as TIDES (ClinicalTrials.gov: NCT02747927) [39]. This study enrolled healthy children and adolescents (aged 4–16 years, *n* = 20,099) living in dengue-endemic areas of Latin America and Asia. The study was adherent to WHO dengue clinical guidelines on study design, population inclusion, duration, and powering of endpoints [40], and captured baseline serostatus for all participants with a 54-month follow-up, in line with the duration of 3–5 years recommended by WHO for endemic trial sites.

TAK-003 was administered as 2 doses of subcutaneous injection, approximately 3 months apart [41]. The efficacy against virologically confirmed dengue (VCD) estimated as the primary endpoint of the trial at 12 months post second dose was 80.2% (95% confidence interval 73.3%–85.3%), and efficacy was comparable in seronegative and seropositive participants. In addition to this, there was 90.4% efficacy against dengue leading to hospitalization at 18 months post second dose (95% confidence interval 82.6%–94.7%). The last follow-up study reported that this efficacy was maintained, with the cumulative efficacy from the first dose to 4.5 years after the second dose being 61.2% against VCD, and correspondingly 84.1% against hospitalized VCD [42]. No evidence of elevated risk of hospitalization or severe disease among seronegative individuals was identified. TAK-003 data have been reviewed extensively by a number of regulatory bodies, including the European Medicines Agency, which approved it for use in individuals aged ≥4 years [43]. Approval has also been granted in Argentina, Brazil, Colombia, Great Britain, Indonesia, and Thailand for the prevention of dengue in individuals, irrespective of their baseline serostatus, recognizing the positive risk-benefit profile and significant burden on both seropositive and seronegative individuals. Recently, TAK-003 has been recommended by WHO for programmatic use in children aged 6–16 years in settings with high dengue transmission intensity [44].

In Thailand, dengue is endemic throughout the country, with large epidemics occurring every few years. The incidence of dengue in Thailand has reflected the global trend, with >24,030 cases and >20 deaths reported in 2023 since January 1 (as of June 21, 2023), an infection rate of 4.2 times higher than in 2022 [45]. Additionally, actual cases of dengue are likely to be higher than reported [46]. The acute presentation of dengue has also been reported as being a serious burden to the Thai healthcare system. There are also broader economic aspects of dengue to consider in Thailand, such as the potential impact on the tourism industry [47].

For the introduction of a vaccine to healthcare systems, it is necessary to determine the age of routine vaccine administration. Dengue vaccination strategies should be based on country-specific dengue epidemiology [48]. This is because dengue is a complex disease that involves the interplay of host, vectors, immunological protection, and many environmental factors, such as seasonality [49]. Dengue affects a broad range of ages, and the distribution of dengue cases across ages and the incidence profile can vary significantly from country to country [20,50]. Therefore, it is important to identify the optimal age cohort for vaccination strategies for the country in question to achieve the most efficient use of the vaccine.

The objectives of the current study were therefore 2-fold. First, we aimed to determine the optimal age cohorts for TAK-003 vaccination in Thailand based on Thai-specific dengue epidemiology. Strategies involving both routine vaccination only and routine vaccination combined with catch-up programs (one-off vaccinations of older cohorts) were explored to assess the public health impact and cost-effectiveness. Second, we explored the benefits of a pragmatic introduction of TAK-003 at age 11 years, co-administered with the existing human papillomavirus (HPV) vaccination, to the Thai national immunization program (NIP).

## Methods

### Transmission model

**Fig 1:**
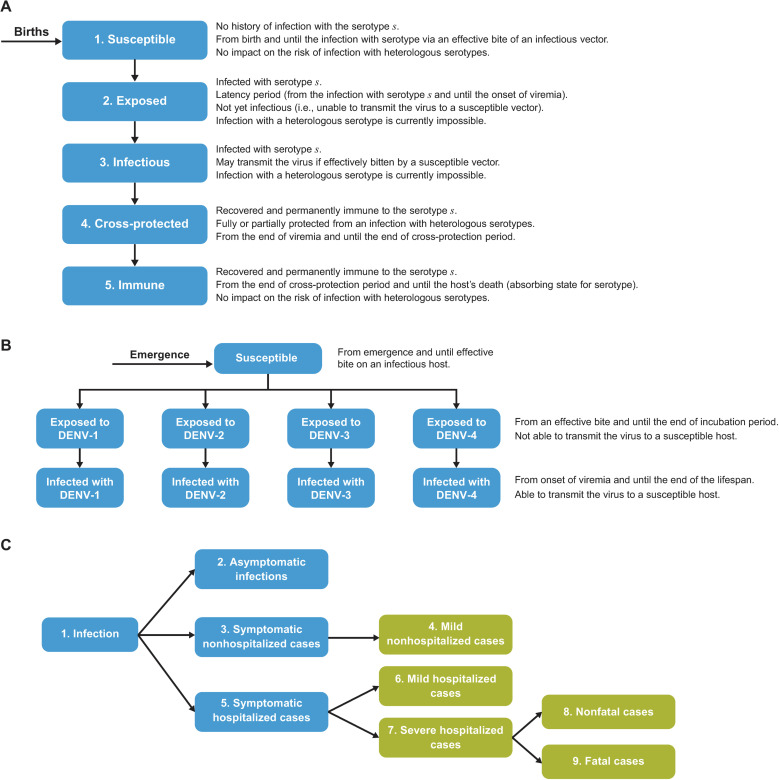
(A) Infection process in hosts. (B) Infection process in vectors. (C) Infection outcomes in host. **(A)** Infection process in hosts (human), for a single serotype. The model considers all possible combinations of statuses for each of the 4 serotypes, resulting in 625 compartments per age cohort (without vaccination). It keeps the memory of serotypes already acquired, but not the order of their acquisition. The transition from the state “Susceptible” to the state “Exposed” (period of incubation) occurs upon an effective bite of a vector carrying the serotype in question. In the state “Infectious” (period of viremia), the hosts may transmit the serotype they are infected with if a susceptible vector feeds upon them. Co-infection is assumed impossible. Following the period of viremia, the hosts are assumed to be temporarily fully protected from a heterologous infection (state “Cross-protected”) and permanently protected from a homologous infection (state “Immune”). **(B)** Infection process in vectors (mosquito). For vectors, the model includes 9 compartments (i.e., susceptible, exposed to a specific serotype, infectious of a specific serotype). Due to short life expectancy, the vectors were assumed to be able to carry only 1 serotype in their lifetime. **(C)** Infection outcomes. Hosts infected with dengue may be asymptomatic or symptomatic. All severe and a proportion of mild symptomatic infections result in hospitalization. Severe infections may result in dengue-caused death. A proportion of patients with symptomatic dengue may also develop persistent dengue (not displayed in the diagram). Each infection outcome is associated with specific costs and impact on health-related quality of life. DENV, dengue virus.

A dynamic transmission model was developed to model the impact of vaccination with TAK-003. The modeling of dengue natural history was informed by the work of Coudeville and colleagues in 2012 [51], and was based on a deterministic compartmental model with both host and vector populations, seasonality, and 4 serotype-specific infections followed by permanent homologous and temporary heterologous protection, as well as the increased probability of clinical disease and hospitalization with second dengue infections. Model inputs for efficacy of TAK-003 were derived from the DEN-301 trial (ClinicalTrials.gov: NCT02747927) [42]. A schematic of the model is provided in Fig 1 and a detailed description of the model structure is provided in S1 File.

The direct effects of TAK-003 were modeled to reflect the current understanding of its mechanism of action, with its efficacy derived from the pivotal DEN-301 study [39]. As in any dynamic model, the probability of a susceptible individual becoming infected at any one point in time depended on the number of infectious individuals in the population, allowing for the simulation of indirect effects of vaccination [52].

For this model, epidemiological data values were informed by data specific to Thailand where appropriate.

### Host and vector populations

The host population in the model was stratified into 1-year age cohorts, for ages 0–100 years. Consistent with previous models in this field [53,54], the population size and age structure were assumed to remain constant over time. The inflows into and outflows from the country population due to migration and travel were not considered. All demographic processes (births, aging, and all-cause deaths) were modeled as discrete events occurring once per year. Dengue-caused deaths averted with vaccination were considered in the estimation of cost and quality-of-life burden, but not in the epidemiological model, as their impact on the total population size and dynamics of dengue transmission was assumed to be negligible.

The vector population in the model included adult female mosquitoes only, as only females transmit the virus through bites [4]. The average vector population size throughout the year was assumed to be twice the size of the host population, based on epidemiological studies [55–58]. Vectors were assumed to only have 1 infection in their lifetime, due to their short life expectancy. It is thought that seasonality may play a role in dengue transmission, as the number of vectors tends to increase during rainy seasons [59]. Therefore, to simulate seasonal fluctuations, a sine forcing function was applied to the vector emergence rate. This function was parametrized by fitting the model output to the observed incidence of dengue cases during each calendar month (S1 and S2 Files).

### Infection process and epidemiological outcomes

The infection process (i.e., the acquisition and transmission) was modeled by simulating the interactions between the hosts and vectors and the corresponding flows between the model compartments. The hosts were assumed to be born susceptible to all 4 dengue serotypes. When bitten by an infectious vector, a susceptible host may become infected with the serotype the vector was carrying (Fig 1A). The probability of the bite being effective (i.e., resulting in a dengue infection) was varied by age and was obtained by fitting the model to the age-specific incidence rate of symptomatic dengue in the country of interest (in this case, Thailand). Similarly, the adult female vectors were assumed to be susceptible to all 4 serotypes when entering the model (Fig 1B). They could then be infected with a dengue serotype if feeding on a host infected with this serotype. Following an incubation period, the vector could transmit the serotype in question onward to another susceptible host, in line with known epidemiological data [51,60,61].

The model did not include explicit compartments for the infections of different severity in the hosts. Instead, the probabilities of being symptomatic, hospitalized, developing severe disease, and dying were applied to the predicted number of infections (Fig 1C). The present analyses did not account for potential variation of the probability of clinical disease or hospitalization by age at infection or by infecting serotype. In line with the majority of the previously published dengue models, the probability of symptomatic and hospitalized dengue was differentiated for first, second, and subsequent infections, with more severe outcomes being the most probable in second infections [62].

Several studies suggest that symptomatic infections may be associated with a higher viral load, which in turn may be associated with greater transmissibility [63–65]. In the base case analyses, symptomatic infections were assumed to be 2 times more transmissible than asymptomatic infections, consistent with assumptions used in previously published dengue models [54]. The impact of changes in this assumption was explored in scenario analyses (S3 File).

The public health impact of vaccination was assessed in terms of the number and proportion of cases avoided, with granular results for symptomatic, hospitalized, severe, and fatal cases. In the base case analyses, the results were assessed over a 20-year timeframe, to fully capture the long-term impact of vaccination. The key input parameters used in the epidemiological model are listed in Table 1.

**Table 1:** Epidemiological parameters used in the model.

Parameter	Base case value	Source
Number of adult female vectors per host	2	Entomology studies [56–58]
Vector life expectancy	14 days	Entomology studies [66,67]
Duration of latent period in vectors	10 days	Entomology studies, modeling studies [60,61,66]
Vector biting rate	0.7 per day	Modeling study [68]
Amplitude of the seasonal sine function	0.34	Fitted^a^
Horizontal shift of the seasonal sine function	–0.51	Fitted^a^
Probability of virus transmission from an infectious host to a susceptible vector, given a bite	30%	Modeling study [66]
Probability of virus transmission from an infectious vector to a susceptible host, given a bite	0.1%–29.4% (age specific)	Fitted^a^
Duration of latent period in hosts	5 days	Entomology studies, modeling studies [51,66,69]
Duration of viremia in hosts	4.5 days	Entomology studies, modeling studies [51,59,70–72]
Probability of symptomatic disease with primary infections	30.0%	Review of modeling studies [62]
Probability of symptomatic disease with secondary infections	60.0%	Review of modeling studies [62]
Probability of symptomatic disease with tertiary and quaternary infections	10.0%	Review of modeling studies [62]
Probability of hospitalization with symptomatic primary infection	15.9%	Fitted^a^
Probability of hospitalization with symptomatic secondary infection	30.0%	Fitted^a^
Probability of hospitalization with symptomatic postsecondary infection	7.5%	Fitted^a^
Proportion of severe infections among hospitalized infections	39.4%	Calculated^a^
Probability of dengue-caused death given a hospitalization for severe dengue	0.26%	Estimated from the Thailand surveillance data^a^
Cross-protection duration	6 months	Modeling studies [59,73–75]
Transmissibility of symptomatic infections (relative to asymptomatic)	2	Modeling studies [54]

^a^More details in S1 and S2 Files.

### Impact of vaccination

Vaccination with TAK-003 was assumed to provide protection against both symptomatic and asymptomatic dengue. The level of protection was derived from clinical trial data, and for the protection against symptomatic dengue it was assumed to be boosted back to the original postvaccination level (or higher) with each breakthrough infection (natural boosting; see S2 File).

### Determination of TAK-003 efficacy

The level and duration of protection offered by TAK-003 were estimated based on the final 54-month post-second dose data [42] from the pivotal clinical trial on TAK-003 (DEN-301) [39]. Although some decline in the efficacy against overall VCD was observed by the end of the follow-up period (4.5 years post second dose), the efficacy against hospitalized VCD remained relatively stable over time [76].

**Fig 2:**
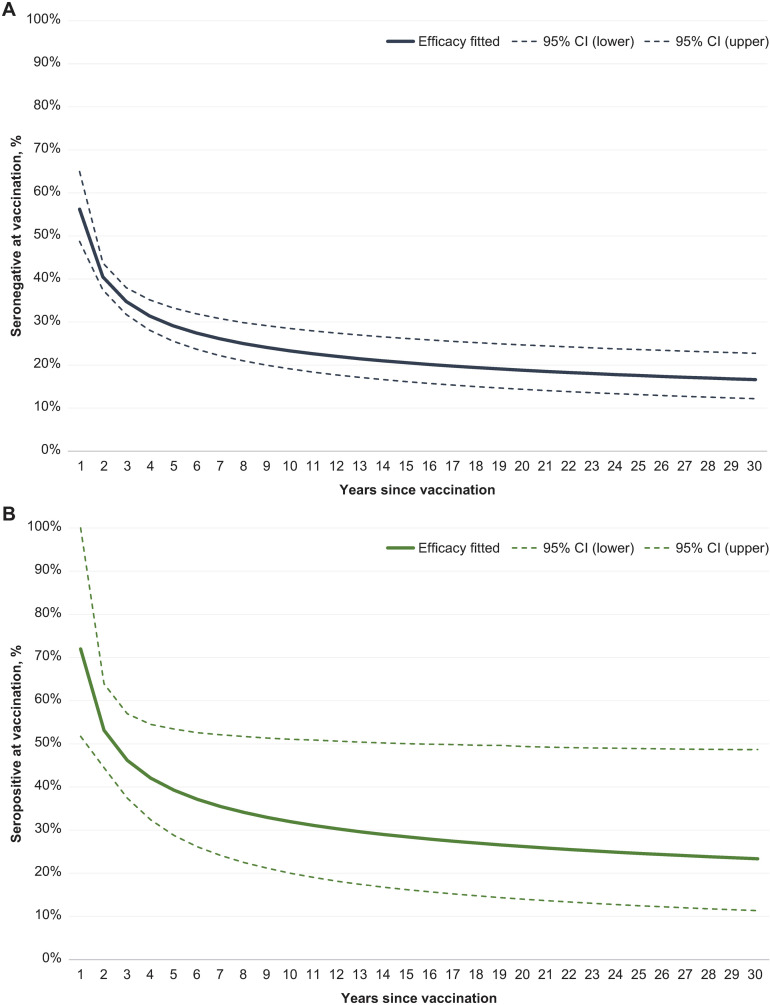
Vaccine efficacy against nonhospitalized dengue, by serostatus at vaccination. **(A)** Seronegative at vaccination; **(B)** seropositive at vaccination. CI, confidence interval.

Due to the differential waning of overall VCD and hospitalized VCD, and given that hospitalized VCD was a subset of overall VCD, to avoid double counting, post hoc analyses were undertaken to calculate the efficacy of TAK-003 separately for symptomatic nonhospitalized infections and symptomatic hospitalized infections. For nonhospitalized infections, the efficacy of TAK-003 was estimated for each of the 4 years following the second dose (the last half year was not used, as it may not be representative of the entire year due to seasonal variations) and extrapolated beyond the trial duration by fitting a power trend to the yearly efficacy estimates, separately by serostatus at vaccination (Fig 2).

For hospitalized infections, the efficacy of TAK-003 was assumed to be constant over time and independent of the serostatus at vaccination, as the final results of DEN-301 indicated very minor differences across years and subgroups [42].

In addition, efficacy against asymptomatic infections was included in the model and supported by post hoc analyses of the immunogenicity subset of the DEN-301 trial [77]. These analyses defined an asymptomatic infection as a 4-fold increase in neutralizing antibody titer and a titer ≥40. This definition was in line with the definition used in Olivera-Botello and colleagues [78], based on which Coudeville and colleagues [79] assumed the efficacy of the CYD-TDV vaccine against asymptomatic infection to be half of the efficacy against symptomatic infection. In the present model, a similar approach was taken, with the efficacy against asymptomatic infection assumed to be half of the efficacy against nonhospitalized symptomatic infection. Additionally, scenario analyses were performed assuming no efficacy against asymptomatic dengue.

The vaccine efficacy applied in the model was differentiated by serostatus, but not differentiated by serotype. Assessment of efficacy by serostatus was a secondary objective in the DEN-301 trial [39] while efficacy by serostatus and serotype was assessed for use in an exploratory scenario.

### Natural boosting

Current evidence suggests that exposure to DENV may induce an immune response that can be protective against symptomatic disease [80,81]. Thus, a breakthrough dengue infection (i.e., an infection following vaccination) may also boost vaccine-induced immune response. This hypothesis of natural boosting of vaccine-derived protection is applied in the model. For seropositive recipients, each breakthrough infection (symptomatic or asymptomatic) was assumed to bring the level of vaccine protection back to the same level as immediately post vaccination. For seronegative recipients, the level of protection, following each breakthrough infection, was boosted to the same level as those who were seropositive at the time of vaccination. A detailed description of the methods applied to model the natural boosting of vaccine efficacy is provided in S1 File.

### Model fitting

The model was designed to be adaptable to dengue-endemic countries and regions. In this study, the model was fitted to the epidemiology of dengue in Thailand by using 2 sets of reported case counts (by age and by calendar month). The former was used to calibrate the values of the probability of virus transmission from an infectious vector to a susceptible host, given a bite, with the relative size of each age cohort calculated using recent national statistics data from Thailand [82]. The latter was used to calibrate the amplitude and horizontal shift of the seasonal sine function applied to the emergence rate of adult female vectors. The case counts, as reported by the Ministry of Health in Thailand [83], were converted into incidence rates and averaged over the period 2011–2020, which was assumed to be representative of the long-term average dengue transmission. As it was not possible to predict future circulation of dengue serotypes, in the forward simulations the 4 serotypes were assumed to co-circulate and distribute equally on average over the long term (although at any short-term period, a dominance of 1 or 2 serotypes would be observed as described in the “Results” section). A detailed description of the methods for model fitting is provided in S2 File.

**Fig 3:**
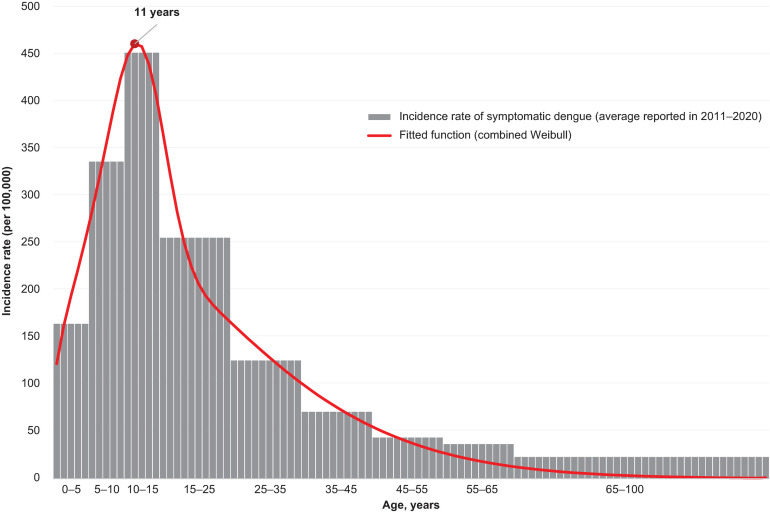
Incidence rate by age, grouped and single year. The gray columns represent the incidence rate of reported symptomatic dengue (average for each age group and average over the period 2011–2020). The red line represents the curve that was fitted to the reported data to smoothen them.

The incidence data were preferred to seroprevalence data for model fitting because, although seroprevalence studies may be a valuable source to inform the age-specific acquisition of dengue infection, they do not allow for the capture of specific patterns of age distribution of symptomatic cases, which are visible in the incidence data (Fig 3). Ideally, the 2 types of data should be used in combination to inform the acquisition of both symptomatic and asymptomatic dengue. However, seroprevalence studies are not always available or generalizable, and in this case, no robust seroprevalence studies were identified for Thailand at the national level. However, the seroprevalence predicted by the model fit within the range of seroprevalence for Thailand found in the literature [84,85].

The aggregate case counts reported by the Ministry of Health in Thailand [83] were provided for 9 age groups of variable length, with the highest incidence observed between age 10 and 14 years (Fig 3). The potentially arbitrary age grouping meant that the values of calibrated parameters (and thus predicted incidence rates) changed dramatically at the beginning of each age group. The use of incidence data grouped by age was also found to bias the results of the cohort optimization analysis, as the highest burden of symptomatic dengue was always placed on the first year of the age group with the highest overall incidence. To overcome this limitation, dengue incidence by single year of age was approximated by using interpolation and approximation techniques, illustrated in Fig 3 and described fully in S2 File.

It is generally recognized that the cases reported in passive surveillance only represent a fraction of true dengue cases [46,86–88]. To account for this discrepancy, data from Wichmann and colleagues [46] were used, which estimated the expansion factor (underestimate to be factored for) for dengue cases in Thailand at 8.37. This was used in the model fitting to correct for underreporting.

### Vaccination strategies

Dengue affects all ages, and the age distribution of dengue cases may vary significantly by region [89]; therefore, there is no single vaccination strategy suitable for all countries and the optimal age of dengue routine vaccination should be based on the country’s epidemiological profile. Furthermore, given the broad impact of dengue across all ages, a catch-up vaccination campaign, whereby a wider cohort of people are immunized in the year of vaccine introduction, may be of particular importance to reduce transmission at a faster speed and result in a more notable reduction of cases.

We first explored what are the optimal routine and routine plus catch-up cohorts for Thailand, under “cohort optimization,” and secondly, we examined a “pragmatic” strategy, which allowed for practical considerations through integration into an existing vaccination schedule—in this case, the Thai NIP—to potentially maximize vaccination coverage and reduce administrative costs.

### Cohort optimization

In the incidence curve with the best fit to the reported data, the peak of incidence was observed at age 11 years (Fig 3), which was used to define the upper limit of the age range explored for routine vaccination. The lower limit was set to 4 years as the youngest indicated age for TAK-003 [41]. In addition to the vaccination strategies with routine vaccination alone, 2 sets of strategies with catch-up vaccination were explored: vaccination of 5 and 10 additional cohorts.

**Fig 4:**
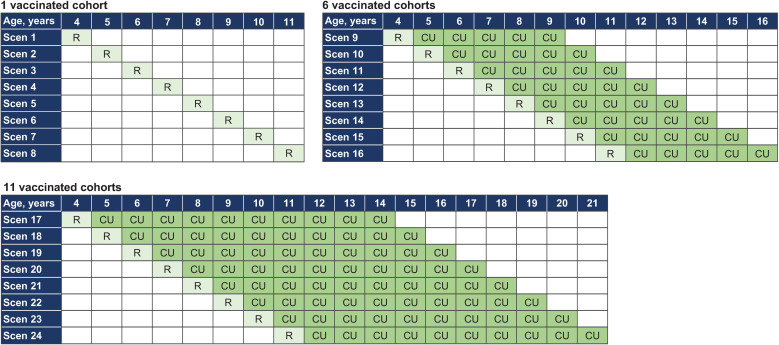
Scenarios explored in the cohort optimization analyses. CU, catch-up vaccination (multiple years vaccinated as a one-off in the first year of introduction); R, routine vaccination (single age cohort then administered annually thereafter); Scen, scenario.

The full range of ages selected for routine vaccination (i.e., 4–11 years) was explored in all 3 sets of analyses. The scenarios explored for the cohort optimization are illustrated in Fig 4. The coverage for these analyses was assumed to be 80%, in line with assumptions made in previous models [62,90,91].

### Pragmatic scenario

A “pragmatic” scenario was simulated to assess the impact of TAK-003 as a strategy that could be introduced into Thailand. This strategy involved routine vaccination at age 11 years, to be administered at the same time as the HPV vaccination on the Thai NIP schedule. As with the cohort optimization strategies, fitted single-year incidence data were used. However, to reflect the potential real-world implementation of this strategy, vaccine coverage of 87% was used (equivalent to HPV coverage) [92,93].

### Cost-effectiveness analysis

The cost-effectiveness of the vaccination strategies versus no vaccination was evaluated from a societal perspective, accounting for the broader impact of dengue disease on patients, their families, and society as a whole [94]. A payer perspective was explored in both the cohort optimization scenarios and the pragmatic scenario (S3 File).

For costs, the model estimated the direct medical and nonmedical cost of each dengue case, differentiating between hospitalized mild, hospitalized severe, and nonhospitalized cases. Indirect costs included costs of patient and caregiver workdays lost due to dengue cases, productivity loss cost due to dengue-caused death, and school absenteeism costs. This was a societal perspective that is recommended by Thai guidelines for health technology assessment (HTA) [95]. An illustrative price of US$30 per dose was used in the cost-effectiveness analysis. The vaccine administration cost was assumed to be US$2.30 based on the cost-effectiveness analysis by Meeyai and colleagues [96].

The humanistic burden of dengue was measured in terms of disability-adjusted life-years (DALYs) [97–99]. Disability weights and duration for severe, mild, and persistent dengue required for the estimation of DALYs were taken from the literature [26,100,101]. Years of life lost to premature dengue-caused death were based on age at death and age-specific life expectancy in Thailand. The incremental cost-effectiveness ratio was calculated using a willingness-to-pay (WTP) threshold of US$7,000, which is equivalent to approximately 1 gross domestic product (GDP) per capita in Thailand [102]. This is in line with Thai HTA guidelines, where using GDP per capita as the WTP threshold was recommended [95]. Costs and DALYs were discounted to present values at 3% per year [103], in line with Thai HTA guidance [95]. Full information on costs and DALYs is reported in S2 File. Reporting guidance for economic evaluations was adhered to using the Consolidated Health Economic Evaluation Reporting Standards (CHEERS) 2022 checklist and is reported in S1 File.

### Sensitivity analyses

Sensitivity analyses were conducted to test the overall robustness of the model and results. These included varying assumptions about the epidemiology of the disease and the efficacy of the vaccine. Where appropriate, their impacts on both epidemiological (cases of dengue and hospitalizations avoided) and cost-effectiveness results were explored (S3 File).

The exact year of vaccine introduction may have an important impact on the results due to potentially considerable year-to-year variations of dengue incidence. To account for the uncertainty around the initial condition, 70 forward simulations were run with varying years of vaccine introduction, with the results presented in this manuscript reflecting the average of these simulations.

## Results

### Epidemiology of dengue in the absence of vaccination

**Fig 5:**
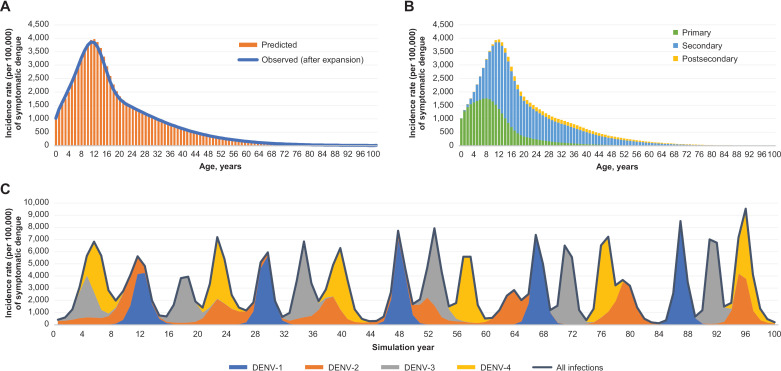
Results of simulation without vaccination. **(A)** Incidence rate (per 100,000) of symptomatic dengue, by age. **(B)** Incidence rate (per 100,000) of symptomatic dengue, by age and type. **(C)** Incidence rate (per 100,000) of dengue infection, by serotype and simulation year. The observed rate is based on the Weibull curve fitted to the reported incidence rate of symptomatic dengue (as described in the “Model fitting” section in the “Methods” and in Fig 2); underreporting is accounted for by applying an expansion factor of 8.37. In panels **A** and **B**, the incidence rate represents the average rate of symptomatic dengue predicted by the model in the last 100 years (following a 650-year burn-in period) simulated in the process of model fitting with the best-fit values of the fitted parameters. In panel **C**, the incidence rate represents the rate of dengue infection (symptomatic or asymptomatic) predicted by the model in the next 100 years. DENV, dengue virus.

The model-predicted incidence rate of symptomatic dengue in Thailand without vaccination was a good fit to the empirical data (Fig 5A). The majority of symptomatic cases were due to either primary or secondary dengue infection, with only a small proportion of cases being due to postsecondary infections (Fig 5B). As has been observed in real life, the predicted dengue incidence is characterized by a high degree of year-to-year variations in infection rates (Fig 5C). Despite the observed regular changes in the dominant serotype (Fig 5C), the 4 serotypes in each 20-year forward simulation were almost evenly distributed. The predicted seroprevalence at age 11 years was 59.6% (Fig A in S3 File), which was consistent with observational data reported from Thailand (59.7% in children aged 10–14 years) [85]. The model also showed a reasonably good fit to seasonality (Fig C in S3 File).

Without vaccination, the dynamic model predicted approximately 41.3 million dengue infections in Thailand over a 20-year period (Table 2). Of those, 14.6 million (35%) infections would be symptomatic and 3.4 million (8%) resulted in hospitalization.

**Table 2:** Number of infections and infections avoided for no vaccination and for R11.

Strategy	Total infections	Asymptomatic	Symptomatic cases	Hospitalized cases	Nonhospitalized cases	Dengue-caused deaths
No vaccination	41,273,513	26,718,297	14,555,216	3,425,852	11,129,364	3,500
R11	26,179,217	18,020,095	8,159,122	1,626,637	6,532,485	1,662
Number avoided	15,094,296	8,698,202	6,396,094	1,799,215	4,596,879	1,838
Proportion avoided	37%	33%	44%	53%	41%	53%

R11 strategy is the routine vaccination at age 11 years without catch-up. Data presented are at the population level of Thailand over a 20-year time horizon with no discounting.

### Cohort optimization

#### Impact on dengue.

**Fig 6:**
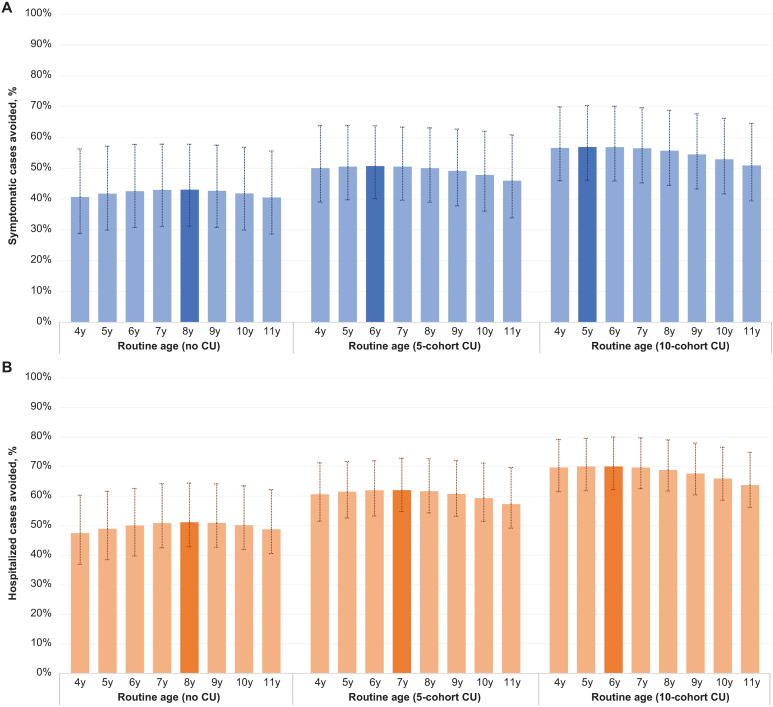
Proportion of cases avoided in each vaccination strategy explored in the cohort optimization analyses (over 20 years). **(A)** Symptomatic cases avoided and **(B)** hospitalized cases avoided. The columns represent the mean proportions of cases avoided; the vertical lines represent the 95% confidence intervals of all model realizations. All vaccination strategies were simulated assuming 80% coverage in all vaccinated cohorts. Each column represents the outcome of a given vaccination strategy with the routine vaccination at a specific age and with a specific number of age cohorts vaccinated as part of CU vaccination in the first years of vaccine introduction. For example, the column “4y” in the cluster “Routine age (5-cohort CU)” represents the results of the strategy with annual routine vaccination of children aged 4 years and CU vaccination of children aged 5–9 years. The strategy with the greatest proportion of cases avoided (in the respective cluster) is highlighted with a darker color. These results are presented in tabular form in S2 File. CU, catch-up.

All the 24 vaccination strategies simulated as part of the cohort optimization analyses demonstrated a considerable public health impact, with 41%–57% of symptomatic cases and 47%–70% of hospitalized cases avoided over a 20-year timeframe (Fig 6). The public health impact of TAK-003 was dependent on the size of the catch-up campaign, with more cohorts vaccinated as part of catch-up vaccination resulting in more symptomatic and hospitalized cases avoided. Secondary to this, the optimal age of routine vaccination varied with the size of catch-up, with the larger the catch-up cohort, the earlier the optimal routine age being observed. When no catch-up was applied, the optimal age of routine vaccination was 8 years. When 10 years of catch-up was implemented, the optimal age was lowered to 5–6 years (prevention of symptomatic infection and hospitalizations, respectively).

#### Cost-effectiveness.

**Fig 7:**
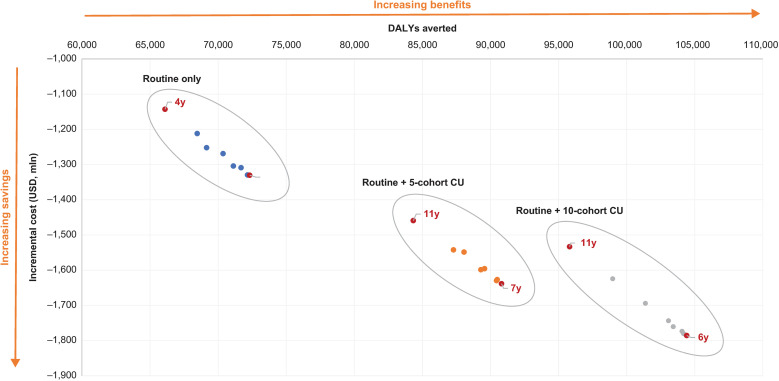
Cost-effectiveness plane for 24 vaccination strategies explored in the cohort optimization analysis (SE quadrant). The total cost includes all the subcategories displayed in Tables 3 and 4 (S3 File) and reflects the societal perspective. The incremental costs are calculated vs. no vaccination; a negative incremental cost indicates that the vaccination strategy is less costly. The DALYs averted are calculated vs. no vaccination; a positive number indicates that the vaccination strategy is more effective than no vaccination. Both costs and DALYs are discounted at 3% per annum. These results are presented in tabular form in S3 File. DALY, disability-adjusted life-year; CU, catch-up; mln, million; SE, southeast; USD, US dollars.

All explored strategies were dominant versus no vaccination from the societal perspective under an assumed vaccine price of US$30 per dose. All the results were located in the southeast quadrant of the incremental cost-effectiveness plane (Fig 7), indicating that regardless of the specific strategy, TAK-003 was dominant over the no vaccination strategy (lower costs and greater benefits). The most cost-effective strategy was routine vaccination in children aged 6 years with 10 years of catch-up, which was associated with 104,415 fewer total DALYs and savings of US$1,786 million.

The threshold analysis showed that the maximum price for vaccination to be dominant compared with no vaccination was between US$74 and US$93 per dose, and between US$93 and US$117 per dose for vaccination to be cost-effective with a WTP threshold of US$7,000 (approximately 1 GDP per capita in Thailand [104]) per DALY averted.

### Pragmatic scenario (routine vaccination of 11 year olds)

#### Impact on dengue.

**Fig 8:**
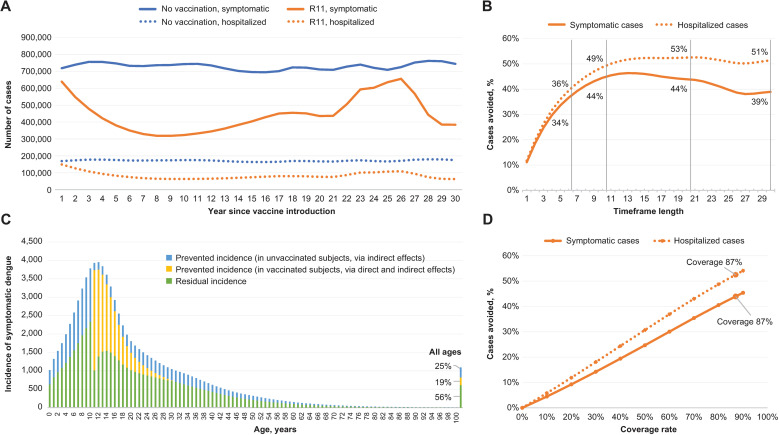
Public health impact of the R11 strategy in Thailand. R11 strategy is routine vaccination at age 11 years without catch-up. **(A)** The absolute changes in the number of symptomatic (solid lines) and hospitalized (broken lines) cases over a 30-year period for both the vaccination and no vaccination strategies. **(B)** The relative change in cases over this period, compared with no vaccination. **(C)** Incidence of symptomatic dengue, over 20 years; this panel illustrates a breakdown of the effect of TAK-003 in terms of direct and indirect effects, as well as residual cases (cases that occur despite R11 vaccination). **(D)** Cases avoided with R11 over 20 years; this panel illustrates the linear impact of coverage rates on cases avoided (symptomatic and hospitalized).

A potential pragmatic scenario was modeled where TAK-003 was co-administered with the existing HPV program in Thailand, in which routine HPV vaccination of children aged 11 years has been estimated to have 87% coverage. This scenario resulted in the prevention of 44% of symptomatic cases and 53% of dengue hospitalizations compared with no vaccination over a 20-year period (Table 2). The predicted numbers of symptomatic and hospitalized dengue cases are shown in Fig 8A, and the cumulative proportions of symptomatic hospitalized cases avoided using routine vaccination R11 compared with no vaccination program are shown Fig 8B.

Over a 10-year timeframe (the time horizon often used in the other dengue models [70,75,90,105]), the public health impact of R11 was also estimated to prevent 44% of symptomatic cases compared with no vaccination (Fig 7B). A moderate rebound effect can be observed with the proportion of cases and hospitalizations avoided, peaking at around 15–20 years following the introduction of vaccination, and then reducing slightly [62].

#### Indirect protection.

The implementation of TAK-003 resulted in pronounced indirect protection in both the vaccinated and unvaccinated populations (Fig 8C). These indirect effects were driven by 2 mechanisms. Firstly, the assumed vaccine efficacy against asymptomatic dengue leads to a reduction in the overall number of infections, reducing the force of infection. Secondly, asymptomatic infections are assumed to be less transmissible than symptomatic infections, whereas vaccinated individuals also have a lower chance of having symptoms when infected with dengue. As a result, infections in vaccinated individuals are relatively less infectious to mosquitoes who feed on them, thus reducing the overall transmission intensity.

#### Impact of coverage rates.

The impact of vaccination coverage rate was explored for the R11 strategy as a scenario analysis (Fig 8D). Higher coverage led to consistently better disease reduction, with an almost linear relationship observed between the coverage rate and the proportion of cases averted.

#### Additional scenario analyses.

**Fig 9:**
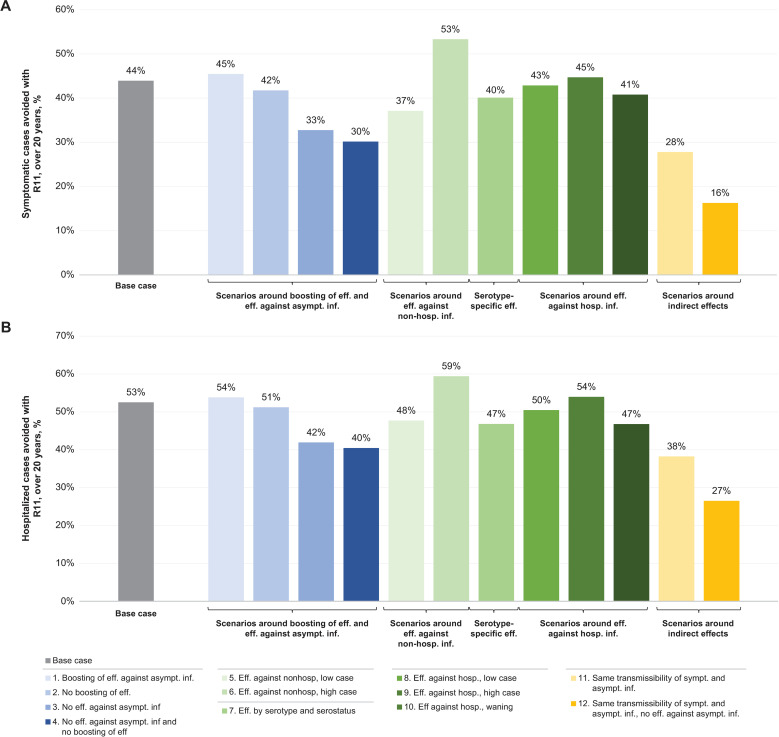
Scenario analyses around the proportion of cases avoided with R11, over 20 years. **(A)** Symptomatic and **(B)** hospitalized cases avoided with R11, over 20 years. Scenario analyses on the R11 pragmatic simulation. Scenarios 1–4 assessed changes to assumptions around boosting and efficacy against asymptomatic dengue, scenarios 5–10 explored sensitivity of the results to changes in TAK-003 efficacy, and scenarios 11–12 assessed changes to transmissibility assumptions. For full details, see S3 File.

Eleven additional scenarios were conducted around the R11 pragmatic vaccination strategy, exploring changes to assumptions and parameters of the epidemiology of the disease and the efficacy of TAK-003. These are reported in full in S3 File and are summarized graphically in Fig 9. All the scenarios demonstrated the benefit of R11 strategy in preventing both symptomatic cases and hospitalization by varying degrees. For symptomatic cases avoided, 3 scenarios predicted increased protection with TAK-003 (compared with base case), with the assumption of high case efficacy against nonhospitalized dengue reporting the highest simulated reduction (53%). The lowest proportion of cases and hospitalizations averted was predicted in a scenario with equal transmissibility of symptomatic and asymptomatic infections and with no protection against asymptomatic infections (i.e., a scenario in which vaccination would not generate any indirect effect). A similar pattern was observed with episodes of hospitalization avoided.

#### Cost-effectiveness analyses.

Over 20 years and at a discount rate of 3%, vaccination with TAK-003 was found to be cost saving and led to fewer DALYs (i.e., greater health benefits) compared with no vaccination (Table 3). The R11 strategy thus dominated the no vaccination strategy. Threshold prices for cost savings and cost-effectiveness with a WTP threshold of US$7,000 were calculated. From a societal perspective, the maximum price of 1 vaccine dose was estimated at US$89 for dominance, and at US$111 for cost-effectiveness under the WTP thresholds of US$7,000. The vaccine threshold prices were higher for cost-effectiveness than for dominance, as a monetary value was attributed to each DALY averted. In summary, the threshold pricing analysis has demonstrated the high economic value of TAK-003 in the NIP to prevent dengue in Thailand.

**Table 3:** Total and incremental costs and DALYs for R11 over 20 years (discounted at 3%). Costs are reported in million US dollars.

Strategy	Direct medical cost	Direct nonmedical cost	Productivity loss	Cost of school absence	Cost of vaccine and administration	Total cost	Total DALYs
No vaccination	2,831	364	1,103	57	0	4,355	162,458
R11	1,443	193	604	27	743	3,009	87,715
Incremental (R11 vs. no vaccination)	–1,388	–171	–499	–30	743	–1,346	–74,744

The total cost includes all the subcategories displayed in the table and reflects societal perspective. The incremental costs are calculated versus no vaccination; a negative incremental cost indicates that the vaccination strategy is less costly. The incremental DALYs are calculated versus no vaccination; a negative number indicates that the vaccination strategy is more effective than no vaccination.

DALY, disability-adjusted life-year.

## Discussion

TAK-003 was found to be highly efficacious at preventing symptomatic and severe episodes of dengue and hospitalizations in the DEN-301 trial [39]. The current study used a dynamic transmission model of dengue designed to simulate the impact of vaccination with TAK-003. Because the epidemiology of dengue varies widely between countries and regions due to different demographic, socioeconomic, climatic, and geographical factors [50,106], dengue vaccination strategies should not be implemented in individual countries as “one size fits all,” but instead should be tailored to the country-specific dengue epidemiological profile [44]. In this study, we first assessed candidates for the optimal dengue vaccination strategies for Thailand, and then we explored a pragmatic strategy that could be potentially incorporated into the Thai NIP to achieve maximum coverage. The key results were as follows.

Firstly, TAK-003 was found to reduce the number of dengue cases in all the scenarios explored, compared with no vaccination. This reflects the high efficacy of the vaccine, against both symptomatic cases and hospitalization.

Secondly, the addition of catch-up cohorts was associated with a large increase in the reduction of dengue cases (both symptomatic and hospitalized). By allowing vaccination of a larger initial cohort in the year of vaccine introduction, catch-up had an immediate impact on transmission, although the efficiency of the catch-up program may decrease with increasing catch-up sizes. Enlarging the size of the catch-up cohort also impacted the optimal age of routine vaccination, with increasing catch-up cohort sizes being associated with lower ages for optimal routine vaccination. This is because the underlying risk of symptomatic or hospitalized dengue is considerably lower in people who already have a history of 2 or more infections; therefore, when the catch-up age range is expanded to include the optimal age of vaccination (found to be 8 years in the absence of catch-up), this tends to lower the optimal age of routine vaccination. If a 10-year catch-up strategy was to be implemented, we estimate that the optimal routine age of vaccination in Thailand would be between 5 and 6 years.

The third key result pertained to the cost-effectiveness of TAK-003. At the illustrative cost of US$30 per dose, TAK-003 was always associated with increased health benefits and reduced societal costs (Fig 7). As a consequence of the cost-saving nature of the intervention, one inference that can be drawn for policy makers is that the larger the catch-up cohort size that is implemented, the greater the long-term economic and health benefits there will be for the country, and potentially the greater the cost savings. However, these results come with the caveats that there was evidence of diminishing economic returns associated when 10 years of catch-up was applied, and, on a practical level, catch-up campaigns would be subject to the budgetary constraints of the country.

Finally, given these caveats, we also investigated a scenario whereby TAK-003 was co-administered with HPV in the Thai NIP at 11 years, where empirical data has shown an achievable coverage rate of 87% [92,93]. Using this strategy, it was observed that 44% of symptomatic cases and over half of hospitalizations could be avoided over a 20-year timeframe. In this pragmatic scenario, TAK-003 was dominant compared with no vaccination, and we estimate that its implementation in Thailand could lead to long-term cost savings equivalent to >1 billion US dollars, while at the same time relieving much of the mortality and morbidity associated with the disease. Although catch-up programs were not explored in this pragmatic strategy, it is reasonable to expect a comparable magnitude of impacts based on the cohort optimization analysis with catch-up programs of 5 or 10 age cohorts.

To our knowledge, this was the first study to assess the public health impact and cost-effectiveness of TAK-003 in Thailand. Early epidemiological models were developed before effective vaccines were available and did not include all serotypes [59]. Later, more sophisticated models were developed. The archetypal study the current model was based on modeled human and mosquito populations in Southern Vietnam and assessed the impact of a hypothetical tetravalent vaccine [51]. Subsequent models have been developed to assess the impact of the first licensed dengue vaccine, CYD-TDV [79]. The benefits associated with CYD-TDV were considerably lower than those reported with TAK-003 demonstrated in the present study, and this trend has also been observed in other models on CYD-TDV [62]. We consider that the improved clinical and economic benefits observed with TAK-003 compared with CYD-TDV in these models reflect fundamental differences between the vaccines. The primary clinical evidence has shown that TAK-003 is effective in both seronegative and seropositive individuals [39], without the safety issues identified with CYD-TDV [32]. This means TAK-003 can be used to protect a broader population (both seropositive and seronegative individuals) compared with CYD-TDV. Furthermore, as a consequence, TAK-003 eliminates the need for prescreening that is required for CYD-TDV, which poses a material challenge in terms of the feasibility of vaccine rollout, public communication, and service provision [107].

Recently, WHO published a position paper on dengue vaccines in which they recommend the introduction of TAK-003 in countries with high dengue endemicity without screening for serostatus [44]. The WHO recommends that countries should consider data on age-specific seroprevalence and/or age-specific dengue hospital admissions that indicate there is a high risk of dengue transmission. The WHO’s position paper was supported by Bayesian modeling and an analysis conducted for the Strategic Advisory Group of Experts on Immunization [108], which had methodological differences compared with the current study, such as using a risk-benefit analysis and favoring generalized seroprevalence over specific incidence data for model fitting. However, overall, WHO recommendation to use TAK-003 in children aged 6–16 years in settings with high dengue transmission intensity is supportive of the findings of the current study and the introduction of the vaccine into affected regions of Thailand.

The current study was likely to have reported a conservative estimate on the cost-effectiveness of TAK-003, as not all dengue-related costs could be incorporated into the model. As with other economic analyses, the current study focused on the direct and indirect costs of dengue cases, with the principal driver being direct medical costs. However, the impact of dengue extends beyond this, and the study did not include all relevant broader costs, including macroeconomic impact, such as costs to Thailand’s tourism industry. For instance, in 2019, a surge of dengue cases had a profound impact on tourism, reducing GDP by an estimated US$1.81 billion, equivalent to 3% of annual tourism revenues or 0.33% of the total GDP [109]. Overall, dengue has been reported as having a large impact on the GDP of Asian countries [110], which cannot be fully captured in the model. Furthermore, the burden of dengue may disproportionately affect the most disadvantaged people in society due to a lack of access to healthcare and other preventive resources. Inclusion of TAK-003 into the NIP could therefore help to address the inequalities of dengue burden within the population.

In summary, the results of this study have shown that vaccination programs with TAK-003 can result in a reduction of dengue in Thailand and were cost saving compared with no vaccination. Sensitivity analyses demonstrated that the results are robust to underlying assumptions about the epidemiology of dengue and the efficacy of TAK-003. The benefits of the vaccine can be maximized by determining the optimal age cohorts for vaccination based on the country-specific epidemiology and by introducing catch-up vaccination programs.

### Strengths and limitations

The model had several strengths. Firstly, it was developed from a previous study on dengue fever that was peer reviewed and validated [51]. The current model was then fitted to recent and detailed data available for Thailand and populated with the most reliable inputs and parameters identified in the literature. Secondly, the efficacy data used for the intervention were derived from a large, phase 3, randomized, placebo-controlled trial of TAK-003 (DEN-301) [39]. This trial was adherent to all the parameters set by WHO dengue clinical guidelines trial methodology [40]. Thirdly, unlike most other models on dengue vaccination [62], the model implemented the mechanism of natural boosting of vaccine-derived protection, more closely reflecting the immunological response to infection, which was reported to be fundamentally different following secondary infections compared with primary infections [111,112]. Fourthly, a societal perspective was used for the cost-effectiveness analysis, which takes into consideration the broader economic impact of dengue and its prevention. Finally, although model size and runtime did not allow for comprehensive probabilistic sensitivity analyses, the key parameters related to vaccine efficacy and mechanism of action were tested in scenario analyses, increasing confidence in the robustness of the model results.

Nevertheless, the model had some limitations. Firstly, we did not explicitly simulate demographic characteristics of Thailand and demographic shift (i.e., changing birth and death rates), and the effects of migration were not accounted for. However, similar simplifying assumptions are often found in other dengue models [53,54]. Secondly, as discussed in the “Methods” section, the vaccine efficacy applied in the base case analyses was differentiated by serostatus, but not differentiated by serotype. To address this limitation, a scenario applying exploratory serotype- and serostatus-specific efficacy was conducted. The results of this analysis indicated a slight decrease in the public health impact (the proportion of symptomatic cases averted reduced from 44% to 40% over 20 years); the base case vaccination strategy remained dominant compared with no vaccination. Thirdly, we did not account for the practical considerations of catch-up strategies, including issues with service delivery and organization, as well as issues with coverage outside of NIPs. Other logistical details, such as maintaining the cold-chain supply, were not considered. Fourthly, the model size and runtime meant it was not computationally possible to run probabilistic sensitivity analyses. To mitigate this limitation, scenario analyses were run addressing the most contentious of the model assumptions (specifically, the assumptions related to the vaccine efficacy extrapolated over the trial duration, protection waning, natural boosting of vaccine efficacy, and achievable vaccination coverage). The results indicated that even in the most pessimistic scenarios, the routine vaccination of children aged 11 years was dominant compared with no vaccination. Finally, the pragmatic strategy selected was based on integration with the HPV vaccination of the NIP at age 11 years. TAK-003 may be administered concomitantly with a HPV vaccine in Thailand and coadministration has been studied in participants aged 9–14 years [113]. However, although HPV vaccination is recommended for both sexes internationally [114], only girls are currently eligible for this vaccine in Thailand [115]. Additionally, the HPV vaccine is usually administered as 2 doses 6 months apart [115], which is different from the 3-month schedule of TAK-003 [41]. Therefore, the coverage used in the analysis [93] may have been optimistic.

This study presents the results of a dynamic transmission model that simulated the epidemiology of dengue and the impact of TAK-003 in Thailand, with a focus on optimizing the cohort age for vaccination strategy to maximize impact. The analysis found that in all scenarios, vaccination with TAK-003 led to reductions in both symptomatic and hospitalized dengue. The impact could be increased by implementing catch-up programs. At an illustrative cost of US$30 per dose, TAK-003 was found to be dominant in all scenarios and vaccination strategies analyzed, resulting in both large health gains and notable cost savings, compared with no vaccination. The threshold analysis indicated that TAK-003 would remain cost-effective in excess of US$100 per dose. These results were also robust to changes in the underlying assumptions used in the model. The study shows that the TAK-003 vaccination strategy should be tailored to country-specific dengue epidemiology, while taking practicalities into consideration, such as existing NIPs’ calendars, to achieve high coverage. Catch-up programs are shown to further reduce cases, while resulting in even larger cost savings.

## Supporting information

S1 File Model definition. (DOCX)

S2 File Model fitting and parameters. (DOCX)

S3 File Model results. (DOCX)

## Abbreviations

DALY: disability-adjusted life-years
DENV: dengue virus
GDP: gross domestic product
HPV: human papillomavirus
HTA: health technology assessment
NIP: national immunization program
VCD: virologically confirmed dengue
WHO: World Health Organization
WTP: willingness-to-pay
