# Correction: Vaccination strategies, public health impact and cost-effectiveness of dengue vaccine TAK-003: A modeling case study in Thailand

**DOI:** 10.1371/journal.pmed.1004789

**Published:** 2025-10-24

**Authors:** Jing Shen, Elizaveta Kharitonova, Anna Tytula, Justyna Zawieja, Samuel Aballea, Shibadas Biswal, Mayuri Sharma, Supattra Rungmaitree, Rosarin Sruamsiri, Derek Wallace, Riona Hanley

There are errors in the Author Contributions. The correct contributions are:

**Conceptualization:** Jing Shen, Elizaveta Kharitonova

**Data curation:** Elizaveta Kharitonova, Anna Tytula, Justyna Zawieja, Samuel Aballea

**Formal analysis:** Elizaveta Kharitonova, Anna Tytula, Justyna Zawieja, Samuel Aballea

**Methodology:** Jing Shen, Elizaveta Kharitonova, Shibadas Biswal, Mayuri Sharma, Derek Wallace

**Supervision:** Jing Shen

**Validation:** Elizaveta Kharitonova, Anna Tytula, Justyna Zawieja

**Visualization:** Elizaveta Kharitonova, Anna Tytula, Justyna Zawieja

**Writing – original draft:** Elizaveta Kharitonova, Anna Tytula, Justyna Zawieja, Samuel Aballea,

**Writing – review & editing**: Jing Shen, Elizaveta Kharitonova, Anna Tytula, Justyna Zawieja, Samuel Aballea, Shibadas Biswal, Mayuri Sharma, Supattra Rungmaitree, Rosarin Sruamsiri, Derek Wallace, Riona Hanley.
