# Supplementary material for: Vaccination strategies, public health impact and cost-effectiveness of dengue vaccine TAK-003: A modeling case study in Thailand
Source: PLoS Med. 2025 Jun 17;22(6):e1004631. doi: 10.1371/journal.pmed.1004631 (PMC12173404; doi:10.1371/journal.pmed.1004631)
Supplement: S1 File — (DOCX) [file pmed.1004631.s001.docx]

**Assessing the optimal vaccination strategies for dengue vaccine TAK-003, and its public health impact and
cost-effectiveness: A case study in Thailand**

**S1 Material: Model definition**

This supplement (1) describes the structure of the epidemiological model used in the study in detail. There are 2 additional supplements:

- **S2 Material** describes the model fitting and validation process, the model inputs, and the source and rationale for the use of these values.
- **S3 Material** describes additional results not reported in the manuscript.

**Contents**

[1. Modeling of dengue transmission without vaccination 3](#_Toc179974399)

[1.1. Modeling of the host population 3](#_Toc179974400)

[1.1.1. Demographic processes in hosts 3](#_Toc179974401)

[1.1.2. Infection process in hosts 6](#_Toc179974402)

[1.1.3. Infection severity 10](#_Toc179974403)

[1.1.4. Transmissibility of symptomatic and asymptomatic infections 12](#_Toc179974404)

[1.2. Modeling of vector population 13](#_Toc179974405)

[1.3. Serotype distribution 16](#_Toc179974406)

[2. Modeling of vaccination 17](#_Toc179974407)

[2.1. Vaccination strategy 18](#_Toc179974408)

[2.2. Vaccine efficacy and mechanism of action 18](#_Toc179974409)

[2.2.1. Natural boosting of vaccine efficacy and level of vaccine protection 20](#_Toc179974410)

[2.2.2. Impact of vaccination on the acquisition of infection 24](#_Toc179974411)

[2.2.3. Impact of vaccination on infection severity 26](#_Toc179974412)

[2.2.4. Transmissibility of breakthrough infections 28](#_Toc179974413)

[2.2.5. Overview of indirect effects 28](#_Toc179974414)

[3. Estimation of quality-of-life outcomes 30](#_Toc179974415)

[4. Estimation of economic outcomes 31](#_Toc179974416)

[4.1. Direct costs 31](#_Toc179974417)

[4.1.1. Vaccination costs 31](#_Toc179974418)

[4.1.2. Treatment costs 32](#_Toc179974419)

[4.1.2.1. Direct medical costs 32](#_Toc179974420)

[4.1.2.2. Persistent dengue costs 33](#_Toc179974421)

[4.1.2.3. Direct nonmedical costs 33](#_Toc179974422)

[4.2. Indirect costs 33](#_Toc179974423)

[4.2.1. Productivity loss 34](#_Toc179974424)

[4.2.2. School absenteeism 35](#_Toc179974425)

[5. Forward simulations 37](#_Toc179974426)

[6. CHEERS 2022 checklist [23] 38](#_Toc179974427)

[7. References 43](#_Toc179974428)

**List of figures**

[Fig A. Infection process in hosts, for a given serotype. 6](#_Toc178663545)

[Fig B. Dengue infections by severity, in unvaccinated hosts. 10](#_Toc178663546)

[Fig C. Number of vectors per host, by calendar month (example). 13](#_Toc178663547)

[Fig D. Infection process in vectors. 14](#_Toc178663548)

[Fig E. Dengue infections by severity, in vaccinated hosts. 19](#_Toc178663549)

[Fig F. Level of protection against dengue with and without natural boosting of efficacy (illustrative example at an individual level). 20](#_Toc178663550)

[Fig G. Risk of a dengue episode by number of infections before vaccination and now. 23](#_Toc178663551)

[Fig H. Costs included in the model by category and perspective. 31](#_Toc178663552)

# Modeling of dengue transmission without vaccination

This section details the submodel without vaccination, which is used to fit the model to the empirical data and to simulate the epidemiology of dengue in the absence of interventions. The modeling of vaccination is described further in Section 2.

## Modeling of the host population

The host population in the model is divided into compartments based on the hosts’ ages and statuses for each of the 4 dengue serotypes. With 101 age cohorts and 5 statuses possible for each of the 4 dengue serotypes (see Sections 1.1.1 and 1.1.2 for more details), the model without vaccination includes 63,125 compartments (101 × 5 × 5 × 5 × 5).

All the hosts within the same compartment are assumed to be identical. The compartments are mutually exclusive (i.e., each host can only be in 1 compartment at any given point in time) and collectively exhaustive (i.e., all compartments combined include all live hosts at any given point in time).

Each compartment is denoted $H_{i}^{jklm}$, where the index $i$ indicates the hosts’ ages; the indices $j$, $k$, $l$*,* and $m$ indicate their status for DENV (dengue virus)-1, DENV-2, DENV-3, and DENV-4, respectively (see Fig A).

The movements in each compartment can be generalized with the following set of ordinary differential equations:

$$\frac{{dH}_{i}^{jklm}(t)}{dt}= D_{i}^{jklm}(t)+\overset{4}{\underset{s=1}{\sum}}I_{s,i}^{jklm}\left( t \right),$$

where $D_{i}^{jklm}(t)$ describes the movements due to the **demographic processes** (births, aging, and all-cause deaths) in compartment $H_{i}^{jklm}$ at time $t$ (see Section 1.1.1 for more details); $I_{s,i}^{jklm}\left( t \right)$ describes the movements due to the **infection process** for serotype $s$ at time $t$ in the same compartment (see Section 1.1.2 for more details).

In the model with vaccination, the process of vaccination is added to the demographic and infection processes (see Section 2 for more details).

### Demographic processes in hosts

The host population includes the entire population of the area of interest and is stratified into 101 single-year age cohorts from ages 0 to 100 years. The total size and age structure of the population are assumed to remain constant over time. The impact of travelers and migrants (i.e., the inflows into the population other than births and the outflows from the population other than all-cause deaths) is not considered.

In the notation of each compartment $H_{i}^{jklm}$, the index $i$ indicates the hosts’ age cohorts. The examples of the notation are provided in the text box below.

Notation of the hosts’ ages, examples:

- $\boldsymbol{H}_{\boldsymbol{1}}^{\boldsymbol{jklm}}\boldsymbol{-}$ hosts aged 0 years, with the statuses $\boldsymbol{j}$, $\boldsymbol{k}$, $\boldsymbol{l,}$ and $\boldsymbol{m}$ for DENV-1, DENV-2, DENV-3, and DENV-4, respectively.
- $\boldsymbol{H}_{\boldsymbol{2}}^{\boldsymbol{jklm}}$ – hosts aged 1 year, with the statuses $\boldsymbol{j}$, $\boldsymbol{k}$, $\boldsymbol{l,}$ and $\boldsymbol{m}$ for DENV-1, DENV-2, DENV-3, and DENV-4, respectively.
- $\boldsymbol{H}_{\boldsymbol{3}}^{\boldsymbol{jklm}}$ – hosts aged 2 years, with the statuses $\boldsymbol{j}$, $\boldsymbol{k}$, $\boldsymbol{l,}$ and $\boldsymbol{m}$ for DENV-1, DENV-2, DENV-3, and DENV-4, respectively.
- $\boldsymbol{H}_{\boldsymbol{101}}^{\boldsymbol{jklm}}$ – hosts aged 100 years, with the statuses $\boldsymbol{j}$, $\boldsymbol{k}$, $\boldsymbol{l,}$ and $\boldsymbol{m}$ for DENV-1, DENV-2, DENV-3, and DENV-4, respectively.

All movements between the model compartments that are due to demographic processes occur simultaneously after each 1-year period. They include the following 3 elements: births (i.e., newly born susceptible hosts entering compartment $H_{1}^{1111}$); aging (i.e., hosts moving from compartment $H_{i}^{jklm}$ to compartment $H_{i+1}^{jklm}$); and all-cause deaths (i.e., hosts dying during the aging process).

The change in the size of compartment $H_{i}^{jklm}$ due to demographic processes is denoted $D_{i}^{jklm}(t)$. The value of $D_{i}^{jklm}(t)$ is calculated differently for different compartments (see the table below). Because these processes only occur once a year (on the first day of the year), the size of each compartment at time $t$ corresponds to its size as estimated in the last integration of the previous year.

|  | Fully susceptible  ($\boldsymbol{jklm}=1111$) | Not fully susceptible  ($\boldsymbol{jklm}\neq1111$) |
| --- | --- | --- |
| Age 0 years ($\boldsymbol{i}=1$) | Births (inflow)  Aging (outflow) | Aging (outflow) |
| Age ≥1 years ($\boldsymbol{i}\neq1$) | Aging (inflow)  Aging (outflow) | Aging (inflow)  Aging (outflow) |

Thus, for the fully susceptible individuals aged 0 years (compartment $H_{1}^{1111}$), the demographic process is defined by the following equation:

$D_{1}^{1111}(t)=B^{H}-H_{1}^{1111}(t)$,

where $B^{H}$ is the number of new births. For the size and age structure of the host population to remain constant over time, the number of births $B^{H}$ is set to be equal to the size of the cohort aged 0 years ($N_{1}^{H}$).

For the individuals aged 0 years, who are no longer fully susceptible, the demographic process only includes aging out, given by the following equation:

$D_{1}^{jklm\neq1111}\left( t \right)=-H_{1}^{jklm}(t)$.

Finally, for the individuals in other age groups (regardless of their status for each serotype), the demographic process includes both aging in and aging out, given by:

$$D_{i\neq1}^{jklm}(t)=H_{i-1}^{jklm}\left( t \right)\left[ 1-\mu_{i-1} \right]-H_{i}^{jklm}\left( t \right),$$

where $\mu_{i-1}$ is the probability of all-cause death in the age group $i-1$. Thus, the first part of the expression represents the inflow of hosts who survive aging from the cohort aged $i-1$ to the cohort aged $i$; the second part of the expression represents the outflow of hosts who leave the cohort aged $i$*.*

The parameter $\mu_{i}$ is informed by the observed probability of all-cause death at a specific age. The size of the birth cohort is adjusted so that the size and age structure of the population remain constant throughout the simulation (i.e., the number of hosts who enter the simulation each year via birth is equal to the number of hosts who leave the simulation via all-cause deaths). To estimate the size of the birth cohort, the observed probability of all-cause death in each year of age is applied to a hypothetical cohort of 1,000 newborns. This allows simulation of the evolution of the population size in each annual cohort and therefore the relative weight of the birth cohort in the total population. The latter then allows the estimation of the number of newborns required to reach the total population size observed in the specific region or country.

The mortality rates are assumed to be constant over time; thus, the historical and potentially future variations in birth and death rates are not considered. The number of hosts in each age cohort is smaller than the number of individuals in the previous age cohort. The probability of all-cause death in the last age cohort $(\mu_{101})$ is set to 100% to ensure that the entire cohort leaves the simulation after age 100 years.

The impact of dengue-caused deaths averted by vaccination on the total population size and on dengue transmission is assumed to be negligible; therefore, dengue-caused deaths were not explicitly simulated. The probability of all-cause death was assumed to be independent of dengue status or vaccination status. To estimate the benefit of averted dengue-caused deaths on cost and quality of life, the number of dengue-caused deaths with and without vaccination were estimated outside the main transmission model (see Section 1.1.3 for more details).

### Infection process in hosts

The model includes all **4 dengue serotypes**. The infection process for each serotype is identical and can be represented as a sequence of several health states (see Fig A).

In the notation of each model compartment $H_{i}^{jklm}$, the subscripts $j$, $k$, $l,$ and $m$ indicate the hosts’ statuses for specific dengue serotypes. These parameters can take 1 of 5 values (“Susceptible”, “Exposed”, “Infectious”, “Cross-protected”, “Immune”), in line with the infection process assumed for each serotype (see Fig A).

##### Fig A. Infection process in hosts, for a given serotype.


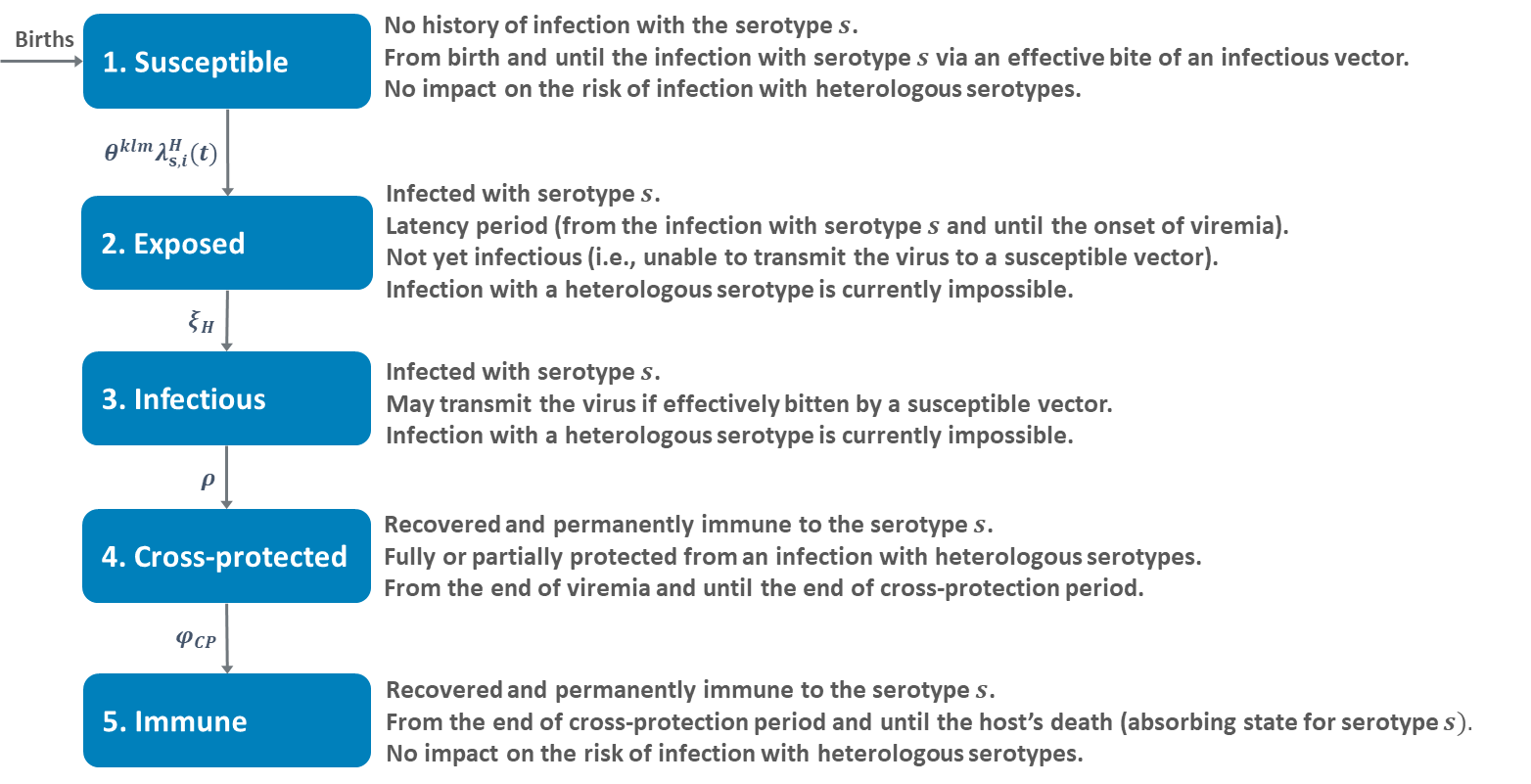


The text box below provides some examples of the notation for the compartments.

Notation of the hosts’ dengue status, examples:

- $\boldsymbol{H}_{\boldsymbol{i}}^{\boldsymbol{1111}}$ – hosts aged $\boldsymbol{i}$ who are susceptible to all 4 serotypes.
- $\boldsymbol{H}_{\boldsymbol{i}}^{\boldsymbol{2111}}$ – hosts aged $\boldsymbol{i}$ who are currently exposed to DENV-1 and susceptible to the other 3 serotypes.
- $\boldsymbol{H}_{\boldsymbol{i}}^{\boldsymbol{5121}}$ – hosts aged $\boldsymbol{i}$ who are immune to DENV-1 and currently exposed to DENV-3.
- $\boldsymbol{H}_{\boldsymbol{i}}^{5555}$– hosts aged $\boldsymbol{i}$ who are immune to all 4 serotypes.

The equations below describe the movements in the compartments due to the infection process with DENV-1. The infection process for the other 3 serotypes can be described in the same way.

$$I_{1,i}^{1klm}\left( t \right)=-\theta^{klm}\lambda_{1,i}^{H}\left( t \right)H_{i}^{1klm}\left( t \right)$$

$$I_{1,i}^{2klm}\left( t \right)=\theta^{klm}\lambda_{1,i}^{H}\left( t \right)H_{i}^{1klm}\left( t \right)-\xi_{H}H_{i}^{2klm}\left( t \right)$$

$$I_{1,i}^{3klm}\left( t \right)=\xi_{H}H_{i}^{2klm}\left( t \right)-\rho H_{i}^{3klm}\left( t \right)$$

$$I_{1,i}^{4klm}\left( t \right)=\zeta_{CP} \left[ \rho H_{i}^{3klm}\left( t \right)-\varphi_{CP}H_{i}^{4klm}\left( t \right) \right]$$

$$I_{1,i}^{5klm}\left( t \right)=\zeta_{CP}\left[ \varphi_{CP}H_{i}^{4klm}\left( t \right) \right]+\left( 1-\zeta_{CP} \right)\left[ \rho H_{i}^{3klm}\left( t \right) \right]$$

The next 5 subsections provide a detailed description of each of the equations above.

**From “Susceptible”**

The state “Susceptible” represents the period of time starting from the hosts’ births (as no maternally acquired immunity was assumed) until the acquisition of the serotype $s$ via an effective bite of a vector infectious of this serotype (which may or may not occur). For the hosts susceptible to DENV-1, the infection process only concerns the acquisition of the infection (i.e., the outflows from the susceptible state). Inflows are determined by births and aging, which are described in Section 1.1.1. The change in the size of the compartment is given by the following equation:

$I_{1,i}^{1klm}\left( t \right)=-\theta^{klm}\lambda_{1,i}^{H}\left( t \right)H_{i}^{1klm}\left( t \right)$,

where $H_{i}^{1klm}\left( t \right)$ is the number of hosts aged $i$ and susceptible to DENV-1 at time $t$ (status for the other 3 serotypes being described by the indices $k$, $l$, and $m$). Parameter $\lambda_{1,i}^{H}(t)$ is the force of infection for DENV-1 at time $t$ for the hosts aged $i$ (more details on the calculation of the force of infection are provided further in the text of this document). Finally, $\theta^{klm}$ is a modifying parameter applied to the force of infection to account for the interactions between the serotypes, including the co-infection with a heterologous serotype (assumed impossible) and full or partial cross-protection.

The parameter $\theta^{klm}$ is defined as follows:

$\theta^{klm}=\min\left( \sigma^{k},\sigma^{l},\sigma^{m} \right)$,

where $\sigma^{k},\sigma^{l},$ and $\sigma^{m}$ are, respectively, the $k$*^th^*, $l$*^th^,* and $m$*^th^* elements of the matrix $\Sigma$, given by:

$$\Sigma=\left[ \begin{aligned} 1 \\ 0 \\ 0 \\ \gamma_{CP} \\ 1 \end{aligned} \right]$$

Simultaneous co-infection with multiple serotypes was assumed impossible, thus $\sigma^{2}$and $\sigma^{3}$ (corresponding to the statuses “Exposed” and “Infectious”) were set to 0. This guarantees that a host will not acquire an infection with DENV-1 while being exposed to or infected with another serotype.

The parameter $\gamma_{CP}$($0\leq\gamma_{CP}\leq1)$ defines the relative risk of acquiring a new infection during **cross-protection** after the infection with another serotype. The model allows for the cross-protection to be perfect (no infection possible during this period) or partial (infection with a heterologous serotype still possible but its risk is reduced). If cross-protection is assumed to be perfect, $\gamma_{CP}$ is equal to 0, which makes infection with DENV-1 impossible at this time.

**Definition of the parameter** $\boldsymbol{\theta}^{\boldsymbol{klm}}$**, examples:**

- $H_{i}^{1111}\left( t \right)$ - a host susceptible to all 4 serotypes; infection with DENV-1 is possible.
  $\theta^{111}$ = min ($\sigma^{1}$ = 1, $\sigma^{1}$ = 1, $\sigma^{1}$= 1) = 1; **force of infection for DENV-1 is not modified.**
- $H_{i}^{1211}\left( t \right)$ - a host currently exposed to DENV-2; co-infection with DENV-1 is impossible.
- $\theta^{211}$ = min ($\sigma^{2}$ = 0, $\sigma^{1}$ = 1, $\sigma^{1}$= 1) = 0; **force of infection for DENV-1 is multiplied by 0.**
- $H_{i}^{1114}\left( t \right)$ - a host currently cross-protected, following an infection with DENV-4; risk of the infection with DENV-1 is either reduced ($\gamma_{CP}>0)$ or equal to 0 ($\gamma_{CP}=0)$.
- $\theta^{114}$ = min ($\sigma^{1}$ = 1, $\sigma^{1}$ = 1, $\sigma^{4}$= $\gamma_{CP}$) = $\gamma_{CP}$; **force of infection for DENV-1 is modified by** $\boldsymbol{\gamma}_{\boldsymbol{CP}}$**.**

**From/to “Exposed”**

The state “Exposed” represents the latency period, i.e., the period following an effective bite of a vector infected with a given serotype until the onset of viremia. Thus, the entries into the state “Exposed” include all hosts newly infected with DENV-1. The exits include the hosts who become infectious after the latency period. The change in the size of the compartment is given by the following equation:

$$I_{1,i}^{2klm}\left( t \right)=\theta^{klm}\lambda_{1,i}^{H}\left( t \right)H_{i}^{1klm}\left( t \right)-\xi_{H}H_{i}^{2klm}\left( t \right),$$

where $\xi_{H}$ is the exit rate calculated as the inverse of the duration of the latency period in hosts.

**From/to “Infectious”**

The state “Infectious” represents the period of viremia. During this period, the infected hosts may transmit the virus if effectively bitten by a susceptible mosquito. The entries into the state “Infectious” include the hosts who become infectious after the latency period. The exits include the hosts who are no longer infectious. The change in the size of the compartment is given by the following equation:

$$I_{1,i}^{3klm}\left( t \right)=\xi_{H}H_{i}^{2klm}\left( t \right)-\rho H_{i}^{3klm}\left( t \right),$$

where $\rho$is the exit rate calculated as the inverse of the duration of viremia.

**From/to “Cross-protected”**

Following the infectious period, the hosts become permanently immune to the infecting serotype. However, before moving to the state “Immune”, they may spend a specified amount of time in the state “Cross-protected”. During this period, they are fully or partially protected from an infection with a heterologous serotype; the impact of this protection on the acquisition of heterologous serotypes is taken into account in the definition of the parameter $\theta^{klm}$ described above.

The entries into the state “Cross-protected” therefore include the hosts who are no longer infectious with DENV-1. The exits include the hosts who are no longer cross-protected. The change in the size of the compartment is given by the following equation:

$$I_{1,i}^{4klm}\left( t \right)=\zeta_{CP} \left[ \rho H_{i}^{3klm}\left( t \right)-\varphi_{CP}H_{i}^{4klm}\left( t \right) \right],$$

where $\zeta_{CP}$ is a binary variable equal to 1 if cross-protection is included and 0 otherwise. Parameter $\varphi_{CP}$ is the exit rate, calculated as the inverse of the duration of cross-protection if cross-protection is included (or equal to 0 otherwise).

**To “Immune”**

The entries into the state “Immune” include the hosts who are no longer cross-protected (if cross-protection is included) or are no longer infectious (if cross-protection is not included). Because each infection results in lifetime immunity to the infecting serotype, the hosts’ statuses for DENV-1 remain unchanged for the rest of their lives. The only exits from the state “Immune” are due to all-cause deaths, which are not considered in the infection process. The change in the size of the compartment is therefore given by the following equation:

$$I_{1,i}^{5klm}\left( t \right)=\zeta_{CP}\left[ \varphi_{CP}H_{i}^{4klm}\left( t \right) \right]+\left( 1-\zeta_{CP} \right)\left[ \rho H_{i}^{3klm}\left( t \right) \right].$$

**Force of infection for hosts**

The force of infection due to serotype $s$ for a host aged $i$ at time $t$ is given by:

$\lambda_{s, i}^{H}\left( t \right)=\frac{\beta^{VH}\beta_{i} b {IV}_{s}(t)}{N^{H}}$,

where ${IV}_{s}\left( t \right)$ is the number of vectors infectious due to serotype $s$ at time $t$, $b$ is the average daily number of bites by an adult female vector, $\beta^{VH}$ is the probability of effective virus transmission from an infectious vector to a susceptible host given a bite, and $\beta_{i}$ is the coefficient used to modify the force of infection for a host aged $i$.

The parameter $N^{H}$ defines the total number of live hosts in the model and is given by the following expression:

$$N^{H}=\overset{101}{\underset{i=1}{\sum}}N_{i}^{H},$$

where $N_{i}^{H}$ is the size of the cohort aged $i$*.*

The parameter $N^{H}$ is not time specific, as the population size is assumed to be constant over time (see Section 1.1.1 for more details on the model population).

### Infection severity

The model did not include specific compartments for the infections of different severity. Instead, the number of new infections was estimated by counting the transitions into the state “Infectious” (see Fig A), and a more granular severity structure was applied afterward (see Fig B). This structure was required to apply the vaccine efficacy considered in the model correctly and to quantify all the elements that contribute to the economic and humanistic burden of dengue.

##### Fig B. Dengue infections by severity, in unvaccinated hosts. The transitions to the elements in blue can be directly affected by vaccination; the transitions to the states in green can only be indirectly affected by vaccination (see Section 2.2); these elements allow estimation of the number of infections with the granularity required for the estimation of economic and quality-of-life burden.


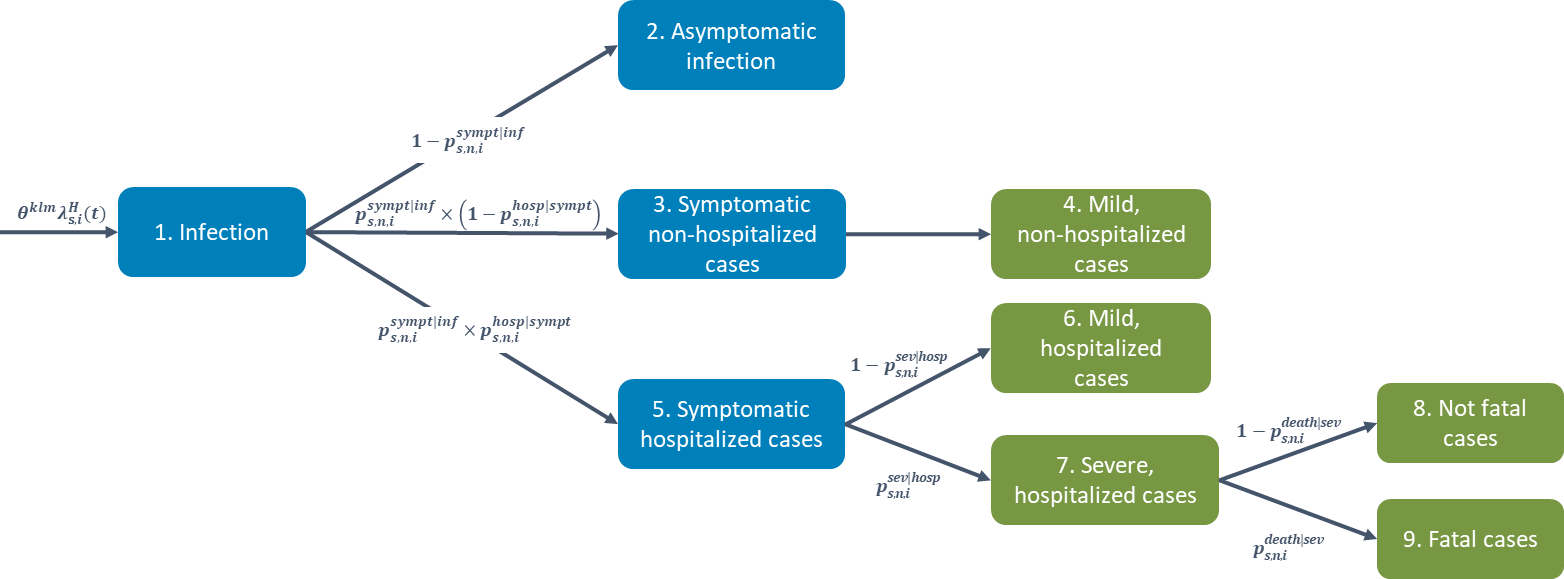


The probability of each outcome in unvaccinated hosts with dengue infection is calculated based on the following input parameters:

- Probability of symptomatic disease with dengue infection $\left( p_{s,n,i}^{sympt|inf} \right)$;
- Probability of hospitalization with a symptomatic case ${(p}_{s,n,i}^{hosp|sympt})$;
- Probability of severe disease with a hospitalized case ${(p}_{s,n,i}^{sev|hosp}$); and
- Probability of dengue death with a severe case ${(p}_{s,n,i}^{death|sev})$,

where the subscript $s$ indicates the infecting serotype, and the subscript $n$ indicates the type of infection (primary, secondary, or postsecondary) to allow simulating that, due to antibody-dependent enhancement, the secondary infections are more likely to result in clinical and severe disease than primary and postsecondary infections. The subscript $i$ indicates the age of the cohort to allow differentiating the probability of different outcomes by age.

Several assumptions were used to inform the final severity distribution:

- Mild cases may or may not be hospitalized;
- All severe cases are hospitalized; thus, all nonhospitalized cases are mild; and
- Dengue-related deaths only occur among severe cases.

The expressions defining the counts of the number of infections of each severity in unvaccinated hosts are provided below.

The number of asymptomatic infections (element #2 in Fig B) with serotype $s$ of type $n$ in the cohort aged $i$ at time $t$ is given by:

$N_{s,n,i}^{asympt}\left( t \right)=N_{s,n,i}^{inf}\left( t \right)\times\left( 1-p_{s,n,i}^{sympt|inf} \right)$.

The total number of symptomatic cases is given by:

$N_{s,n,i}^{sympt}\left( t \right)=N_{s,n,i}^{inf}\left( t \right)\times p_{s,n,i}^{sympt|inf}$.

The number of symptomatic nonhospitalized (mild) cases (elements #3 and #4 in Fig B) is given by:

$N_{s,n,i}^{sympt\_non\_hosp}(t)=N_{s,n,i}^{inf}(t)\times p_{s,n,i}^{sympt|inf}\times\left( 1-p_{s,n,i}^{hosp|sympt} \right)$.

The total number of hospitalized cases (mild or severe, element #5 in Fig B) is given by:

$N_{s,n,i}^{sympt\_hosp}(t)=N_{s,n,i}^{inf}(t)\times p_{s,n,i}^{sympt|inf}\times p_{s,n,i}^{hosp|sympt}$.

The number of mild hospitalized cases (element #6 in Fig B) is given by:

𝑁𝑠,𝑛,𝑖ℎ𝑜𝑠𝑝_𝑚𝑖𝑙𝑑(𝑡)=𝑁𝑠,𝑛,𝑖𝑖𝑛𝑓(𝑡)×𝑝𝑠,𝑛𝑠𝑦𝑚𝑝𝑡|𝑖𝑛𝑓×𝑝𝑠,𝑛,𝑖ℎ𝑜𝑠𝑝|𝑠𝑦𝑚𝑝𝑡×$1-p$𝑠,𝑛,𝑖𝑠𝑒𝑣|ℎ𝑜𝑠𝑝$N_{s,n,i}^{hosp\_mild}(t)=N_{s,n,i}^{inf}(t)\times p_{s,n}^{sympt|inf}\times p_{s,n,i}^{hosp|sympt}\times\left( {1-p}_{s,n,i}^{sev|hosp} \right)$

The number of severe cases (all of which are assumed to be hospitalized; element #7 in Fig B) is given by:

$$N_{s,n,i}^{severe}\left( t \right)=N_{s,n,i}^{inf}\left( t \right)\times p_{s,n}^{sympt|inf}\times p_{s,n,i}^{hosp|sympt}\times p_{s,n,i}^{sev|hosp}.$$

The number of severe nonfatal cases (element #8 in Fig B) is given by:

$N_{s,n,i}^{severe\_non\_fatal}(t)=N_{s,n,i}^{inf}(t)\times p_{s,n,i}^{sympt|inf}\times p_{s,n,i}^{hosp|sympt}\times p_{s,n,i}^{sev|hosp}\times\left( {1-p}_{s,n,i}^{death|sev} \right)$.

Finally, the number of severe fatal cases (dengue-caused deaths; element #9 in Fig B) is given by:

$N_{s,n,i}^{severe\_fatal}(t)=N_{s,n,i}^{inf}(t)\times p_{s,n,i}^{sympt|inf}\times p_{s,n,i}^{hosp|sympt}\times p_{s,n,i}^{sev|hosp}\times p_{s,n,i}^{death|sev}$,

where the parameters $p_{s,n,i}^{sympt|inf}$, $p_{s,n,i}^{hosp|sympt}$, $p_{s,n,i}^{\left( sev | hosp \right)},$ and $p_{s,n,i}^{death|sev}$ were defined above; $N_{s,n,i}^{inf}\left( t \right)$ is the total number of infections with serotype $s$ of type $n$ in the cohort aged $i$ at time $t$, estimated by counting the transitions into the state “Infectious”.

It is also true that:

$$N_{s,n,i}^{inf}\left( t \right)=N_{s,n,i}^{asympt}\left( t \right)+N_{s,n,i}^{sympt}\left( t \right)$$

$$N_{s,n,i}^{sympt}\left( t \right)=N_{s,n,i}^{sympt\_hosp}(t)+N_{s,n,i}^{sympt\_non\_hosp}(t)$$

$$N_{s,n,i}^{severe}\left( t \right)=N_{s,n,i}^{severe\_non\_fatal}(t)+N_{s,n,i}^{severe\_fatal}(t)$$

$$N_{s,n,i}^{sympt\_hosp}\left( t \right)=N_{s,n,i}^{hosp\_mild}\left( t \right)+N_{s,n,i}^{severe}\left( t \right).$$

### Transmissibility of symptomatic and asymptomatic infections

Several studies suggest that symptomatic infections may be associated with higher viral load, which in turn may be associated with greater transmissibility (i.e., a greater chance of virus transmission from an infectious host to a susceptible vector given a bite) [1-3]. The parameter $\psi_{sympt}$ was introduced to define the relative infectiousness of a symptomatic infection relative to an asymptomatic infection. The relative contribution of infectious hosts in each compartment into the force of infection for vectors was estimated based on the probability of having symptomatic or asymptomatic infection in a specific compartment, thus compensating for the lack of explicit compartments in the model for infections with and without symptoms. More details are provided with the definition of the force of infection for vectors in Section 1.2.

## Modeling of vector population

The vector population was modeled explicitly and only included adult female mosquitoes, who transmit the disease from an infected to a susceptible host. Pre-adult stages of vector life cycles were not considered in the present model.

The size of vector population was assumed to be (on average) proportional to the size of the host population, similar to assumptions used in previously published models [4,5]. Because the host population is assumed to be constant (see Section 1.1.1), this implied that the vector population size is also constant (on average throughout the year, without taking into account seasonal variations or the impact of vector control measures).

Seasonality was introduced by varying the **emergence rate** (i.e., the rate at which female adult vectors emerge from nonadult stages). Similar to some previously published models [6,7], the emergence rate was varied using a parametrized sine function with a period of 1 year (see the corresponding equations in the text and an example of a sine curve in Fig C).

##### Fig C. Number of vectors per host, by calendar month (example). Example based on the assumed ratio of 2 adult female vectors per host, amplitude of 0.5, and horizontal shift of −0.5.


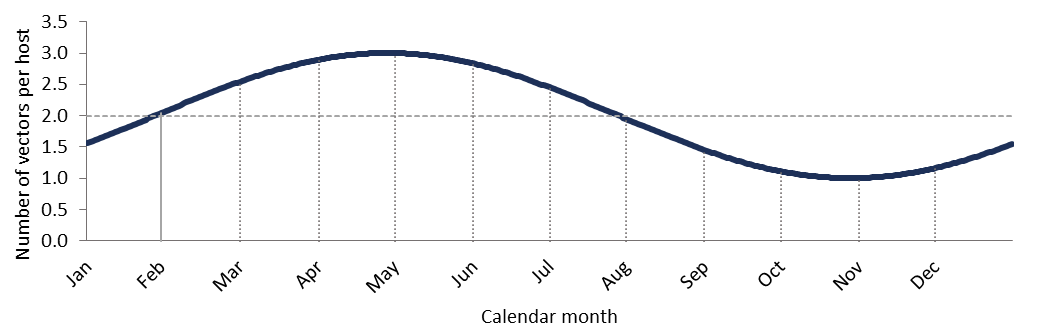


Variations in other parameters that may vary seasonally, such as vector death rates, biting rates and duration of incubation period, were not considered; seasonal variations of a single parameter as a proxy for multiple parameters can be found in previously published dengue models (e.g., Burattini et al [7] varied in mosquito population size only; Lourenço and Recker [6] varied in biting rate only) and proved to be sufficient for reproducing the seasonal patterns of dengue incidence in local adaptations.

The **infection process in the vectors** was modeled following the “Susceptible–Exposed–Infected” structure, with added granularity for the 4 dengue serotypes. The vector population is thus divided into 9 compartments (see Fig D).

##### Fig D. Infection process in vectors. DENV, dengue virus.


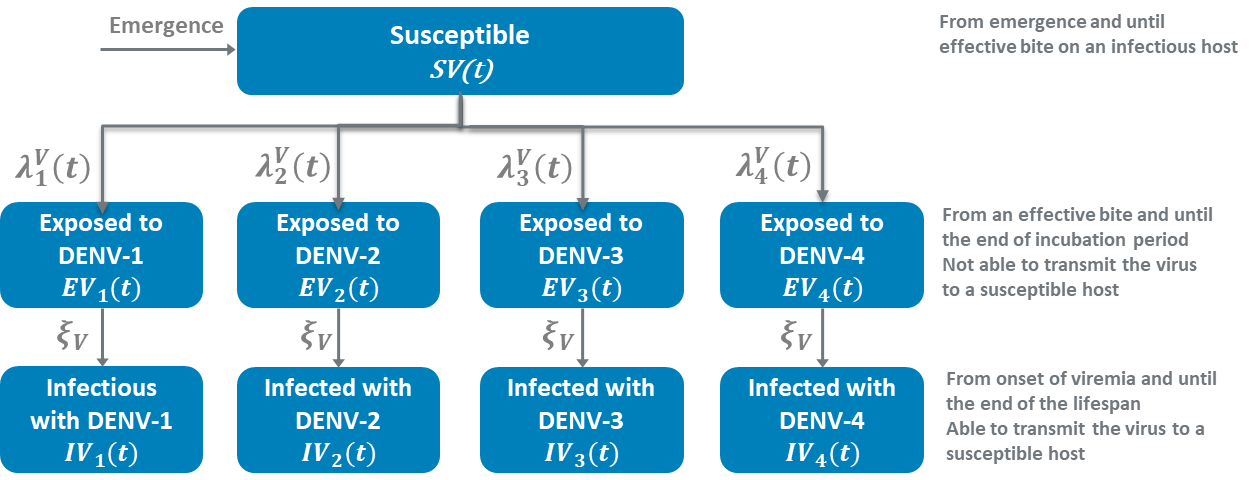


The movements in each compartment are defined by a system of differential equations described below.

**From/to “Susceptible”**

Entries to the state “Susceptible” consist of time-dependent emergence of adult female vectors (all adult vectors were assumed to emerge susceptible, thus assuming no vertical virus transmission in vectors). The exits consist of the vectors who die or acquire an infection with 1 of the dengue serotypes. The movements in the compartment are given by the following expression:

$$\frac{dSV\left( t \right)}{dt}=B_{V}\left( t \right)-\overset{4}{\underset{s=1}{\sum}}\lambda_{s}^{V}\left( t \right)SV\left( t \right)-\mu_{V}SV(t),$$

where $B_{V}\left( t \right)$ is the recruitment rate for female adult vectors at time $t$, $\lambda_{s}^{V}(t)$ is the serotype-specific force of infection for vectors at time $t$ (see definition in the following section), and $\mu_{V}$ is the daily death rate of a vector, calculated as the inverse of the average life span of an adult *Aedes aegypti*.

The emergence of new vectors takes into account the size of the host population so that the average vector population over 1 year remains proportional to the host population (“predator–prey” theory). The average recruitment rate (without seasonality) is therefore defined as:

$\bar{B_{V}}=R_{VH}N^{H}\mu_{V}$,

where $R_{VH}$ is the vector-to-host ratio (i.e., number of adult female vectors per host) and $N^{H}$is the total size of the host population.

To introduce the seasonal variation in the vector population size, a sine function was applied to the average emergence rate. The time-dependent emergence rate is therefore defined as follows:

$B_{V}(t)=\bar{B_{V}}(1+\zeta_{seas}(a_{seas} \sin(\frac{2\Pi}{365} t+b_{seas}))$,

where $\zeta_{seas}$ is a binary variable indicating the presence or absence of seasonality and $a_{seas}$ and $b_{seas}$ are the parameters defining the shape of the sinusoidal function (amplitude and horizontal shift). The model allows fitting the parameters$a_{seas}$ and $b_{seas}$ to empirical data.

**From/to “Exposed”**

The entries to and exits from the exposed state are defined by the following equation:

$\frac{d{EV}^{s}\left( t \right)}{dt}=\lambda_{s}^{V}\left( t \right)SV\left( t \right)-{(\mu}_{V}+\xi_{V}){EV}^{s}(t)$ $s\in\left\{ 1,2,3,4 \right\}$,

where $\xi_{V}$ is the transition rate calculated from the average duration of the latency period in vectors.

**From/to “Infected”**

After the latency period ends, the vectors become infectious with a given serotype. Due to the relatively short lifespan, the recovery in vectors was assumed to be impossible. Thus, they remain infectious until they die (co-infection with multiple serotypes is assumed impossible) and can only carry 1 serotype during their lifespan.

$\frac{d{IV}^{s}(t)}{dt}=\xi_{V}{EV}^{s}\left( t \right)-\mu_{V}{IV}^{s}\left( t \right) s\in\left\{ 1,2,3,4 \right\}$.

**Force of infection**

The example of the force of infection with DENV-1 for vectors is given by:

$\lambda_{1}^{V}\left( t \right)= \frac{{b\beta}^{HV}}{N^{H}}\overset{101}{\underset{i=1}{\sum}}{\underset{k,l,m}{\sum}\Psi_{1,i}^{klm}H}_{i}^{3klm}$,

where $b$ is the average daily number of bites by an adult female vector, $\beta^{HV}$ is the probability of virus transmission from an infectious host to a susceptible vector given a bite, $N^{H}$ is the total size of the host population, and $H_{i}^{3klm}$ is the number of hosts aged $i$ currently infectious with DENV-1. Parameter $\Psi_{1,i}^{klm}$ allows weighing the contribution of the compartment $H_{i}^{3klm}$ into the force of infection based on their chance of having symptomatic or asymptomatic infection (as previously discussed in Section 1.1.4). The presence of symptoms depends on the hosts’ infection history and on the serotype.

The parameter $\Psi_{s,i}^{klm}$ is thus defined as follows:

$\Psi_{s,i}^{klm}=\left\{ \begin{aligned} \left( 1-p_{s,1,i}^{sympt|inf} \right)+\psi_{sympt}p_{s,1,i}^{sympt|inf} if k,l,m contain no \text{“4” or “5” } \\ \left( 1-p_{s,2,i}^{sympt|inf} \right)+\psi_{sympt}p_{s,2,i}^{sympt|inf} if k,l,m contain one “\text{4” }or “\text{5”} \\ \left( 1-p_{s,3,i}^{sympt|inf} \right)+\psi_{sympt}p_{s,3,i}^{sympt|inf} if k,l,m contain more than one “4” or “5” \end{aligned} \right.$,

where $\psi_{sympt}$ is the infectiousness of a symptomatic infection relative to an asymptomatic infection and $p_{s,1,i}^{sympt|inf}$, $p_{s,2,i}^{sympt|inf}$, and $p_{s,3,i}^{sympt|inf}$ define the probability of symptomatic disease with primary, secondary, and postsecondary infection, respectively, with serotype $s$ in hosts aged $i$.

## Serotype distribution

The model allows for up to 4 serotype-specific infections in hosts and only 1 serotype-specific infection in vectors. In hosts, the memory of already-acquired serotypes is preserved (however, the order of past infections with different serotypes is not tracked).

The serotypes are assumed to be identical and have the same probability $\beta^{VH}$ of being transmitted upon a bite by an infectious vector. To desynchronize the occurrence of each serotype, the model is initiated with a single infectious vector introduced into a population of fully susceptible hosts (see “Model fitting” section [Section 1] in S2 Material for more details). However, this single infectious vector is distributed asymmetrically across the 4 serotypes based on arbitrarily specified proportions (e.g., 0.1, 0.2, 0.3, and 0.4 vectors infected with DENV-1, DENV-2, DENV-3, and DENV-4, respectively), which allows for asynchronous peaks in the incidence of each serotype.

In the calibration process, the model is run over a sufficiently long period of time to allow for it to stabilize. The last period simulated in the calibration process (and then used for forward simulations) is characterized by the long-term symmetry of the 4 serotypes; over the period of 20–30 years, the cumulative proportion of each serotype tends to 25%. In shorter time intervals (3–5 years), dominance of a single serotype is often observed. However, the model does not allow specification of any particular target serotype distribution.

# Modeling of vaccination

The model with vaccination is identical to the model without vaccination in terms of the simulation of dengue natural history and basic demographic processes. Modifications were required for the infection process to account for the impact of vaccination on the acquisition, development, and transmission of dengue infection. The process of vaccine administration was added to simulate the transitions of cohorts targeted by a vaccination strategy between unvaccinated and vaccinated states.

Another set of ordinary differential equations was defined for simulations with vaccination. The compartment notation became $H_{i}^{jklmv}$, where the index $v$ indicates the host’s vaccination status. The index $v$ can take on 1 of the following 3 values:

1. Unvaccinated;
2. Vaccinated as seronegative; or
3. Vaccinated as seropositive.

Separate compartments for the hosts vaccinated as seronegative or as seropositive allow preservation of the memory of serostatus at vaccination and differentiation of their level of protection (as discussed further in Section 2.2), in line with the consensus on the role of prior dengue exposure [8,9].

Similar to the model without vaccination, the dynamics in each compartment can be described by the following set of ordinary differential equations:

$$\frac{{dH}_{i}^{jklmv}(t)}{dt}= D_{i}^{jklmv}\left( t \right)+V_{i}^{jklmv}\left( t \right)+\overset{4}{\underset{s=1}{\sum}}I_{s,i}^{jklmv}\left( t \right),$$

where $D_{i}^{jklmv}\left( t \right)$ and $I_{s,i}^{jklmv}\left( t \right)$ are defined the same way as in the model without vaccination (see Section 1.1) and $V_{i}^{jklmv}\left( t \right)$ defines the movements due to the vaccination in the compartment $H_{i}^{jklmv}$ at time $t$.

With 101 age cohorts, 5 statuses possible for each serotype, and 3 vaccination statuses, the total number of compartments in the simulations with vaccination was 189,375 (101 × 5 × 5 × 5 × 5 × 3).

All vaccination-related processes (vaccine administration, evolution of the level of vaccine protection) are modeled as discrete events that occur once a year (on the first day of each simulated year). Here, the time variable $t$ (and the variable $y$ introduced further in the text) refer to discrete 1-year intervals (as in the modeling of the demographic processes described in Section 1.1.1) and not to continuous time (as in the modeling of the infection process).

## Vaccination strategy

The vaccination strategy is defined via a matrix specifying coverage for each age cohort and each year post vaccine introduction. The movements due to vaccination are given by the following equations:

$$V_{i}^{jklm2}\left( t \right)=H_{i}^{11111}(t)\cdot\chi_{i}(t)$$

$$V_{i}^{jklm3}\left( t \right)=H_{i}^{jklm\neq1111 v=1}(t)\cdot\chi_{i}(t)$$

$V_{i}^{jklm1}\left( t \right)=-\left( H_{i}^{11111}(t)+H_{i}^{jklm\neq1111 v=1}(t) \right)\cdot\chi_{i}(t)$,

where $\chi_{i}(t)$ is vaccination coverage for age cohort $i$ at time (year) $t$.

The model does not include separate compartments for individual vaccine doses and does not allow for partial compliance and partial protection between the doses. All vaccinated hosts are assumed to receive the full vaccination schedule and vaccine-derived protection is assumed to start immediately after vaccination.

The exact timing of vaccine introduction may play an important role given the presence of multi-annual cycles in dengue transmission [10]. A sufficiently long period without vaccination is simulated. The vaccination is introduced at a specific year within this period; multiple forward simulations are run, with a varying year of vaccine introduction to account for the uncertainty around the initial condition.

## Vaccine efficacy and mechanism of action

Vaccine efficacy in the model is differentiated by outcome (asymptomatic dengue, symptomatic nonhospitalized dengue, and hospitalized dengue), serotype, and serostatus at vaccination, as well as over time. The model inputs describing the vaccine efficacy are defined as $\eta_{s}^{x,as}\left( y_{v} \right)$, $\eta_{s}^{x,snh}\left( y_{v} \right)$, and $\eta_{s}^{x,sh}\left( y_{v} \right)$ for the efficacy against asymptomatic dengue (index $as$), symptomatic nonhospitalized dengue (index $snh$), and symptomatic hospitalized dengue (index $sh$), respectively. Index $s$ indicates the infecting serotype, index $x$ indicates serostatus at vaccination, and index $y_{v}$ indicates the year since vaccination.

The model also allows the assumption that the level of protection in vaccinated hosts is boosted with each breakthrough infection, in which case the vaccine efficacy is also differentiated by the episode post vaccination (here, the term “episode” refers to a breakthrough infection, i.e., a symptomatic or an asymptomatic infection in a vaccinated individual post vaccination).

The notation of model inputs therefore becomes $\eta_{s,e}^{x,as}\left( y_{b} \right)$, $\eta_{s,e}^{x,snh}\left( y_{b} \right)$, and $\eta_{s,e}^{x,sh}\left( y_{b} \right)$, where the index $e$ indicates the episode post vaccination (with up to 4 episodes possible in individuals who are seronegative at vaccination and up to 3 episodes possible in individuals who are seropositive at vaccination). Index $y_{b}$ indicates the year since last boosting (i.e., breakthrough infection or vaccination itself, the latter being considered as the first boosting). These efficacy parameters can be interpreted as the level of protection against a specific outcome, given an infection with serotype $s$ in an individual who had status $x$ at vaccination, is at risk of the $e$^th^ episode, and was last boosted $y_{b}$ years ago.

The direct impact of vaccination can be summarized with 2 main elements (see Fig E):

- Impact on the acquisition of infection (by modifying the force of infection for vaccinated hosts); and
- Impact on the infection outcomes (by modifying the probability of asymptomatic, symptomatic hospitalized, and symptomatic dengue with breakthrough infections).

##### Fig E. Dengue infections by severity, in vaccinated hosts. The parameters in blue indicate the direct impact of vaccination on acquisition and development of dengue infection.


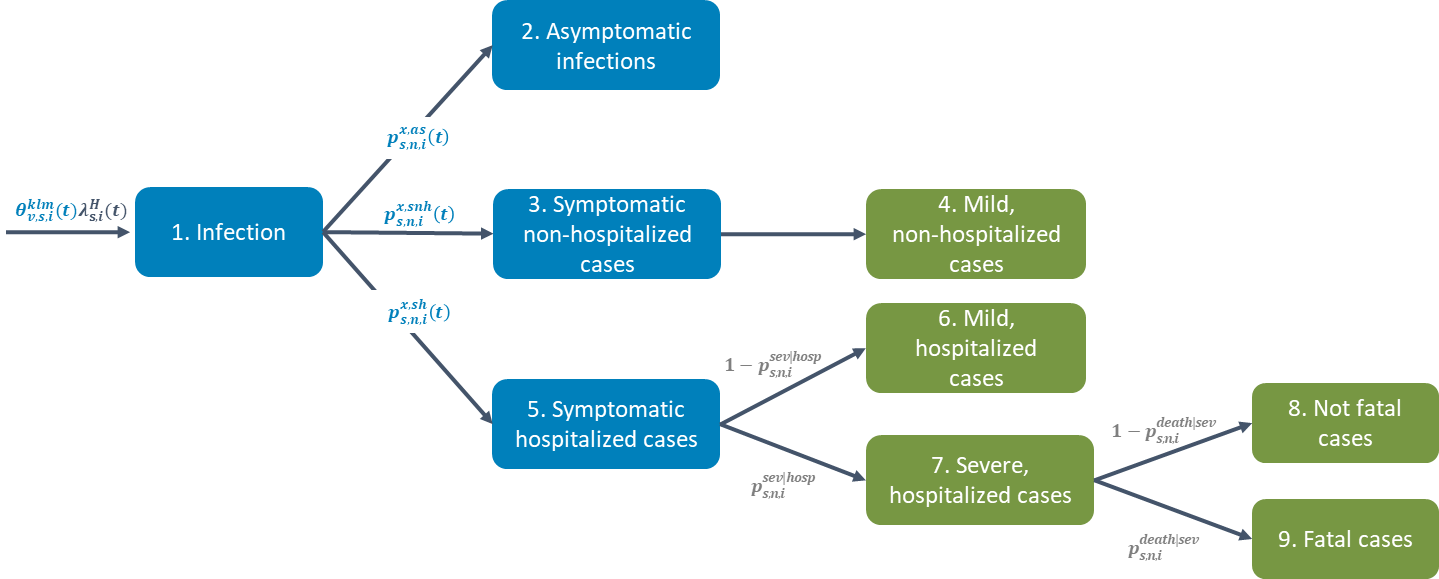


The following subsections are organized as follows: Section 2.2.1 introduces the concept of natural boosting of vaccine efficacy and describes how it is applied in the model, and also defines the parameters that describe the level of vaccine protection (i.e., vaccine efficacy or effectiveness); Section 2.2.2 describes how the infection process in vaccinated hosts is modified to capture the impact of vaccination on the acquisition of infection; Section 2.2.3 describes the definition of infection severity in vaccinated hosts, which takes into account the level of protection against specific outcomes; Section 2.2.4 describes the modeling of a potential reduction in the transmissibility of breakthrough infections; and Section 2.2.5 provides an overview of the mechanisms that drive indirect effects in the presence of vaccination.

### Natural boosting of vaccine efficacy and level of vaccine protection

The model allows for the vaccine-derived protection to be boosted with each breakthrough infection. Epidemiologic and cohort studies have shown that postsecondary infections are generally mild or subclinical [11,12]. Published studies also suggest that exposure to dengue provokes an immune response and increases the level of neutralizing antibody titers, which may be protective against symptomatic disease with the next dengue infection [13,14]. In the model without vaccination, the number of previous infections may affect the probability of symptoms with the next infection, which is usually lower for postsecondary infections than for secondary [15].

The model allows for the inclusion of natural boosting separately for the efficacy against symptomatic (hospitalized or nonhospitalized) and asymptomatic infections.

**In the absence of natural boosting of efficacy**, the level of vaccine protection is defined by how long ago each cohort was vaccinated. At each simulation year, the values for each vaccinated cohort are drawn from the specified efficacy curves $\eta_{s}^{x,as}\left( t \right), \eta_{s}^{x,snh}\left( t \right), \text{and} \eta_{s}^{x,sh}(t)$.

Fig F (below) shows an example of such an efficacy curve on an individual level.

##### Fig F. Level of protection against dengue with and without natural boosting of efficacy (illustrative example at an individual level). This figure is for illustration purposes only. The efficacy curves were selected arbitrarily. In panel A, it can be seen that immediately after vaccination, the individual’s level of protection is defined by the efficacy against the first breakthrough symptomatic infection (referred to as “episode,” with different efficacy used for hospitalized and nonhospitalized infections). Following the first breakthrough infection, the level of protection is defined by the efficacy against the second breakthrough infection (starting from its highest point). The same logic is applied for each infection post vaccination. In this example, the same efficacy curve was used for each infection post vaccination. However, the model allows for the definition of infection-specific efficacy curves. The timing of breakthrough infections was selected arbitrarily for illustration purposes. In the model, it is defined by the background force of infection and is different for each vaccinated cohort. Panel B shows the decrease in efficacy, assuming no boosting of efficacy.


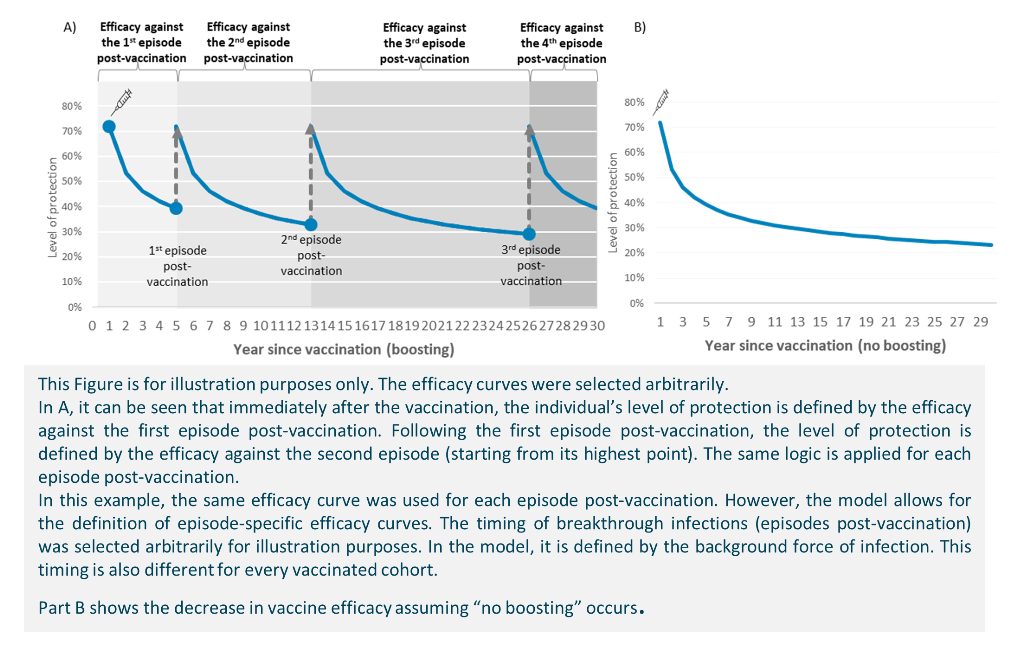


The final level of protection (efficacy) for a host aged $i$, with serotype $s$, at year $y$, by status at vaccination $x$ is given by the following expressions:

$$\overline{\eta}_{s,i}^{x,as}\left( y \right)=\eta_{s}^{x,as}\left( y_{v} \right)$$

$$\overline{\eta}_{s,i}^{x,snh}\left( y \right)=\eta_{s}^{x,snh}\left( y_{v} \right)$$

$\overline{\eta}_{s,i}^{x,sh}\left( y \right)=\eta_{s}^{x,sh}\left( y_{v} \right)$,

where $\eta_{s}^{x,as}\left( t \right)$, $\eta_{s}^{x,snh}\left( t \right)$, and $\eta_{s}^{x,sh}\left( t \right)$ are, respectively, model inputs for efficacy against asymptomatic, symptomatic nonhospitalized, and symptomatic hospitalized infections with serotype $s$, vaccinated at serostatus $x$, at time $t$ since vaccination.

**In the presence of natural boosting of efficacy**, the level of protection no longer depends on the time since vaccination, but instead on the time since last boosting (vaccination being viewed as the first boosting). The efficacy against the first episode post vaccination is applied from the moment of vaccine administration and until the acquisition of the first breakthrough infection e.g., $\eta_{s,1}^{1,as}(y)$ and $\eta_{s,1}^{2,as}(y)$ are the input values of efficacy against asymptomatic dengue caused by serotype $s$ for hosts vaccinated as seronegative and as seropositive, respectively, for whom this would be the first infection post vaccination and who were vaccinated *y* years ago; in this case, the first infection post vaccination coincides with the primary infection for the hosts vaccinated as seronegative; for the hosts vaccinated as seropositive, the first infection post vaccination can be a secondary or a postsecondary infection, depending on how many infections they experienced before vaccination.

Once the host has experienced the first breakthrough infection (i.e., the first episode post vaccination), their level of protection is boosted and is now defined by the input efficacy against the second episode post vaccination (e.g., $\eta_{s,2}^{1,as}(y)$ and $\eta_{s,2}^{2,as}(y)$, which are defined in the same way as above except for the index $y$, which now represents the time since the first episode rather than the time since vaccination). These values of efficacy remain applicable until a new episode occurs. The same logic is applied for each episode post vaccination.

The final level of protection (on an individual level) can be visualized by the curve presented in Fig F, where, following each episode post vaccination, the individual’s level of protection goes up to the initial point of the curve defined for a specific episode post vaccination.

This level of protection can now be referred as real-life effectiveness rather than efficacy, as it combines the “pure” efficacy of the vaccine with the frequency of breakthrough infections, which is driven by the background force of infection.

In the presence of natural boosting of efficacy, the estimation of the level of vaccine protection (effectiveness) has to take into account the number of episodes since vaccination and the time since the last boosting. Fig F illustrates, in a simplified way, the evolution of effectiveness in a specific individual. However, in the model, the effectiveness has to be estimated at the cohort level (separately for each vaccinated cohort and for each simulation year). To keep track of how long ago the hosts in a specific cohort were last boosted, the distribution of each vaccinated cohort was recorded in an object similar to a Markov trace, which tracked the number of hosts in the cohort by:

- The number of infections experienced before vaccination;
- The current number of infections; and
- The number of years since the last infection (or since vaccination).

The first 2 elements indicate which episode post vaccination a specific proportion of the cohort is at risk for (thus indicating which efficacy curve to apply; see Fig G). The last element (similar to “tunnel states” frequently used in Markov models to keep track of how long ago an event occurred) indicates how long ago this proportion of the cohort was last boosted (thus indicating which point of the identified efficacy curve to apply).

##### Fig G. Risk of a dengue episode by number of infections before vaccination and now. Nb, number; seroneg at vac, seronegative at vaccination; seropos at vac, seropositive at vaccination. *No longer at risk of a new infection.


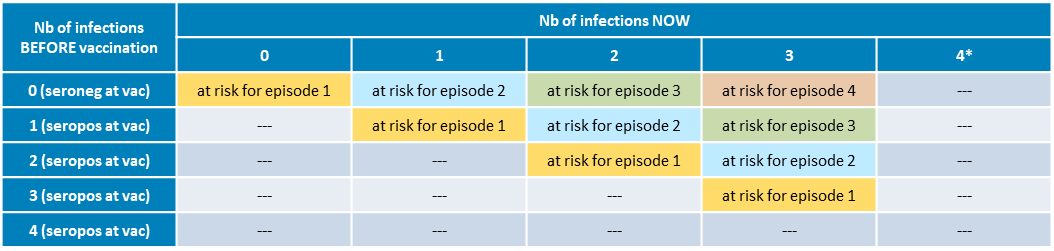


The final effectiveness in each cohort (for each simulation year, each outcome, each serostatus at vaccination, and each serotype) was calculated as a weighted average of the efficacy applicable to different “subgroups” within the cohort (i.e., the proportions of population that are at risk of this episode). The effectiveness for hosts aged $i$, serotype $s$, at time $t$, by status at vaccination and infection severity is given by the following expressions:

$$\overline{\eta}_{s,i}^{1,as}\left( y \right)=\overset{4}{\underset{e=1}{\sum}}\overset{100}{\underset{y=0}{\sum}}T_{i,e}^{1}(y,y_{b})\cdot\eta_{s,e}^{s,as}(y_{b})$$

$$\overline{\eta}_{s,i}^{1,sh}\left( y \right)=\overset{4}{\underset{e=1}{\sum}}\overset{100}{\underset{y=0}{\sum}}T_{i,e}^{1}(y,y_{b})\cdot\eta_{s,e,y}^{1,sh}(y_{b})$$

$$\overline{\eta}_{s,i}^{1,snh}\left( y \right)=\overset{4}{\underset{e=1}{\sum}}\overset{100}{\underset{y=0}{\sum}}T_{i,e}^{1}(y,y_{b})\cdot\eta_{s,e,y}^{1,snh}(y_{b})$$

$$\overline{\eta}_{s,i}^{2,as}\left( y \right)=\overset{3}{\underset{e_{v}=1}{\sum}}\overset{4}{\underset{e=1}{\sum}}\overset{100}{\underset{y=0}{\sum}}T_{{i,e}_{v},e}^{2}\left( y,y_{b} \right)\cdot\eta_{s,e-e_{v}+1}^{2,as}(y_{b})$$

$$\overline{\eta}_{s,i}^{2,sh}\left( y \right)=\overset{3}{\underset{e_{v}=1}{\sum}}\overset{4}{\underset{e=1}{\sum}}\overset{100}{\underset{y=0}{\sum}}T_{{i,e}_{v},e}^{2}(y,y_{b})\cdot\eta_{s,e-e_{v}+1}^{2,sh}(y_{b})$$

$$\overline{\eta}_{s,i}^{2,snh}\left( y \right)=\overset{3}{\underset{e_{v}=1}{\sum}}\overset{4}{\underset{e=1}{\sum}}\overset{100}{\underset{y=0}{\sum}}T_{{i,e}_{v},e}^{2}(y,y_{b})\cdot\eta_{s,e-e_{v}+1}^{2,snh}(y_{b}),$$

where $T_{i,e}^{1}(y,y_{b})$ is the proportion of individuals aged *i* with the number of episodes post vaccination equal to *e−1* and years since the last episode equal to $y_{b}$ in the entire vaccinated population aged *i*, who were seronegative at vaccination, at simulation year *y*.

The parameters $\eta_{s,e}^{1,as}(y_{b})$, $\eta_{s,e}^{1,snh}(y_{b})$, and $\eta_{s,e}^{1,sh}(y_{b})$ were defined above.

The parameter $T_{{i,e}_{v},e}^{2}\left( y,y_{b} \right)$is the proportion of individuals aged $i$ with the number of episodes post vaccination equal to *e−1*, who were vaccinated after $e_{v}$ infections and years since the last episode equal to $y_{b}$ in the entire vaccinated population aged $i$, who were seropositive at vaccination, at simulation year *y*.

### Impact of vaccination on the acquisition of infection

Equations defining the infection process with DENV-1 in the presence of vaccination are presented below. The infection process with other serotypes can be described in the same way.

$$I_{1,i}^{1klmv}\left( t \right)=-\theta_{v,1,i}^{klm}{\left( t \right)\lambda}_{1,i}^{H}\left( t \right)H_{i}^{1klmv}\left( t \right)$$

$$I_{1,i}^{2klmv}\left( t \right)=\theta_{v,1,i}^{klm}\lambda_{1,i}^{H}\left( t \right)H_{i}^{1klmv}\left( t \right)-\xi_{H}H_{i}^{2klmv}\left( t \right)$$

$$I_{1,i}^{3klmv}\left( t \right)=\xi_{H}H_{i}^{2klmv}\left( t \right)-\rho H_{i}^{3klmv}\left( t \right)$$

$$I_{1,i}^{4klmv}\left( t \right)=\zeta_{CP} \left[ \rho H_{i}^{3klmv}\left( t \right)-\varphi_{CP}H_{i}^{4klmv}\left( t \right) \right]$$

$$I_{1,i}^{5klmv}\left( t \right)=\zeta_{CP}\left[ \varphi_{CP}H_{i}^{4klmv}\left( t \right) \right]+\left( 1-\zeta_{CP} \right)\left[ \rho H_{i}^{3klmv}\left( t \right) \right]$$

These equations are identical to those in the model without vaccination (see Section 1.1.2), with 2 exceptions. First, the index $v$ is added to the notation of each compartment to reflect the host’s vaccination status. Second, the modifying parameter applied to the force of infection for DENV-1 is now labeled $\theta_{v,1,i}^{klm}(t)$ (previously $\theta^{klm}$) and is given by:

$\theta_{v,s,i}^{klm}(t)=\min\left( \sigma_{k}^{v,s,i},\sigma_{l}^{v,s,i},\sigma_{m}^{v,s,i} \right)$,

where $\sigma_{k}^{v,s,i}$, $\sigma_{l}^{v,s,i}$, and $\sigma_{m}^{v,s,i}$ are, respectively, the $k$*^th^*, $l$*^th^*, and $m$*^th^* elements of the matrixes $\Sigma_{vac}^{v,s,i}$, given by:

$\Sigma_{vac}^{1,s,i}=\left[ \begin{aligned} 1 \\ 0 \\ 0 \\ \gamma_{CP} \\ 1 \end{aligned} \right]$ $\Sigma_{vac}^{2,s,i}(t)=\left[ \begin{aligned} 1-\overline{\eta}_{s,i}^{1,inf}(y) \\ 0 \\ 0 \\ \gamma_{CP} \\ 1-\overline{\eta}_{s,i}^{1,inf}(y) \end{aligned} \right]$ $\Sigma_{vac}^{3,s,i}(t)=\left[ \begin{aligned} 1-\overline{\eta}_{s,i}^{2,inf}(y) \\ 0 \\ 0 \\ \gamma_{CP} \\ 1-\overline{\eta}_{s,i}^{2,inf}(y) \end{aligned} \right]$,

where the parameters $\overline{\eta}_{s,i}^{1,inf}(y)$ and $\overline{\eta}_{s,i}^{2,inf}(y)$ represent the level of vaccine protection against infection with serotype $s$ in simulation year $y$ in the hosts aged $i$ vaccinated as seronegative and seropositive, respectively.

The first matrix $\Sigma_{vac}^{1,s,i}$ for unvaccinated hosts was defined and described in Section 1.1.2. The matrixes for vaccinated hosts are defined similarly, except for the first and last elements of the matrix, which are modified with parameter $\overline{\eta}_{s,i}^{x,inf}\left( y \right)$ defined above.

The level of protection against infection with serotype $s$ in vaccine recipients aged $i$ with the serostatus at vaccination $x$ is given by the following expression:

$$\overline{\eta}_{s,i}^{x,inf}\left( y \right) =\overline{\eta}_{s,i}^{x,as}\left( y \right)\times(\overline{p}_{s,i}^{x, as}\left( y \right) +$$

$$\overline{p}_{s,i}^{x,snh}\left( y \right)\times\overline{\eta}_{s,i}^{x,snh}\left( y \right) \times\delta+$$

$$\overline{p}_{s,i}^{x,sh} \left( y \right)\times\overline{\eta}_{s,i}^{x,sh} \left( y \right) \times\delta)+$$

$$\overline{\eta}_{s,i}^{x,snh}\left( y \right) \times\overline{p}_{s,i}^{x,snh}\left( y \right) \times\left( 1-\delta\right)+$$

$\overline{\eta}_{s,i}^{x,sh} \left( y \right) \times\overline{p}_{s,i}^{x,sh} \left( y \right) \times\left( 1-\delta\right)$,

where $\overline{\eta}_{s,i}^{x,as}(y)$, $\overline{\eta}_{s,i}^{x,snh}(y)$, and $\overline{\eta}_{s,i}^{x,sh}(y)$ are, respectively, the efficacy or effectiveness against asymptomatic, symptomatic nonhospitalized, and symptomatic hospitalized dengue infection with serotype $s$ in the fraction of the cohort aged $i$ who had serostatus $x$ at vaccination. Further details on the definition of these parameters were provided in the previous section.

The parameters $\overline{p}_{s,i}^{x, as}(y)$, $\overline{p}_{s,i}^{x,snh}(y)$, and $\overline{p}_{s,i}^{x,sh}(y)$ represent the average weighted probability of each outcome of the infection with serotype $s$ in the fraction of the cohort aged $i$ who had serostatus $x$ at vaccination.

The binary parameter $\delta$ defines whether the symptomatic infections averted with vaccination become asymptomatic instead ($\delta=1$) or are prevented entirely ($\delta=0$). If symptomatic infections averted with vaccination are assumed to become asymptomatic (i.e., $\delta=1$), the expression above becomes:

$$\overline{\eta}_{s,i}^{x,inf}\left( y \right) =\overline{\eta}_{s,i}^{x,as}\left( y \right)\times(\overline{p}_{s,i}^{x, as} \left( y \right) +$$

$$\overline{p}_{s,i}^{x,snh}\left( y \right)\times\overline{\eta}_{s,i}^{x,snh}\left( y \right)+$$

$\overline{p}_{s,i}^{x,sh} \left( y \right)\times\overline{\eta}_{s,i}^{x,sh}\left( y \right) )$,

where the expression in the brackets defines the proportion of infections that are asymptomatic in vaccinated individuals (including the symptomatic infections that become asymptomatic following vaccination). The level of protection against overall infection is therefore defined by this new proportion of asymptomatic infection and the vaccine efficacy or effectiveness against them.

If symptomatic infections are assumed to be prevented entirely with vaccination (i.e., $\delta=0$), the original expression becomes:

$$\bar{\eta}_{s,i}^{x}\left( y \right) =\overline{\eta}_{s,i}^{x,as} \left( y \right)\times\overline{p}_{s,i}^{x, as} \left( y \right) +$$

$$\overline{\eta}_{s,i}^{x,snh}\left( y \right)\times\overline{p}_{s,i}^{x,snh}\left( y \right)+$$

$\overline{\eta}_{s,i}^{x,sh} \left( y \right)\times\overline{p}_{s,i}^{x,sh} \left( y \right)$.

The level of protection against infection is therefore a weighted average of the level of protection against each individual outcome.

As can be seen from the equations above, the level of protection against infection is nonzero if ≥1 of the 2 following conditions is met: efficacy (effectiveness) against asymptomatic dengue is positive (i.e., $\overline{\eta}_{s,i}^{x,as}>0)$, and/or the symptomatic infections averted with vaccination are eliminated entirely and do not become asymptomatic instead (i.e., $\delta=0$).

### Impact of vaccination on infection severity

The distribution of infections in vaccinated individuals by severity was modified to account for the impact of vaccination. Specifically, the proportions of symptomatic nonhospitalized, symptomatic hospitalized, and asymptomatic infections were re-estimated for vaccinated individuals at each simulation year, taking into account the current values of vaccine efficacy or effectiveness.

The proportion $p_{s,n,i}^{x,snh}\left( t \right)$ of symptomatic nonhospitalized cases with serotype $s$ of type $n$ in vaccinated hosts aged $i$ with serostatus $x$ at vaccination is given by:

$$p_{s,n,i}^{x,snh}\left( t \right)=\frac{1}{1-\overline{\eta}_{s,i}^{x}\left( y \right)} \times p_{s,n,i}^{sympt|inf}\times\left( {1-p}_{s,n,i}^{hosp|sympt} \right)\times\left( 1-\overline{\eta}_{s,i}^{x,snh}\left( y \right) \right),$$

where $\overline{\eta}_{s,i}^{x}\left( y \right)$ is the efficacy (or effectiveness) against infection, $p_{s,n,i}^{sympt|inf}$ and $p_{s,n,i}^{hosp|sympt}$ are the probability of symptomatic disease with dengue infection and the probability of hospitalization with symptomatic disease in unvaccinated individuals (see Section 1.1.3), and $\overline{\eta}_{s,i}^{x,snh}\left( y \right)$ is the efficacy (or effectiveness) against nonhospitalized dengue.

Similarly, the proportion $p_{s,n,i}^{x,sh}\left( t \right)$ of symptomatic hospitalized cases in the same vaccinated hosts can be given by:

$$p_{s,n,i}^{x,sh}\left( t \right)=\frac{1}{1-\overline{\eta}_{s,i}^{x}\left( y \right)} \times p_{s,n,i}^{sympt|inf}{\times p}_{s,n,i}^{hosp|sympt}\times\left( 1-\overline{\eta}_{s,i}^{x,sh}\left( y \right) \right),$$

where $\overline{\eta}_{s,i}^{x,sh}\left( y \right)$ is the efficacy (or effectiveness) against hospitalized dengue.

The proportion of symptomatic cases in the same vaccinated host can be given by:

$p_{s,n,i}^{x,sympt}\left( t \right)=p_{s,n,i}^{x,snh}\left( t \right)+p_{s,n,i}^{x,sh}\left( t \right)$.

Finally, the proportion of asymptomatic infections in the same vaccinated hosts is given by:

$p_{s,n,i}^{x,as}\left( t \right)=1-p_{s,n,i}^{x,snh}\left( t \right)-p_{s,n,i}^{x,sh}\left( t \right)$.

Probabilities of other outcomes (mild, severe, fatal dengue) are not directly affected by vaccination.

The expressions defining the counts of the number of infections of each severity in vaccinated hosts are provided below.

The object $N_{s,n,i}^{v,inf}\left( t \right)$ is the number of all infections of serotype $s$, infection type $n$ in individuals aged$i$ with vaccination status $v$, at time $t$. For vaccination status 1 (unvaccinated hosts), equations were defined in Section 1.1.3.

The number of asymptomatic infections (element #2 in Fig E) with serotype $s$ of type $n$ in the cohort aged $i$ at time $t$ is given by:

$N_{s,n,i}^{x+1,asympt}\left( t \right)=N_{s,n,i}^{x+1,inf}\left( t \right)\times p_{s,n,i}^{x,as}\left( t \right)$.

The number of symptomatic nonhospitalized (mild) cases (elements #3 and #4 in Fig E) is given by:

$N_{s,n,i}^{x+1,sympt\_non\_hosp}(t)=N_{s,n,i}^{x+1,inf}(t)\times p_{s,n,i}^{x,snh}\left( t \right)$.

The total number of hospitalized cases (mild or severe, element #5 in Fig E) is given by:

$N_{s,n,i}^{x+1,sympt\_hosp}(t)=N_{s,n,i}^{x+1,inf}(t)\times p_{s,n,i}^{x,sh}\left( t \right)$.

The number of mild hospitalized cases (element #6 in Fig E) is given by:

$N_{s,n,i}^{x+1,hosp\_mild}(t)=N_{s,n,i}^{x+1,inf}(t)\times p_{s,n,i}^{x,sh}\left( t \right)\times\left( {1-p}_{s,n,i}^{sev|hosp} \right)$.

The number of severe cases (all of which are assumed to be hospitalized, element #7 in Fig E) is given by:

${N,}_{s,n,i}^{x+1,severe}(t)=N_{s,n,i}^{x+1,inf}(t)\times p_{s,n,i}^{x,sh}\left( t \right)\times p_{s,n,i}^{sev|hosp}$.

The number of severe nonfatal cases (element #8 in Fig E) is given by:

$N_{s,n,i}^{x+1,severe\_non\_fatal}(t)=N_{s,n,i}^{x+1,inf}(t)\times p_{s,n,i}^{x,sh}\left( t \right)\times p_{s,n,i}^{sev|hosp}\times\left( {1-p}_{s,n,i}^{death|sev} \right)$.

Finally, the number of severe fatal cases (dengue-caused deaths, element #9 in Fig E) is given by:

$N_{s,n,i}^{x+1,severe\_fatal}(t)=N_{s,n,i}^{x+1,inf}(t)\times p_{s,n,i}^{x,sh}\left( t \right)\times p_{s,n,i}^{sev|hosp}\times p_{s,n,i}^{death|sev}$,

Where the parameters $p_{s,n,i}^{x,as}\left( t \right)$, $p_{s,n,i}^{x,snh}\left( t \right)$, and $p_{s,n,i}^{x,sh}\left( t \right)$ were defined above; $p_{s,n}^{sev|hosp}$ is the probability of severe disease with hospitalization, $p_{s,n,i}^{death|sev}$ is the probability of dengue death with severe disease, and $N_{s,n,i}^{x+1,inf}\left( t \right)$ is the total number of infections with serotype $s$ of type $n$ in the cohort aged $i$ with vaccination status $x+1$ (2 for hosts vaccinated as seronegative and 3 for hosts vaccinated as seropositive), at time $t$, estimated by counting the transitions to the state “Infectious.”

### Transmissibility of breakthrough infections

The model allows the assumption of different transmissibility of infections in vaccinated hosts (relative to the similar infections in unvaccinated hosts). This level can be specified separately for symptomatic and asymptomatic infections ($\psi_{asympt}^{vac},\psi_{sympt}^{vac}$).

This, in turn, affects the calculation of the vector force of infection, which is now given by the following expression (example for DENV-1; force of infection for other serotypes may be defined in the same way):

$\lambda_{1}^{V}\left( t \right)= \frac{{b(t)\beta}^{H\to V}}{N^{H}}\underset{k,l,m,v}{\sum}\sum_{i=1}^{101}{\Psi_{1,i}^{klmv}\left( t \right)H}_{i}^{3klmv}$.

The parameter $\Psi_{1,i}^{klmv}$ now represents relative infectiousness of the individuals in each compartment $H_{i}^{3klmv}$, as determined by the presence of symptoms, as well as vaccination status.

The parameter $\Psi_{s,i}^{klmv}$ for $v\neq1$ is defined as follows:

$\Psi_{s,i}^{klmv}\left( t \right)=\left\{ \begin{aligned} p_{s,1,i}^{v-1,as}\left( t \right)\psi_{asympt}^{vac} +\psi_{sympt}^{vac}p_{s,1,i}^{v-1,sympt}\left( t \right) if k,l,m contain no \text{4}or \text{5} \\ p_{s,2,i}^{v-1,as}\left( t \right)\psi_{asympt}^{vac}+\psi_{sympt}^{vac}p_{s,2,i}^{v-1,sympt}\left( t \right) if k,l,m contain one \text{4}or \text{5} \\ p_{s,3,i}^{v-1,as}\left( t \right)\psi_{asympt}^{vac}+\psi_{sympt}^{vac}p_{s,3,i}^{v-1,sympt}\left( t \right) if k,l,m contain more than one "4" or "5" \end{aligned} \right.$,

where $\psi_{sympt}^{vac}$ and $\psi_{asympt}^{vac}$ define the infectiousness of a symptomatic and an asymptomatic infection in a vaccinated host (relative to an asymptomatic infection in an unvaccinated host), $p_{s,1,i}^{v-1,as}\left( t \right)$, $p_{s,2,i}^{v-1,as}\left( t \right)$, and $p_{s,3,i}^{v-1,as}\left( t \right)$ are, respectively, the proportion of asymptomatic infections in primary, secondary, and postsecondary infections with serotype $s$ in hosts aged $i$, with vaccination status $v$, and $p_{s,1,i}^{v-1,sympt}\left( t \right)$, $p_{s,2,i}^{v-1,sympt}\left( t \right)$, and $p_{s,3,i}^{v-1,sympt}\left( t \right)$ are, respectively, the proportion of symptomatic cases in primary, secondary, and postsecondary infections.

### Overview of indirect effects

In the present model, the indirect effects of vaccination that benefit both vaccinated and unvaccinated hosts through a reduction of the overall circulation of dengue virus may arise via 3 mechanisms.

First, if the efficacy against dengue infection is nonzero, the overall number of dengue infections (and therefore the force of infection) is reduced. The conditions under which the efficacy against infection is nonzero are discussed in Section 2.2.2.

Second, the probability of symptomatic disease in vaccinated individuals may be lower than in unvaccinated individuals if the vaccine efficacy is nonzero (see Section 2.2.3). If symptomatic infections are assumed to be more transmissible than asymptomatic infections (see Section 1.1.4), the contribution of vaccinated individuals to the force of infection is reduced.

Finally, a potentially lower transmissibility of breakthrough infections may also lead to lower virus circulation in the population (see Section 2.2.4).

# Estimation of quality-of-life outcomes

Disability-adjusted life years (DALYs) are commonly used to quantify the impact of dengue (or other infectious diseases) on quality of life [16-18]. This measure combines years lived with disability (YLDs) due to being in poor health and years of life lost (YLLs) due to premature death. The total number of DALYs can thus be given by:

$DALYs=YLDs+YLLs.$

The formulas below were used to estimate YLDs and YLLs and were originally taken from the publication by Devleesschauwer et al. [19]:

$$YLDs=N\times DW\times\int_{A}^{A+L}\left\{ KCxe^{-\beta x}e^{-r\left( x-a \right)}+(1-K)e^{-r\left( x-a \right)} \right\}dx$$

$$YLLs=M\times\int_{A}^{A+L}\{KCxe^{-\beta x}e^{-r\left( x-a \right)}+(1-K)e^{-r\left( x-a \right)}\}dx$$

where $N$ is the number of cases, $M$ is the number of deaths, $DW$ is the disability weight equal to 0 for perfect health and 1 for death, $K$ is a modulating factor equal to 1 if age weighting is applied and 0 otherwise, $A$ is the age at disease onset or at death, $L$ is the duration of the disease or the remaining life expectancy at the age of death, $C$ and $\beta$ are the constants used in a standard age-weighting formula, $r$ is the discount rate, $x$ is the concerned age, and $a$ is the age to which the burden is assigned.^[[1]](#footnote-1)^

The age weighting mentioned above implies that the “value” of life depends on age, with greater weight being attributed to the YLLs in age groups viewed as more important, from a societal perspective. The study by Devleesschauwer et al. [19] cited the age from 9 to 56 years as “more important,” referring to an earlier study by Murray [20].

Because the model population was stratified by a single year of age, the age $A$ of disease onset or death was set to the middle of the year. The discounting rate $r$ was equal to the display discount rate for outcomes; the number of DALYs was therefore re-estimated every time the display rate changed.

The number of YLDs was estimated separately for 3 types of dengue cases: mild, severe, and persistent, based on the outcome-specific disability durations and weights. The number of YLLs included all the YLLs, including those that went beyond the simulation timeframe.

The estimation of DALYs in the model R codes was based on the sample code provided in the supplementary materials of the publication by Devleesschauwer et al. [19].

# Estimation of economic outcomes

The model includes direct costs (cost of vaccination, cost of dengue treatment) and indirect costs (productivity lost, cost of school absenteeism). The costs are estimated based on the number of cases of each severity predicted for a specific simulation year (see Section 1.1.3 for more details) and discounted using the following formula:

$d\left( y \right)=\frac{1}{\left( 1+r \right)^{y-1}}$,

where $d(y)$ is the discount factor for the costs occurring in year $y$ and $r$is the annual discount rate.

Two perspectives (payer and societal) are used in the estimation. The payer perspective includes the cost of vaccination, the direct medical cost, and the cost of persistent dengue. The societal perspective includes all the costs included in the payer perspective plus the direct nonmedical costs and indirect costs (see Fig H).

##### Fig H. Costs included in the model by category and perspective.


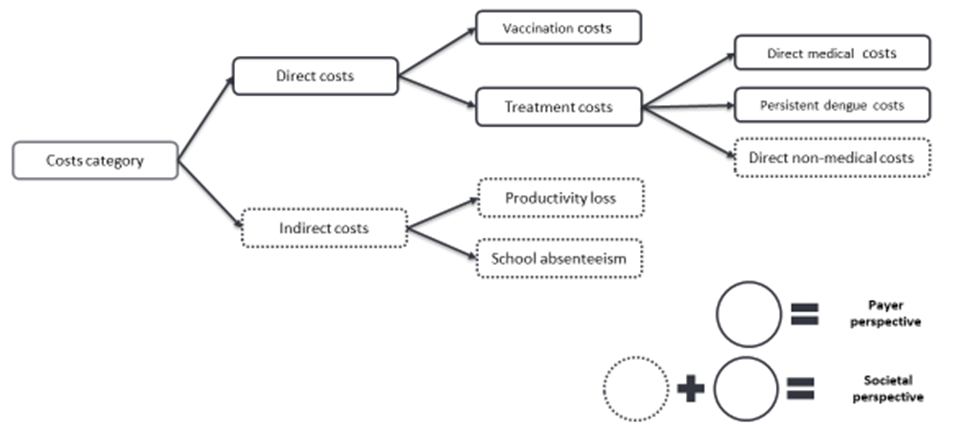


## Direct costs

### Vaccination costs

The cost of vaccination includes the cost of vaccine and the cost of vaccine administration. The latter is differentiated for routine vaccination and catch-up vaccination, as it may depend on the age of the cohort being vaccinated (e.g., because of school-based vaccination or co-administration with other vaccines).

To estimate the total cost of a vaccination strategy, the individual unit costs were multiplied by the number of dispensed doses, predicted for the strategy of interest (and based on the coverage and size of the cohort[s] being vaccinated).

The cost of vaccination of a cohort aged $i$ at time $t$ can be given by the following formula:

$C_{vac}\left( i,t \right)=\left( {V_{i}^{jklm2}\left( t \right)+V}_{i}^{jklm3}\left( t \right) \right)\cdot n_{vac}\cdot\left( c_{vac}+c_{vac\_adm} \right)$,

where $V_{i}^{jklm2}\left( t \right)$and $V_{i}^{jklm3}\left( t \right)$ are the number of newly vaccinated hosts (as defined by the movements from the status “Unvaccinated” to the status “Vaccinated”; see definitions in Section 2.1), $n_{vac}$ is the number of vaccine doses in the full vaccination schedule, $c_{vac}$ is the cost of 1 vaccine dose, and $c_{vac\_adm}$ is the cost of administration of 1 vaccine dose.

### Treatment costs

#### Direct medical costs

Direct medical costs include the cost of ambulatory visits, hospital stays, medications, tests, etc. Depending on the healthcare system and perspective, these costs may, therefore, represent all items covered by the payer.

The input unit costs can be differentiated by case severity (nonhospitalized, hospitalized mild, and hospitalized severe) and by age. The direct medical cost for hosts aged$i$, at time $t$, is given by the following formula:

$$C_{dir\_med}\left( i,t \right)=c_{dir\_med}^{sympt\_non\_hosp}\left( i \right)\cdot\overset{3}{\underset{n=1}{\sum}}\overset{4}{\underset{s=1}{\sum}}\overset{3}{\underset{v=1}{\sum}}N_{s,n,i}^{v,sympt\_non\_hosp}\left( t \right)+$$

$$c_{dir\_med}^{hosp\_mild}\left( i \right)\cdot\overset{3}{\underset{n=1}{\sum}}\overset{4}{\underset{s=1}{\sum}}\overset{3}{\underset{v=1}{\sum}}N_{s,n,i}^{v,hosp\_mild}\left( t \right)+$$

$$c_{dir\_med}^{hosp\_severe}\left( i \right)\cdot\overset{3}{\underset{n=1}{\sum}}\overset{4}{\underset{s=1}{\sum}}\overset{3}{\underset{v=1}{\sum}}N_{s,n,i}^{v,hosp\_severe}\left( t \right),$$

where $c_{dir\_med}^{sympt\_non\_hosp}\left( i \right)$, $c_{dir\_med}^{hosp\_mild}\left( i \right)$, and $c_{dir\_med}^{hosp\_severe}\left( i \right)$ are the unit direct medical costs in hosts aged $i$ per dengue case that is symptomatic nonhospitalized, hospitalized mild, and hospitalized severe, respectively; $N_{s,n,i}^{v,sympt\_non\_hosp}\left( t \right)$, $N_{s,n,i}^{v,hosp\_mild}\left( t \right)$, and $N_{s,n,i}^{v,hosp\_severe}\left( t \right)$ are the corresponding number of cases due to serotype $s$ of type $n$ in the cohort aged $i$ with vaccination status $v$ at time $t$.

#### Persistent dengue costs

The literature suggests that some patients may experience persistent dengue symptoms lasting beyond the acute phase of the infection [21]. To estimate the cost of persistent dengue, the number of symptomatic cases is multiplied by the proportion of cases resulting in long-term dengue, their duration, and their monthly cost. The cost of persistent dengue for hosts aged$i$, at time $t,$ is given by the following formula:

$$C_{pers}\left( i,t \right)={dur}_{pers}\cdot c_{pers}\cdot\overset{3}{\underset{n=1}{\sum}}\overset{4}{\underset{s=1}{\sum}}\overset{3}{\underset{v=1}{\sum}}N_{s,n,i}^{v,sympt}\left( t \right)\cdot p_{i}^{pers},$$

where $N_{s,n,i}^{v, sympt}\left( t \right)$ is the number of symptomatic cases with serotype $s$ of type $n$ in the cohort aged $i$ with vaccination status $v$ at time $t$, $p_{i}^{pers}$ is the proportion of cases resulting in long-term dengue in the cohort aged $i$, ${dur}_{pers}$ is the duration of long-term dengue expressed in months, and $c_{pers}$ is the monthly cost of treatment associated with persistent dengue.

#### Direct nonmedical costs

Direct nonmedical costs may include such items as treatment-related transportation, meals, accommodation, or any other item paid out of pocket by the patient and directly related to dengue treatment. The input unit costs can be differentiated by case severity (nonhospitalized, hospitalized mild, and hospitalized severe) and by age.

Direct nonmedical cost for hosts aged$i$, at time $t$, is given by the following formula:

$$C_{dir\_non\_med}\left( i,t \right)=c_{dir\_non\_med}^{sympt\_non\_hosp}\left( i \right)\cdot\overset{3}{\underset{n=1}{\sum}}\overset{4}{\underset{s=1}{\sum}}\overset{3}{\underset{v=1}{\sum}}N_{s,n,i}^{v,sympt\_non\_hosp}\left( t \right)+$$

$$c_{dir\_non\_med}^{hosp\_mild}\left( i \right)\cdot\overset{3}{\underset{n=1}{\sum}}\overset{4}{\underset{s=1}{\sum}}\overset{3}{\underset{v=1}{\sum}}N_{s,n,i}^{v,hosp\_mild}\left( t \right)+$$

$$c_{dir\_non\_med}^{hosp\_severe}\left( i \right)\cdot\overset{3}{\underset{n=1}{\sum}}\overset{4}{\underset{s=1}{\sum}}\overset{3}{\underset{v=1}{\sum}}N_{s,n,i}^{v,hosp\_severe}\left( t \right),$$

where $c_{dir\_non\_med}^{sympt\_non\_hosp}\left( i \right)$, $c_{dir\_non\_med}^{hosp\_mild}\left( i \right)$, and $c_{dir\_non\_med}^{hosp\_severe}\left( i \right)$ are the unit direct nonmedical costs in hosts aged $i$ per dengue case that is symptomatic nonhospitalized, hospitalized mild, and hospitalized severe, respectively, and $N_{s,n,i}^{v,sympt\_non\_hosp}\left( t \right)$, $N_{s,n,i}^{v,hosp\_mild}\left( t \right)$, and $N_{s,n,i}^{v,hosp\_severe}\left( t \right)$ were defined above.

## Indirect costs

Indirect costs include the productivity loss incurred by patients and their caregivers where applicable, and school absenteeism during the acute phase of dengue. Persistent dengue also has implications for productivity loss, as symptoms of persistent dengue may result in both presenteeism and absenteeism; however, owing to limited available data to quantify the productivity loss caused by persistent dengue, the model does not currently include this for in indirect costs. However, the model could potentially be updated should more data become available.

### Productivity loss

Productivity loss due to the acute phase of a dengue case is calculated from the number of workdays lost per dengue case (by the patient or a caregiver, for a specific age and case severity), the average daily wage, and the employment rate.

Productivity loss due to dengue death is calculated from the number of fatal cases, the average daily wage, the employment rate, and the number of workdays lost between the age of death and the retirement age.

The total productivity loss in hosts aged$i$, at time $t$, is given by the following formula:

$$C_{prod}\left( i,t \right)=\left( {wd}_{patient}^{sympt\_non\_hosp}\left( i \right)+{wd}_{caregiver}^{sympt\_non\_hosp}\left( i \right) \right)\cdot c_{daily wage}\cdot p^{empl}\cdot\overset{3}{\underset{n=1}{\sum}}\overset{4}{\underset{s=1}{\sum}}\overset{3}{\underset{v=1}{\sum}}N_{s,n,i}^{v,sympt\_non\_hosp}\left( t \right)+\left( {wd}_{patient}^{hosp\_mild}\left( i \right)+{wd}_{caregiver}^{hosp\_mild}\left( i \right) \right)\cdot c_{daily wage}\cdot p^{empl}\cdot\overset{3}{\underset{n=1}{\sum}}\overset{4}{\underset{s=1}{\sum}}\overset{3}{\underset{v=1}{\sum}}N_{s,n,i}^{v,hosp\_mild}\left( t \right)+\left( {wd}_{patient}^{hosp\_severe}\left( i \right)+{wd}_{caregiver}^{hosp\_severe}\left( i \right) \right)\cdot c_{daily wage}\cdot p^{empl}\cdot\overset{3}{\underset{n=1}{\sum}}\overset{4}{\underset{s=1}{\sum}}\overset{3}{\underset{v=1}{\sum}}N_{s,n,i}^{v,hosp\_severe}\left( t \right)+c_{prod}^{fatal}\left( i \right) \cdot\overset{3}{\underset{n=1}{\sum}}\overset{4}{\underset{s=1}{\sum}}\overset{3}{\underset{v=1}{\sum}}N_{s,n,i}^{v,severe\_fatal}\left( t \right)$$

where ${wd}_{patient}^{sympt\_non\_hosp}\left( i \right)$, ${wd}_{patient}^{hosp\_mild}\left( i \right)$, and ${wd}_{patient}^{hosp\_severe}\left( i \right)$ are the number of workdays lost by the patient per dengue case that is symptomatic nonhospitalized, hospitalized mild, and hospitalized severe, respectively, in a host aged $i$; ${wd}_{caregiver}^{sympt\_non\_hosp}\left( i \right)$, ${wd}_{caregiver}^{hosp\_mild}\left( i \right)$, and ${wd}_{caregiver}^{hosp\_severe}\left( i \right)$ are the number of workdays lost by the caregiver per dengue case that is symptomatic nonhospitalized, hospitalized mild, and hospitalized severe, respectively, in a host aged $i$; $c_{daily wage}$ is the average daily wage; $p^{empl}$ is the employment rate; $c_{prod}^{fatal}\left( i \right)$ is the cost of mortality-related productivity; and $N_{s,n,i}^{v,sympt\_non\_hosp}\left( t \right)$, $N_{s,n,i}^{v,hosp\_mild}\left( t \right)$, $N_{s,n,i}^{v,hosp\_severe}\left( t \right)$, and $N_{s,n,i}^{v,severe\_fatal}\left( t \right)$ are the number of infections with serotype $s$ of type $n$ in the cohort aged $i$ with vaccination status $v$, at time $t$ for symptomatic nonhospitalized, hospitalized mild, hospitalized severe, and severe fatal dengue, respectively.

The unit cost of mortality-related productivity is estimated with the following formula:

$c_{prod}^{fatal}\left( i \right)=c_{daily wage}\cdot p^{empl}\cdot{wd}^{annual}\cdot{wy}^{left}\left( i \right)$,

where $c_{daily wage}$ is the average daily wage, $p^{empl}$ is the employment rate, ${wd}^{annual}$ is the number of workdays in the year, and ${wy}^{left}\left( i \right)$ is the number of lost work years from the age of patient death to the retirement age for a host aged $i$ that would be left in the case of no fatality of the dengue case. If age $i$ is lower than the end of working age and greater or equal to the start of working age, then the parameter ${wy}^{left}\left( i \right)$may be estimated with the formula:

${wy}^{left}\left( i \right)=min\left( i^{we},L\left( i \right) \right)-i$,

where $i^{we}$ is the end of working age and $L\left( i \right)$ is the life expectancy for a host aged $i$. If age $i$ is lower than the end of working age and lower than the start of working age, then the parameter ${wy}^{left}\left( i \right)$may be estimated with the formula:

${wy}^{left}\left( i \right)=min\left( i^{we},L\left( i \right) \right)-i^{ws}+1$,

where$i^{we}$ is the end of working age, $i^{ws}$ is the start of working age, and $L\left( i \right)$ is the life expectancy for a host aged $i$.

When the discount rate for cost is included, then this cost of mortality-related productivity is discounted by taking into account the discount rate and the year during which the specific productivity loss occurs.

### School absenteeism

Based on the fact that schooling is publicly funded in many countries, and thus society values it at least as highly as its cost, the societal daily cost of absence from school was assumed to be equal to the cost per day of schooling [22]. The cost of school absenteeism is calculated from the number of symptomatic dengue cases (by severity) and the number of school days lost to a specific case and cost per school day lost.

In the model, the school absenteeism cost was estimated for hosts aged$i$, at time $t$, with the following formula:

$$C_{school}\left( i,t \right)={sd}^{sympt_{non_{hosp}}}\left( i \right)\cdot c_{sd}\cdot\overset{3}{\underset{n=1}{\sum}}\overset{4}{\underset{s=1}{\sum}}\overset{3}{\underset{v=1}{\sum}}N_{s,n,i}^{v,sympt_{non_{hosp}}}\left( t \right)+$$

$${sd}^{hosp_{mild}}\left( i \right)\cdot c_{sd}\cdot\overset{3}{\underset{n=1}{\sum}}\overset{4}{\underset{s=1}{\sum}}\overset{3}{\underset{v=1}{\sum}}N_{s,n,i}^{v,hosp_{mild}}\left( t \right)$$

$+{sd}^{hosp\_severe}\left( i \right)\cdot c_{sd}\cdot\overset{3}{\underset{n=1}{\sum}}\overset{4}{\underset{s=1}{\sum}}\overset{3}{\underset{v=1}{\sum}}N_{s,n,i}^{v,hosp\_severe}\left( t \right)$,

where ${sd}^{sympt\_non\_hosp}\left( i \right)$, ${sd}^{hosp\_mild}\left( i \right)$, and ${sd}^{hosp\_severe}\left( i \right)$ are the number of school days lost by patient per dengue case that is symptomatic nonhospitalized, hospitalized mild, and hospitalized severe, respectively, in a host aged $i$; $c_{sd}$ is the cost of 1 day of school absenteeism; and $N_{s,n,i}^{v,sympt\_non\_hosp}\left( t \right)$, $N_{s,n,i}^{v,hosp\_mild}\left( t \right)$, and $N_{s,n,i}^{v,hosp\_severe}\left( t \right)$ are the number of infections with serotype $s$ of type $n$ in the cohort aged $i$ with vaccination status $v$, at time $t$ for symptomatic nonhospitalized, hospitalized mild, and hospitalized severe dengue, respectively.

Total indirect costs are calculated as the sum of productivity loss costs and school absenteeism cost:

$C_{ind}\left( i,t \right)=C_{prod}\left( i,t \right)+C_{school}\left( i,t \right)$.

# Forward simulations

The outcome of vaccination may depend on the dengue transmission in the year of vaccine introduction (and more generally, during the simulation period). Thus, the exact timing of vaccine introduction may considerably affect the overall benefit of vaccination (especially in a scenario with catch-up vaccination), depending on when the vaccine is introduced within a natural multi-annual dengue cycle (lower or higher peak of dengue incidence). To account for the impact of this uncertainty around the initial condition, for each vaccination strategy, the model allows running of multiple forward simulations. In each simulation, the vaccination was introduced at a randomly selected year within a specified period simulated without vaccination (i.e., the second period from calibration, described in Supplemental Material S2). Assuming a simulation timeframe of 30 years from vaccination introduction, the vaccination can be introduced at any year ≥30 years before the end of the second period of calibration. Therefore, assuming that 100 years without vaccine were simulated, the main results of the model with vaccination represent the average of 70 simulations (each simulation for a different year of vaccine introduction).

Epidemiologic outcomes estimated in the model are the number of dengue infections over time (100 years for scenarios without vaccination and 30 years for scenarios with vaccination), by age (0–100 years), by infection type (primary, secondary, postsecondary), by serotype (DENV-1, DENV-2, DENV-3, DENV-4), by severity (see Sections 1.1.3 and 2.2.3), and by vaccination status, as well as seroprevalence (see Section 2.2.3) and number of newly vaccinated individuals over time, by age and by serostatus. For each simulation with vaccination, the corresponding period without vaccination is used for the comparison of outcomes generated with and without vaccination.

For scenarios with vaccination, additional outcomes were estimated:

- Number of infections avoided (versus no vaccination);
- Proportion (%) of infections avoided (versus no vaccination);
- Incremental costs (versus no vaccination).

The 95% confidence intervals were estimated around the mean of all model realizations.

# CHEERS 2022 checklist [23]

| **Topic** | **No.** | **Item** | **Location where item is reported** |
| --- | --- | --- | --- |
| **Title** |  |  |  |
|  | 1 | Identify the study as an economic evaluation and specify the interventions being compared. | Title of the manuscript: “Assessing the optimal vaccination strategies for dengue vaccine TAK-003, and its public health impact and cost-effectiveness: A case study in Thailand” |
| **Abstract** |  |  |  |
|  | 2 | Provide a structured summary that highlights context, key methods, results, and alternative analyses. | Abstract fully summarizes the background, aims, methodology, results, limitations, and conclusions within word count limit. |
| **Introduction** |  |  |  |
| **Background and objectives** | 3 | Give the context for the study, the study question, and its practical relevance for decision making in policy or practice. | Introduction (paragraphs 1-11). The introduction sets out the disease with specific regard to Thailand, the current care and prevention methods, the technology (TAK-003), and the aims of the study. |
| **Methods** |  |  |  |
| **Health economic analysis plan** | 4 | Indicate whether a health economic analysis plan was developed and where available. | N/A. A Health Economics Analysis Plan was not undertaken or registered. However, the epidemiological model evolved from previous studies and the cost-effectiveness analysis undertaken were standard to usual practice. |
| **Study population** | 5 | Describe characteristics of the study population (such as age range, demographics, socioeconomic, or clinical characteristics). | Methods. Transmission model section (Figure 1). Both host and vector populations are described, with limitations discussed. |
| **Setting and location** | 6 | Provide relevant contextual information that may influence findings. | Introduction (paragraph 11). The setting was Thailand, which is also made clear in the study title. |
| **Comparators** | 7 | Describe the interventions or strategies being compared and why chosen. | Methods. Transmission model section (paragraphs 1-2); Impact of Vaccination section (paragraph 1); Determination of TAK-003 efficacy (paragraph 1). The intervention is the implementation of TAK-003 in a national immunization program to prevent dengue under different vaccination strategies as discussed in the manuscript. The comparator is the absence of TAK-003. |
| **Perspective** | 8 | State the perspective(s) adopted by the study and why chosen. | Methods. Cost-effectiveness analysis section (paragraph 1). The perspective was societal, to encompass all relevant outcomes. A payer perspective was also performed in scenario analyses. |
| **Time horizon** | 9 | State the time horizon for the study and why appropriate. | Methods. Infection process and epidemiological outcomes section (Table 1). The time horizon was 20 years to capture the long term impact of vaccination. |
| **Discount rate** | 10 | Report the discount rate(s) and reason chosen. | Methods. Cost-effectiveness analysis section (paragraph 3). A discount rate of 3% was applied (consistent with Thai HTA guidance). |
| **Selection of outcomes** | 11 | Describe what outcomes were used as the measure(s) of benefit(s) and harm(s). | Methods. Infection process and epidemiological outcomes section (paragraphs 1-4 and Table 1). The outcomes measured were consistent with other models of this type. |
| **Measurement of outcomes** | 12 | Describe how outcomes used to capture benefit(s) and harm(s) were measured. | Methods. Infection process and epidemiological outcomes section (paragraphs 1-4 and Table 1). Disability-adjusted life years (DALYs) were ultimately derived from the epidemiological model which was informed with fully validated data where possible. |
| **Valuation of outcomes** | 13 | Describe the population and methods used to measure and value outcomes. | Methods. Cost-effectiveness analysis section (paragraph 3). DALYs for each health state were derived from the literature. |
| **Measurement and valuation of resources and costs** | 14 | Describe how costs were valued. | Methods. Cost-effectiveness analysis section (paragraphs 1-2). Both direct and indirect costs were included. These were derived from Thai specific sources and fully explained in the supplementary material (S2). |
| **Currency, price date, and conversion** | 15 | Report the dates of the estimated resource quantities and unit costs, plus the currency and year of conversion. | Methods. Cost-effectiveness analysis section (paragraphs 2-3). All costs were taken from the literature and were Thai specific where possible. Costs were converted to US dollars and adjusted to the price year 2021. |
| **Rationale and description of model** | 16 | If modelling is used, describe in detail and why used. Report if the model is publicly available and where it can be accessed. | Methods. All sections and paragraphs. The epidemiological model, data fitting, and cost-effectiveness were fully described in the main paper and also in the supplements (S1 and S2). |
| **Analytics and assumptions** | 17 | Describe any methods for analysing or statistically transforming data, any extrapolation methods, and approaches for validating any model used. | Methods. Determination of TAK-003 efficacy section (Figure 2). Construction and extrapolation of vaccine efficacy curves,  S2 document, section 1.1, Dengue incidence in Thailand (Figures A, B, C and D). Construction of single age curve. |
| **Characterising heterogeneity** | 18 | Describe any methods used for estimating how the results of the study vary for subgroups. | N/A. The implementation of TAK-003 in the national immunization program is a population level intervention. |
| **Characterising distributional effects** | 19 | Describe how impacts are distributed across different individuals or adjustments made to reflect priority populations. | N/A. The implementation of TAK-003 in the national immunization program is a population level intervention. |
| **Characterising uncertainty** | 20 | Describe methods to characterise any sources of uncertainty in the analysis. | S3 document, section 3.2. Scenario analyses section (Table 7). Scenario analyses were undertaken. |
| **Approach to engagement with patients and others affected by the study** | 21 | Describe any approaches to engage patients or service recipients, the general public, communities, or stakeholders (such as clinicians or payers) in the design of the study. | N/A. The implementation of TAK-003 in the national immunization program is a public health preventative intervention at the population level. |
| **Results** |  |  |  |
| **Study parameters** | 22 | Report all analytic inputs (such as values, ranges, references) including uncertainty or distributional assumptions. | Results. All subsections. All results reported using appropriate statistical annotation. |
| **Summary of main results** | 23 | Report the mean values for the main categories of costs and outcomes of interest and summarise them in the most appropriate overall measure. | Results. Cost-effectiveness analyses section (paragraph 1 and Table 3). Costs and benefits (DALYs) reported separately and aggregated as summary cost-effectiveness results, presented as incremental cost-effectiveness ratios (ΔCost/ΔDALY). |
| **Effect of uncertainty** | 24 | Describe how uncertainty about analytic judgments, inputs, or projections affect findings. Report the effect of choice of discount rate and time horizon, if applicable. | Results. Additional scenario analyses section (paragraph 1 and Figure 9). Results of scenario analysis presented. Note this was not done for cost-effectiveness outcomes as the primary interest was epidemiological, but in all scenarios TAK-003 dominated (<costs, >benefits). |
| **Effect of engagement with patients and others affected by the study** | 25 | Report on any difference patient/service recipient, general public, community, or stakeholder involvement made to the approach or findings of the study | N/A. No engagement of this type was made. As a preventative measure, this would not be appropriate. |
| **Discussion** |  |  |  |
| **Study findings, limitations, generalisability, and current knowledge** | 26 | Report key findings, limitations, ethical or equity considerations not captured, and how these could affect patients, policy, or practice. | Discussion, strengths and limitations and conclusion sections (all paragraphs). Extensive discussion on how results apply to Thailand in theory and in practice, with all major limitations discussed. |
| **Other relevant information** |  |  |  |
| **Source of funding** | 27 | Describe how the study was funded and any role of the funder in the identification, design, conduct, and reporting of the analysis | Financial disclosures statement section. |
| **Conflicts of interest** | 28 | Report authors conflicts of interest according to journal or International Committee of Medical Journal Editors requirements. | Competing interests section. |

*From:* Husereau D, Drummond M, Augustovski F, et al. Consolidated Health Economic Evaluation Reporting Standards 2022 (CHEERS 2022) Explanation and Elaboration: A Report of the ISPOR CHEERS II Good Practices Task Force. Value Health 2022;25. <doi:10.1016/j.jval.2021.10.008>

# References

**1.** Nguyet MN, Duong THK, Trung VT, Nguyen THQ, Tran CNB, Long VT, et al. Host and viral features of human dengue cases shape the population of infected and infectious *Aedes aegypti* mosquitoes. Proc Natl Acad Sci U S A. 2013; 110(22):9072-7. <https://doi.org/10.1073/pnas.1303395110> PMID: 23674683; PubMed Central PMCID: PMC3670336.

**2.** Duong V, Lambrechts L, Paul RE, Ly S, Lay RS, Long KC, et al. Asymptomatic humans transmit dengue virus to mosquitoes. Proc Natl Acad Sci U S A. 2015; 112(47):14688-93. <https://doi.org/10.1073/pnas.1508114112> PMID: 26553981; PubMed Central PMCID: PMC4664300.

**3.** Matangkasombut P, Manopwisedjaroen K, Pitabut N, Thaloengsok S, Suraamornkul S, Yingtaweesak T, et al. Dengue viremia kinetics in asymptomatic and symptomatic infection. Int J Infect Dis. 2020; 101:90-7. <https://doi.org/10.1016/j.ijid.2020.09.1446> PMID: 32992011.

**4.** Coudeville L, Garnett GP. Transmission dynamics of the four dengue serotypes in southern Vietnam and the potential impact of vaccination. Plos One. 2012; 7(12):e51244. <https://doi.org/10.1371/journal.pone.0051244> PMID: 23251466; PubMed Central PMCID: PMC3519629.

**5.** Bartley LM, Donnelly CA, Garnett GP. The seasonal pattern of dengue in endemic areas: Mathematical models of mechanisms. Trans R Soc Trop Med Hyg. 2002; 96(4):387-97. <https://doi.org/10.1016/S0035-9203(02)90371-8> PMID: 12497975.

**6.** Lourenço J, Recker M. Viral and epidemiological determinants of the invasion dynamics of novel dengue genotypes. PLoS Negl Trop Dis. 2010; 4(11):e894. <https://doi.org/10.1371/journal.pntd.0000894> PMID: 21124880; PubMed Central PMCID: PMC2990689.

**7.** Burattini MN, Chen M, Chow A, Coutinho FA, Goh KT, Lopez LF, et al. Modelling the control strategies against dengue in Singapore. Epidemiol Infect. 2008; 136(3):309-19. <https://doi.org/10.1017/s0950268807008667> PMID: 17540051; PubMed Central PMCID: PMC2870819.

**8.** Sridhar S, Luedtke A, Langevin E, Zhu M, Bonaparte M, Machabert T, et al. Effect of dengue serostatus on dengue vaccine safety and efficacy. N Engl J Med. 2018; 379(4):327-40. <https://doi.org/10.1056/NEJMoa1800820> PMID: 29897841.

**9.** Rivera L, Biswal S, Saez-Llorens X, Reynales H, Lopez-Medina E, Borja-Tabora C, et al. Three-year efficacy and safety of Takeda's dengue vaccine candidate (TAK-003). Clin Infect Dis. 2022; 75(1):107-17. <https://doi.org/10.1093/cid/ciab864> PMID: 34606595; PubMed Central PMCID: PMC9402653.

**10.** Rodriguez-Barraquer I, Mier-y-Teran-Romero L, Schwartz IB, Burke DS, Cummings DAT. Potential opportunities and perils of imperfect dengue vaccines. Vaccine. 2014; 32(4):514-20. <https://doi.org/10.1016/j.vaccine.2013.11.020> PMID: 24269318; PubMed Central PMCID: PMC4142437.

**11.** Srikiatkhachorn A, Yoon IK. Immune correlates for dengue vaccine development. Expert Rev Vaccines. 2016; 15(4):455-65. <https://doi.org/10.1586/14760584.2016.1116949> PMID: 26560015; PubMed Central PMCID: PMC5523864.

**12.** de Silva AM, Harris E. Which dengue vaccine approach is the most promising, and should we be concerned about enhanced disease after vaccination? The path to a dengue vaccine: Learning from human natural dengue infection studies and vaccine trials. Cold Spring Harb Perspect Biol. 2018; 10(6):a029371. <https://doi.org/10.1101/cshperspect.a029371> PMID: 28716891; PubMed Central PMCID: PMC5983190.

**13.** Katzelnick LC, Montoya M, Gresh L, Balmaseda A, Harris E. Neutralizing antibody titers against dengue virus correlate with protection from symptomatic infection in a longitudinal cohort. Proc Natl Acad Sci U S A. 2016; 113(3):728-33. <https://doi.org/10.1073/pnas.1522136113> PMID: 26729879; PubMed Central PMCID: PMC4725482.

**14.** Alexander LW, Ben-Shachar R, Katzelnick LC, Kuan G, Balmaseda A, Harris E, et al. Boosting can explain patterns of fluctuations of ratios of inapparent to symptomatic dengue virus infections. Proc Natl Acad Sci U S A. 2021; 118(14):e2013941118. <https://doi.org/10.1073/pnas.2013941118> PMID: 33811138; PubMed Central PMCID: PMC8040803.

**15.** Flasche S, Jit M, Rodríguez-Barraquer I, Coudeville L, Recker M, Koelle K, et al. The long-term safety, public health impact, and cost-effectiveness of routine vaccination with a recombinant, live-attenuated dengue vaccine (Dengvaxia): A model comparison study. PLOS Medicine. 2016; 13(11):e1002181. <https://doi.org/10.1371/journal.pmed.1002181> PMID: 27898668; PubMed Central PMCID: PMC5127514.

**16.** Carrasco LR, Lee LK, Lee VJ, Ooi EE, Shepard DS, Thein T, et al. Economic impact of dengue illness and the cost-effectiveness of future vaccination programs in Singapore. PLoS Negl Trop Dis. 2011; 5(12):e1426. <https://doi.org/10.1371/journal.pntd.0001426> PMID: 22206028; PubMed Central PMCID: PMC3243704.

**17.** Clark DV, Mammen Mp J, Nisalak A, Puthimethee V, Endy TP. Economic impact of dengue fever/dengue hemorrhagic fever in Thailand at the family and population levels. Am J Trop Med Hyg. 2005; 72(6):786-91. PMID: 15964964.

**18.** Meltzer MI, Rigau-Pérez J, Clark G, Reiter P, Gubler DJ. Using disability-adjusted life years to assess the economic impact of dengue in Puerto Rico: 1984-1994. Am J Trop Med Hyg. 1998; 59(2):265-71. <https://doi.org/10.4269/ajtmh.1998.59.265> PMID: 9715944.

**19.** Devleesschauwer B, Havelaar AH, Maertens de Noordhout C, Haagsma JA, Praet N, Dorny P, et al. Calculating disability-adjusted life years to quantify burden of disease. Int J Public Health. 2014; 59(3):565-9. <https://doi.org/10.1007/s00038-014-0552-z> PMID: 24752429.

**20.** Murray CJ. Quantifying the burden of disease: the technical basis for disability-adjusted life years. Bull World Health Organ. 1994; 72(3):429-45. PMID: 8062401; PubMed Central PMCID: PMC2486718.

**21.** Zeng W, Halasa-Rappel YA, Durand L, Coudeville L, Shepard DS. Impact of a nonfatal dengue episode on disability-adjusted life years: A systematic analysis. Am J Trop Med Hyg. 2018; 99(6):1458-65. <https://doi.org/10.4269/ajtmh.18-0309> PMID: 30277202; PubMed Central PMCID: PMC6283510.

**22.** Kongsin S, Jiamton S, Suaya JA, Vasanawathana S, Sirisuvan P, Shepard DS. Cost of dengue in Thailand. WHO Regional Office for South-East Asia 2010 [cited 2024 September 17]. Available from: <https://iris.who.int/handle/10665/170969>.

**23.** Husereau D, Drummond M, Augustovski F, de Bekker-Grob E, Briggs AH, Carswell C, et al. Consolidated Health Economic Evaluation Reporting Standards (CHEERS) 2022 Explanation and Elaboration: A Report of the ISPOR CHEERS II Good Practices Task Force. Value Health. 2022; 25(1):10-31. <https://doi.org/10.1016/j.jval.2021.10.008> PMID: 35031088.

1. The original notation found in Devleesschauwer et al. is preserved; parameter notation in this section is independent of the parameter notation in the rest of the model. [↑](#footnote-ref-1)
