# Supplementary material for: Vaccination strategies, public health impact and cost-effectiveness of dengue vaccine TAK-003: A modeling case study in Thailand
Source: PLoS Med. 2025 Jun 17;22(6):e1004631. doi: 10.1371/journal.pmed.1004631 (PMC12173404; doi:10.1371/journal.pmed.1004631)
Supplement: S2 File — (DOCX) [file pmed.1004631.s002.docx]

Assessing the optimal vaccination strategies for dengue vaccine TAK-003, and its public health impact and
cost-effectiveness: A case study in Thailand

**S2 Material: Model fitting and parameters**

This supplement (2) describes the model fitting and validation process, the model inputs, and the source and rationale for the use of these values. There are 2 additional supplements:

- **S1 Material** describes the structure of the epidemiological model used in the study in detail.
- **S3 Material** describes additional results not reported in the manuscript.

**Contents**

[1. Model fitting 4](#_Toc178261504)

[1.1. Dengue incidence in Thailand 4](#_Toc178261505)

[1.2. Calibration of β^VH^ and seasonality parameters 10](#_Toc178261506)

[1.3. Calibration of age-specific coefficients 11](#_Toc178261507)

[2. Model parameters 15](#_Toc178261508)

[2.1. Host population 15](#_Toc178261509)

[2.2. Vector population 17](#_Toc178261512)

[2.3. Dengue epidemiology 17](#_Toc178261513)

[2.3.1. Dengue incidence 17](#_Toc178261514)

[2.3.2. Severity structure 18](#_Toc178261515)

[2.3.3. Dengue natural history 21](#_Toc178261516)

[2.4. Efficacy of TAK-003 21](#_Toc178261517)

[2.4.1. Clinical overview 21](#_Toc178261518)

[2.4.2. TAK-003 efficacy 22](#_Toc178261519)

[2.5. Costs 28](#_Toc178261520)

[2.5.1. Costs of treatment 28](#_Toc178261521)

[2.5.1.1. Direct medical costs 29](#_Toc178261522)

[2.5.1.2. Direct nonmedical costs 29](#_Toc178261523)

[2.5.2. Indirect costs 30](#_Toc178261524)

[2.5.2.1. Cost of school absenteeism 30](#_Toc178261525)

[2.5.2.2. Productivity loss due to work absenteeism 31](#_Toc178261526)

[2.5.3. Cost of persistent dengue 32](#_Toc178261527)

[2.5.4. Cost of vaccination 32](#_Toc178261528)

[2.6. Quality of life 32](#_Toc178261529)

[3. Summary of parameters 34](#_Toc178261530)

[4. References 40](#_Toc178261531)

**List of figures**

[Fig A. Incidence rate with knots, illustrative example (Thailand, 2015). 8](#_Toc178261094)

[Fig B. Incidence rate with interpolated curves, illustrative example (Thailand, 2015). 9](#_Toc178261095)

[Fig C. Input data for approximation (Thailand, 2011–2020). 9](#_Toc178261096)

[Fig D. Incidence rate of symptomatic dengue (raw reported and smoothed; per 100,000). 10](#_Toc178261097)

[Fig E. Structure of the static model. 12](#_Toc178261098)

[Fig F. Age structure of the model population (versus the real age structure in 2020). 16](#_Toc178261099)

[Fig G. Thailand, life expectancy of the model population, by age. 17](#_Toc178261100)

[Fig H. Vaccine efficacy against nonhospitalized dengue, by serostatus and infection post vaccination. 24](#_Toc178261101)

[Fig I. Power curve fitted to vaccine efficacy against hospitalized dengue observed in the trial. 25](#_Toc178261102)

[Fig J. Modified vaccine efficacy against nonhospitalized dengue, by serostatus at vaccination and virus serotype. 27](#_Toc178261103)

**List of tables**

[Table 1. Number of dengue cases (DF + DHF + DSS) in Thailand between 2011 and 2020. 5](#_Toc178261178)

[Table 2. Population size in Thailand between 2011 and 2020. 6](#_Toc178261179)

[Table 3. Calculated average dengue incidences in Thailand. 6](#_Toc178261180)

[Table 4. Age-specific transmission coefficients, by age. 14](#_Toc178261181)

[Table 5. Thailand, probability of all-cause death, by age (2019). 15](#_Toc178261182)

[Table 6. Thailand, severity structure inputs. 18](#_Toc178261183)

[Table 7. Thailand, annual number of hospitalized cases (with and without expansion), by year. 19](#_Toc178261184)

[Table 8. Thailand, probability of hospitalization with symptomatic dengue (before and after adjustment). 19](#_Toc178261185)

[Table 9. Thailand, % DHF among dengue cases. 20](#_Toc178261186)

[Table 10. Thailand, probability of death with severe dengue. 20](#_Toc178261187)

[Table 11. Point estimates for efficacy against nonhospitalized dengue, by serostatus at vaccination. 22](#_Toc178261188)

[Table 12. Vaccine efficacy against nonhospitalized dengue, by serostatus at vaccination. 23](#_Toc178261189)

[Table 13. Vaccine efficacy against hospitalized dengue, by year and overall. 24](#_Toc178261190)

[Table 14. Cumulative 48-month post–second dose vaccine efficacy against symptomatic nonhospitalized dengue infection, by serotype and serostatus at vaccination. 26](#_Toc178261191)

[Table 15. Assumed vaccine efficacy against hospitalized dengue, by serostatus and dengue serotype. 28](#_Toc178261192)

[Table 16. Thailand, direct medical and nonmedical cost (per case), by case severity. 29](#_Toc178261193)

[Table 17. Thailand, direct medical costs, per unit. 29](#_Toc178261194)

[Table 18. Thailand, resource use (per case), by case severity (as in Fitzpatrick et al. [2017] [35]). 29](#_Toc178261195)

[Table 19. Thailand, direct nonmedical cost (per case), by case severity (as in Suaya et al. [2009] [36]). 30](#_Toc178261196)

[Table 20. Thailand, number of school days lost (per case), by case severity. 30](#_Toc178261197)

[Table 21. Thailand, number of workdays lost by patient (per case), by case severity. 31](#_Toc178261198)

[Table 22. Thailand, quality-of-life inputs. 32](#_Toc178261199)

[Table 23. Summary of parameters used in the model. 34](#_Toc178261200)

# Model fitting

The model is fitted to the average age-specific and seasonal incidence rates of symptomatic dengue, reported by the Thailand Ministry of Health for the period between 2011 and 2020 [1]. The incidence rates are informed by the case counts reported in the national or regional surveillance systems, and adjusted by a country-specific expansion factor (i.e., the degree of underreporting) that can be derived from the literature [2], as it is generally recognized that the reported cases only represent a fraction of actual dengue incidence (due to patients not seeking care, misdiagnoses, or the failure to meet all the criteria required for case definition) [3-6]. The expansion factors are usually estimated by comparing the incidence recorded in prospective cohort studies with the incidence reported to the passive surveillance systems [4]. The overall expansion factor of 8.37 [4] was used in the fitting of the probability of virus transmission and the shape of the seasonal curve.

The fitted parameters include the probability of virus transmission from an infectious host to a susceptible vector upon a bite ($\beta^{VH}$), as well as the amplitude and horizontal shift of the seasonal sine function used to define the vector recruitment rate at each day of the simulation ($a_{seas}$ and $b_{seas}$, respectively). More details are presented in Section 1.1.

Age-specific coefficients $\beta_{i}$ used in the calculation of the force of infection for hosts are calibrated separately using a simpler static model.

## Dengue incidence in Thailand

Based on reported number of dengue cases (Table 1) and Thai population size in 2011–2020 (Table 2), derived from the reports published by the Ministry of Health in Thailand and National Statistical Office of Thailand, average incidence rate was estimated (Table 3).

###### Table 1. Number of dengue cases (DF + DHF + DSS) in Thailand between 2011 and 2020.

|  | **2011** | **2012** | **2013** | **2014** | **2015** | **2016** | **2017** | **2018** | **2019** | **2020** |
| --- | --- | --- | --- | --- | --- | --- | --- | --- | --- | --- |
| **Total** | **69,800** | **78,337** | **154,444** | **41,082** | **144,952** | **63,931** | **53,961** | **87,212** | **131,157** | **72,130** |
| **By age group, years** | | | | | | | | | | |
| 0–4 | 4,394 | 13,026 | 8,298 | 2,772 | 6,617 | 4,158 | 3,183 | 5,487 | 7,561 | 3,873 |
| 5–9 | 11,103 | 14,215 | 21,220 | 6,396 | 17,545 | 9,086 | 7,020 | 13,900 | 22,050 | 10,556 |
| 10–14 | 16,559 | 16,799 | 32,916 | 8,080 | 27,483 | 11,029 | 10,129 | 18,172 | 28,775 | 15,282 |
| 15–24 | 18,972 | 18,558 | 45,045 | 10,753 | 41,039 | 15,921 | 13,140 | 22,286 | 32,861 | 18,626 |
| 25–34 | 8,947 | 7,724 | 20,682 | 5,641 | 22,624 | 9,877 | 6,332 | 12,029 | 17,143 | 10,221 |
| 35–44 | 4,885 | 3,867 | 11,995 | 3,406 | 13,747 | 6,027 | 8,932 | 6,847 | 9,468 | 5,609 |
| 45–54 | 2,755 | 2,240 | 7,862 | 2,145 | 8,566 | 3,946 | 2,517 | 4,135 | 6,234 | 3,663 |
| 55–64 | 1,377 | 1,230 | 4,247 | 1,174 | 4,882 | 2,473 | 1,669 | 2,703 | 4,363 | 2,543 |
| ≥65 | 807 | 678 | 2,179 | 714 | 2,448 | 1,413 | 1,039 | 1,653 | 2,702 | 1,757 |
| **By month** | | | | | | | | | | |
| January | 2,889 | 1,961 | 8,172 | 1,748 | 2,639 | 7,068 | 3,410 | 2,244 | 5,797 | 3,883 |
| February | 2,256 | 2,024 | 6,099 | 1,528 | 2,183 | 4,060 | 2,458 | 1,996 | 4,984 | 2,787 |
| March | 2,403 | 2,385 | 7,707 | 1,672 | 2,716 | 3,712 | 2,152 | 2,606 | 5,369 | 2,539 |
| April | 3,255 | 3,071 | 9,000 | 1,382 | 3,431 | 2,152 | 2,126 | 3,104 | 4,931 | 3,111 |
| May | 8,058 | 4,920 | 15,660 | 2,474 | 8,065 | 1,981 | 3,708 | 7,291 | 8,350 | 5,805 |
| June | 12,284 | 8,361 | 28,377 | 4,357 | 12,913 | 3,741 | 7,102 | 13,612 | 18,697 | 9,242 |
| July | 12,082 | 10,032 | 31,132 | 5,911 | 17,735 | 7,491 | 8,385 | 14,125 | 22,413 | 15,828 |
| August | 9,784 | 9,608 | 22,253 | 5,751 | 21,852 | 10,258 | 7,561 | 12,420 | 18,209 | 12,527 |
| September | 6,244 | 8,821 | 11,844 | 4,845 | 21,181 | 7,995 | 5,049 | 9,081 | 14,367 | 7,915 |
| October | 4,115 | 9,547 | 6,838 | 4,326 | 18,058 | 6,336 | 4,093 | 7,592 | 12,848 | 4,324 |
| November | 4,143 | 10,232 | 5,002 | 4,229 | 23,472 | 5,253 | 4,047 | 7,256 | 10,418 | 2,967 |
| December | 2,287 | 7,375 | 2,360 | 2,859 | 10,707 | 3,884 | 1,958 | 5,885 | 4,774 | 1,591 |
| DF, dengue fever; DHF, dengue hemorrhagic fever; DSS, dengue septic shock syndrome.  Source: Ministry of Public Health. National Disease Surveillance (Report 506) [7]. | | | | | | | | | | |

###### Table 2. Population size in Thailand between 2011 and 2020.

|  | **2011** | **2012** | **2013** | **2014** | **2015** | **2016** | **2017** | | **2018** | **2019** | **2020** |
| --- | --- | --- | --- | --- | --- | --- | --- | --- | --- | --- | --- |
| **Total** | **63,977,185** | **64,075,986** | **64,076,033** | **64,955,313** | **65,124,716** | **65,426,907** | **66,060,027** | | **66,301,242** | **66,486,458** | **66,301,242** |
| **By age group, years** | | | | | | | |  |  |  |  |
| 0–4 | 3,898,787 | 3,861,328 | 3,804,745 | 3,795,696 | 3,730,296 | 3,621,572 | 3,504,388 | | 3,390,953 | 3,263,101 | 3,133,695 |
| 5–9 | 4,043,377 | 4,031,171 | 4,003,252 | 4,002,979 | 3,950,545 | 3,946,672 | 3,972,855 | | 3,941,765 | 3,900,415 | 3,819,804 |
| 10–14 | 4,410,826 | 4,210,038 | 4,079,305 | 4,088,081 | 4,044,227 | 4,046,455 | 4,080,963 | | 4,079,323 | 4,049,503 | 3,985,518 |
| 15–24 | 9,607,135 | 9,635,586 | 9,577,573 | 9,577,908 | 9,444,569 | 9,285,541 | 9,160,753 | | 9,002,107 | 8,872,832 | 8,657,604 |
| 25–34 | 10,249,277 | 10,004,244 | 9,809,780 | 9,743,908 | 9,621,055 | 9,560,595 | 9,555,925 | | 9,519,820 | 9,504,453 | 9,481,089 |
| 35–44 | 10,855,585 | 10,802,642 | 10,627,554 | 10,691,905 | 10,629,436 | 10,551,968 | 10,547,302 | | 10,482,835 | 10,375,731 | 10,230,834 |
| 45–54 | 9,464,836 | 9,639,194 | 9,809,854 | 10,042,291 | 10,150,490 | 10,309,292 | 10,464,815 | | 10,506,318 | 10,507,456 | 10,433,824 |
| 55–64 | 6,132,744 | 6,389,597 | 6,494,685 | 6,814,693 | 7,089,235 | 7,354,352 | 7,667,059 | | 7,951,252 | 8,250,133 | 8,526,861 |
| ≥65 | 5,314,617 | 5,502,186 | 5,869,284 | 6,197,852 | 6,464,862 | 6,750,459 | 7,105,967 | | 7,426,869 | 7,762,834 | 8,032,013 |
| Source: National Statistic Office of Thailand [8]. | | | | | | | | | | | |

###### Table 3. Calculated average dengue incidences in Thailand.

|  | **2011** | **2012** | **2013** | **2014** | **2015** | **2016** | **2017** | **2018** | **2019** | **2020** | **Average** |
| --- | --- | --- | --- | --- | --- | --- | --- | --- | --- | --- | --- |
| **Total** | **109** | **122** | **241** | **63** | **223** | **98** | **82** | **132** | **197** | **109** | **138** |
| **By age group, years** | | | | | | | | | | | |
| 0–4 | 113 | 337 | 218 | 73 | 177 | 115 | 91 | 162 | 232 | 124 | 164 |
| 5–9 | 275 | 353 | 530 | 160 | 444 | 230 | 177 | 353 | 565 | 276 | 336 |
| 10–14 | 375 | 399 | 807 | 198 | 680 | 273 | 248 | 445 | 711 | 383 | 452 |
| 15–24 | 197 | 193 | 470 | 112 | 435 | 171 | 143 | 248 | 370 | 215 | 256 |
| 25–34 | 87 | 77 | 211 | 58 | 235 | 103 | 66 | 126 | 180 | 108 | 125 |
| 35–44 | 45 | 36 | 113 | 32 | 129 | 57 | 85 | 65 | 91 | 55 | 71 |
| 45–54 | 29 | 23 | 80 | 21 | 84 | 38 | 24 | 39 | 59 | 35 | 43 |
| 55–64 | 22 | 19 | 65 | 17 | 69 | 34 | 22 | 34 | 53 | 30 | 37 |
| ≥65 | 15 | 12 | 37 | 12 | 38 | 21 | 15 | 22 | 35 | 22 | 23 |
| **By month** | | | | | | | | | | | |
| January | 4 | 3 | 13 | 3 | 4 | 11 | 5 | 3 | 9 | 6 | 6 |
| February | 3 | 3 | 9 | 2 | 3 | 6 | 4 | 3 | 8 | 4 | 5 |
| March | 4 | 4 | 12 | 3 | 4 | 6 | 3 | 4 | 8 | 4 | 5 |
| April | 5 | 5 | 14 | 2 | 5 | 3 | 3 | 5 | 8 | 5 | 5 |
| May | 12 | 8 | 24 | 4 | 12 | 3 | 6 | 11 | 13 | 9 | 10 |
| June | 19 | 13 | 43 | 7 | 20 | 6 | 11 | 21 | 29 | 14 | 18 |
| July | 19 | 15 | 48 | 9 | 27 | 11 | 13 | 22 | 34 | 24 | 22 |
| August | 15 | 15 | 34 | 9 | 33 | 16 | 12 | 19 | 28 | 19 | 20 |
| September | 10 | 14 | 18 | 7 | 32 | 12 | 8 | 14 | 22 | 12 | 15 |
| October | 6 | 15 | 10 | 7 | 28 | 10 | 6 | 12 | 20 | 7 | 12 |
| November | 6 | 16 | 8 | 6 | 36 | 8 | 6 | 11 | 16 | 5 | 12 |
| December | 4 | 11 | 4 | 4 | 16 | 6 | 3 | 9 | 7 | 2 | 7 |

In the age optimization analysis, to overcome the limitation of the incidence data grouped by age in irregular intervals and in the absence of single-year incidence data, an incidence curve by single year of age was approximated (Fig D).

The estimation of the single-age incidence curve was achieved by applying a data-specific approach combining interpolation and approximation techniques. Due to a noticeable variability in incidence rate values across years, an interpolation step [9] was performed for each year separately. To estimate the incidence rate curve for each interval (age group), 2 knots (i.e., data points placed on both borders of interval) were derived using the following formula:

$K_{i} =\frac{\left( \frac{r_{i}}{w_{i}}+\frac{r_{i+1}}{w_{i+1}} \right)}{\left( \frac{1}{w_{i}}+\frac{1}{w_{i+1}} \right)}$,

where:

$K_{i}$ – knot value between$i_{th}$ and $i_{th+1}$ intervals;

$r_{i}$ – incidence rate value for$i_{th}$interval; and

$w_{i}$ – the width of the $i_{th}$interval.

The knot magnitude should be interpreted as the expected incidence rate value at the junction of 2 adjacent intervals (age groups; Fig A). To account for differences in interval widths, the knot value was calculated as a weighted average rather than a simple average. The weight was defined as reciprocal of a given interval length; thus, greater weight was given to narrower intervals, which allowed the ability to account for the uncertainty stemming from the different interval widths. As a result, 2 knots were obtained for each interval. The incidence equal to 0 was assumed for borders knots, i.e., at birth (0 years) and age of 100 years (Fig D).

##### Fig A. Incidence rate with knots, illustrative example (Thailand, 2015).


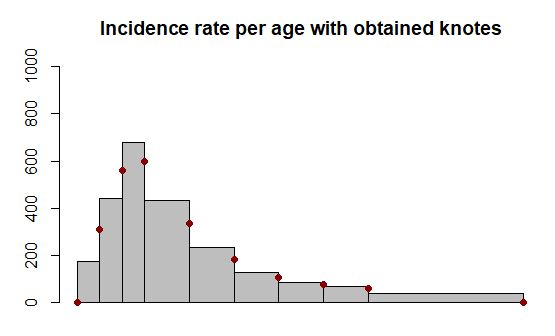


Afterward, a curve crossing the 2 knots was constructed separately for each interval, assuming the shape of the second-order polynomial (Fig B). To reflect the incidence magnitude for the given interval, the additional condition was incorporated, i.e., it was implied that the area under the curve (corresponding to predicted incidence) and the respective bar (corresponding to reported incidence) are equal.

##### Fig B. Incidence rate with interpolated curves, illustrative example (Thailand, 2015).


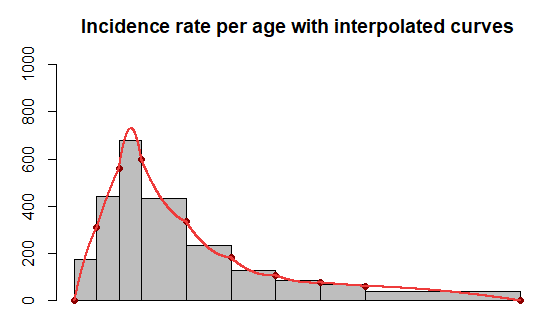


The obtained interpolated polynomials for each year were used to generate data inputs for the approximation step [10]. The exemplary results are presented in Fig C.

##### Fig C. Input data for approximation (Thailand, 2011–2020).


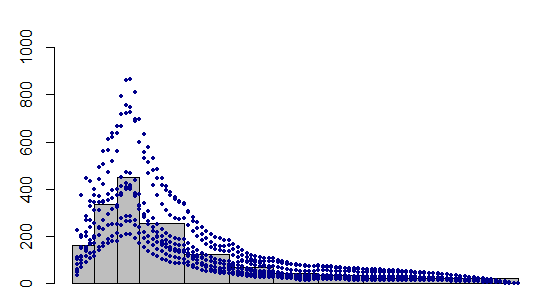


Seven different curves were considered to find the best-fitting curve to interpolated data points, namely the following: inverse Gompertz, Weibull, combined Weibull, chi-square test, generalized gamma distribution, and Frechet and Levy. For each curve, parameters were obtained using a weighted Levenberg-Marquardt algorithm [11]. The principle of weighting was the same as applied in the interpolation step, i.e., a greater weight was given to points from narrower intervals. Based on the goodness-of-fit measure (interval weighted mean absolute error), the combined Weibull curve was chosen.

##### Fig D. Incidence rate of symptomatic dengue (raw reported and smoothed; per 100,000).


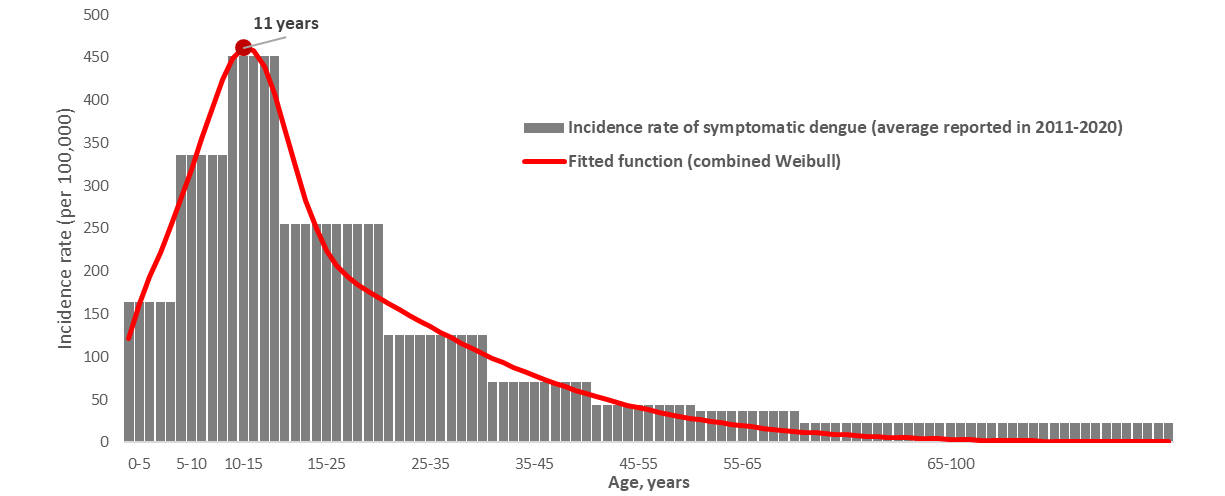


## Calibration of β^VH^ and seasonality parameters

The following parameters are calibrated:

- The parameter $\beta^{VH}$(the probability of virus transmission from an infectious mosquito to a susceptible host, given a bite) is calibrated by fitting the model to the age-specific incidence rate of symptomatic dengue, using the R package *optimize*.
- Seasonality parameters (the amplitude $a_{seas}$ and the horizontal shift $b_{seas}$ of the sine function applied to the size of the vector population) is calibrated by fitting the model to the incidence rate of symptomatic dengue by calendar month, using the R package *optim*.

The calibration of these parameters is performed iteratively. First, the probability of virus transmission is calibrated, assuming no seasonality; at the second iteration, the seasonality parameters are calibrated using the best-fitting value of the probability of virus transmission from the first iteration; and at the third iteration, the probability of virus transmission is re-calibrated given the best-fitting seasonality parameters from the second iteration. This process is repeated until the goodness of fit, determined by log-likelihood, can no longer be improved.

At each iteration, the calibration is performed dynamically (rather than at steady state); the model is run numerous times, with the values of calibrated parameter(s) being adjusted at each run based on the goodness of fit predicted in the previous runs.

For each run, the model is initialized by introducing a single infectious mosquito into a population of fully susceptible hosts. Dengue transmission is then simulated over 2 time periods: a relatively long burn-in period (e.g., 650 years), aimed at letting the model equilibrate, and a relatively short second period (e.g., 100 years), which is used to assess the goodness of fit.

The goodness of fit is defined by Poisson log-likelihood, which is maximized in the calibration process [12].

When calibrating the parameter $\beta^{VH}$, the mean predicted age-specific incidence rate over the second period of the simulation is compared with the actual incidence rate (i.e., the reported rate multiplied by the expansion factor) using a positive log-likelihood given by the following formula:

${Log\text{-}likelihood}_{incidence}=\underset{I}{\sum}\left( n_{I}^{obs} ln\left( n_{I}^{pred} \right)-n_{I}^{pred} \right)$,

where $n_{I}^{obs}$ denotes the actual incidence rate of symptomatic dengue in the age group $I$ and is estimated as $n_{I}^{obs}$=$n_{I}^{rep}\cdot EF$, where $n_{I}^{rep}$ denotes the rate of reported incidence in the age group $I$ and $EF$ denotes the expansion factor; $n_{I}^{pred}$ denotes the incidence rate of symptomatic dengue in the age group $I$ predicted in a given model run and averaged over the analysis period.

Similarly, when calibrating the seasonality parameters $a_{seas}$ and $b_{seas}$, the mean predicted incidence by calendar month is compared with the actual incidence rate by calendar month (also corrected by the value of the expansion factor). The log-likelihood is given by:

${Log\text{-}likelihood}_{seasonality}=\overset{12}{\underset{m=1}{\sum}}\left( n_{m}^{obs} ln\left( n_{m}^{pred} \right)-n_{m}^{pred} \right)$,

where $m$ indicates the month of the year; $n_{m}^{obs}$ denotes the actual incidence rate of symptomatic dengue in month $m$ and is estimated as $n_{m}^{obs}$=$n_{m}^{rep}\cdot EF$, where $n_{m}^{rep}$ denotes the reported incidence rate of symptomatic dengue in month $m$ and $EF$ denotes the expansion factor. The parameter $n_{m}^{pred}$ denotes the average incidence rate of symptomatic dengue in month $m$ over the analysis period.

## Calibration of age-specific coefficients

To reproduce the differences in incidence rates observed between the age groups, the force of infection for hosts includes an age-specific coefficient $\beta_{i}$. Given the large number of these coefficients (1 per age group used to define the observed incidence rate), calibrating them at the same time as the parameter $\beta^{VH}$ proved prohibitively time consuming. Therefore, they are calibrated outside the dynamic model using a simpler static model, which is based on the same key assumptions and is populated with the same input values as the dynamic model. The definition of the static model and the approach to the calibration of the age-specific coefficients is described below. The resulting values of $\beta_{i}$ are then input into the dynamic model for the calibration of $\beta^{VH}$ and seasonality parameters.

The static model is structured as a Markov process and tracks 1 cohort through 8 health states, as displayed in Fig E. The probability of infection is assumed to be constant over time. Combined with the assumption that the population size and age structure are constant over time (in line with the dynamic model), the pathway of each cohort is identical in the absence of vaccination. Therefore, it is sufficient to simulate a single cohort from birth to death to calibrate the probability of infection required to estimate the age coefficient $\beta_{i}$.

##### Fig E. Structure of the static model. All-cause death is possible from any health state. In the post-CP states, there is an immunization to the infection, e.g., being in the first infection post CP means that person is immune to 1 serotype and susceptible to another 3 serotypes. CP, cross-protection; inf, infection.


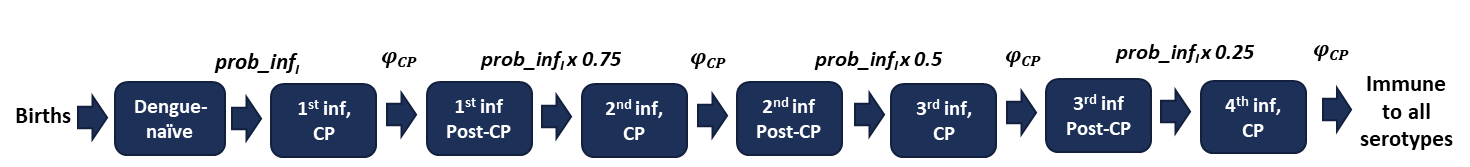


The pathway displayed in Fig E is based on the same key assumptions as the dynamic model (albeit a simplified representation). It is assumed that the hosts are born susceptible and can have up to 4 dengue infections during their lifetime. Unlike in the dynamic model, the infections in the static model are modeled as consecutive and not serotype specific.

Each infection is followed by a period of cross-protection, when the host is completely protected against infection and stays in the health state labeled “cross-protection (CP)”. After the period of cross-protection, the host transitions to a state labeled “Post-CP,” where acquisition of a new infection is possible.

The transitions from the state “Dengue-naive” and the state labeled “Post-CP” to the state labeled “CP” are driven by the probability of acquiring a new infection. This probability is assumed to be age specific and constant over time. The probability of primary infection for the age group $I$ is denoted by ${prob\_inf}_{I}$ and is obtained by fitting the model to empirical data.

Once immune to 1 serotype, the individuals are assumed to be immune to 25% of mosquito bites. Thus, in the individuals already immune to 1, 2, or 3 serotypes, the probability of a new infection is equal to 75%, 50%, or 25% of the original probability of primary infection, respectively.

The transition probabilities from “CP” states to “Post-CP” states are defined by the expiration of cross-protection (calculated in the same way as in the dynamic model).

As described above, the probabilities of the second, third, and fourth infections are calculated from the probability of the first infection. Therefore, only the probability of the first infection is calibrated for each age group.

The calibration is performed independently for each age group used to define the target incidence rate and is aimed at minimizing the distance between the predicted and observed rate of symptomatic dengue in a specific age group. The 1-dimensional optimization using the R package *optimize* requires multiple simulations of the age group of interest (e.g., if the age group “0–4 years old” is used to specify the target incidence, the simulation only includes the first 5 years of life). Each run is based on a predetermined value of the probability of infection; the quality of fit for each run is then assessed by comparing the distance between the simulated and reported rates of symptomatic dengue, defined as follows:

${(n_{I}^{pred}-n_{I}^{obs})}^{2}$,

where $n_{I}^{pred}$ denotes the incidence rate of symptomatic dengue in the age group $I$ predicted at a given model run; $n_{i}^{obs}$ denotes the actual incidence rate of symptomatic dengue in the age group $i$ and is estimated as $n_{I}^{obs}$=$n_{I}^{rep}\cdot EF$, where $n_{I}^{rep}$ denotes the rate of reported incidence in the age group $I$ and $EF$ denotes the expansion factor.

The parameter $\beta_{i}$ required in the dynamic model is estimated as the best-fitting probability of primary infection in the age group $I$ divided by the best-fitting probability of primary infection in the age group “50 years old”:

$\beta_{I}=\frac{{prob\_inf}_{I}}{{prob\_inf}_{51}}$,

where $\beta_{I}$ is the age coefficient for the age group $I$; ${prob\_inf}_{I}$ is the probability of primary infection in the age group $I$; and ${prob\_inf}_{51}$ is the probability of primary infection in the age group “50 years old.” The parameter $\beta_{i}$ (where $i$ indicates a single year of age) is informed by the parameter $\beta_{I}$ (where $I$ is the age group, which includes the age $i$).

###### Table 4. Age-specific transmission coefficients, by age.

| **Age, years** | **Age coefficient** | **Age, years** | **Age coefficient** | **Age, years** | **Age coefficient** |
| --- | --- | --- | --- | --- | --- |
| 0 | 1.2923 | 34 | 2.0385 | 68 | 0.2862 |
| 1 | 1.7129 | 35 | 1.9755 | 69 | 0.2642 |
| 2 | 2.0135 | 36 | 1.9110 | 70 | 0.2437 |
| 3 | 2.2773 | 37 | 1.8453 | 71 | 0.2247 |
| 4 | 2.5439 | 38 | 1.7788 | 72 | 0.2069 |
| 5 | 2.8336 | 39 | 1.7115 | 73 | 0.1902 |
| 6 | 3.1548 | 40 | 1.6439 | 74 | 0.1749 |
| 7 | 3.5047 | 41 | 1.5761 | 75 | 0.1607 |
| 8 | 3.8684 | 42 | 1.5085 | 76 | 0.1475 |
| 9 | 4.2198 | 43 | 1.4413 | 77 | 0.1353 |
| 10 | 4.5229 | 44 | 1.3748 | 78 | 0.1240 |
| 11 | 4.7369 | 45 | 1.3090 | 79 | 0.1129 |
| 12 | 4.8242 | 46 | 1.2443 | 80 | 0.1033 |
| 13 | 4.7619 | 47 | 1.1810 | 81 | 0.0944 |
| 14 | 4.5525 | 48 | 1.1191 | 82 | 0.0877 |
| 15 | 4.2287 | 49 | 1.0587 | 83 | 0.0801 |
| 16 | 3.8473 | 50 | 1.0000 | 84 | 0.0732 |
| 17 | 3.4730 | 51 | 0.9431 | 85 | 0.0660 |
| 18 | 3.1569 | 52 | 0.8881 | 86 | 0.0601 |
| 19 | 2.9232 | 53 | 0.8352 | 87 | 0.0547 |
| 20 | 2.7690 | 54 | 0.7842 | 88 | 0.0498 |
| 21 | 2.6746 | 55 | 0.7354 | 89 | 0.0453 |
| 22 | 2.6159 | 56 | 0.6887 | 90 | 0.0411 |
| 23 | 2.5744 | 57 | 0.6446 | 91 | 0.0370 |
| 24 | 2.5377 | 58 | 0.6022 | 92 | 0.0336 |
| 25 | 2.5005 | 59 | 0.5619 | 93 | 0.0304 |
| 26 | 2.4608 | 60 | 0.5236 | 94 | 0.0275 |
| 27 | 2.4180 | 61 | 0.4875 | 95 | 0.0264 |
| 28 | 2.3719 | 62 | 0.4533 | 96 | 0.0230 |
| 29 | 2.3228 | 63 | 0.4211 | 97 | 0.0208 |
| 30 | 2.2708 | 64 | 0.3907 | 98 | 0.0188 |
| 31 | 2.2162 | 65 | 0.3607 | 100 | 0.0170 |
| 32 | 2.1591 | 66 | 0.3348 |  |  |
| 33 | 2.0998 | 67 | 0.3097 |  |  |

# Model parameters

## Host population

The **total population size** in Thailand was assumed to be equal to 66,486,458, based on data from the National Disease Surveillance (Report 506) [13]. The year for the population counts (2019) was selected to coincide with the year of the data on all-cause mortality described below.

Age-specific **probability of all-cause death** was informed by the life tables available on the World Health Organization (WHO) website (see third column of Table 5) [14]. Because these values were grouped by age, they represented the probability of dying *while in a specific age group* (i.e., over a period that corresponded to the age group’s length); they were extended to single years of age. The final values, representing the *annual* probability of dying at a specific age are shown in the last column of Table 5.

###### Table 5. Thailand, probability of all-cause death, by age (2019).

| **Age, years** | | **Probability of dying**  **(while in the age group)** | **Probability of dying**  **(annual)** |
| --- | --- | --- | --- |
| **Low** | **High** |  |  |
| 0 | 0 | 0.76% | 0.764% |
| 1 | 4 | 0.13% | 0.032% |
| 5 | 9 | 0.16% | 0.033% |
| 10 | 14 | 0.26% | 0.051% |
| 15 | 19 | 0.44% | 0.088% |
| 20 | 24 | 0.60% | 0.120% |
| 25 | 29 | 0.80% | 0.161% |
| 30 | 34 | 1.04% | 0.209% |
| 35 | 39 | 1.27% | 0.254% |
| 40 | 44 | 1.52% | 0.306% |
| 45 | 49 | 1.87% | 0.376% |
| 50 | 54 | 2.45% | 0.495% |
| 55 | 59 | 3.24% | 0.656% |
| 60 | 64 | 4.72% | 0.963% |
| 65 | 69 | 7.13% | 1.469% |
| 70 | 74 | 10.73% | 2.246% |
| 75 | 79 | 16.34% | 3.505% |
| 80 | 84 | 24.91% | 5.569% |
| 85 | 99 | 99.9%^a^ | 35.062% |
| **100** | 100 | 99.9% | 100% |
| ^a^The probability of dying in the last age group (85–100 years) was set to 99.9% to prevent the entire cohort from dying at the age of 85 years; the final probability of dying at the age of 100 years was set to 100%. | | | |

The **size of birth cohort** required to maintain the constant size and age structure of the model population was estimated at 871,952 individuals who would enter the model every year (see S1 Material, Section 1.1.1 for more details). Based on this number and on the probability of dying presented above, the final age structure of the model population could be simulated (see Fig F below).

##### Fig F. Age structure of the model population (versus the real age structure in 2020). Real population counts by single year of age are based on the estimates for 2020, as published by the National Statistical Office [15].


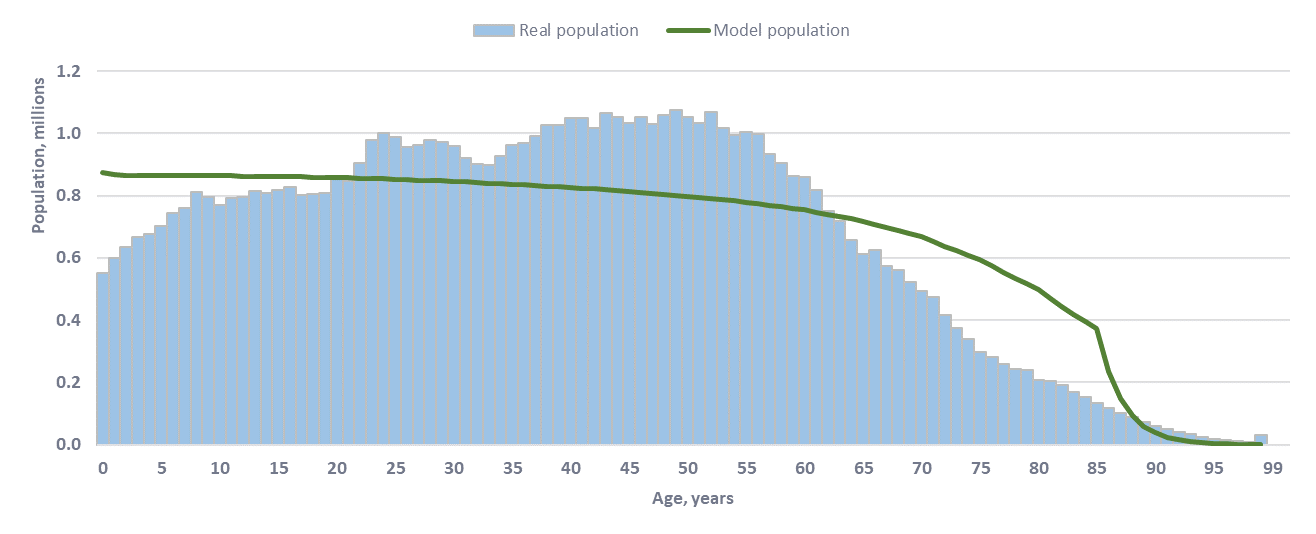


The annual probability of dying presented above was also used to simulate **life expectancy** by single year of age (see Fig G). These values were then used to estimate the number of years of life and productivity lost to premature death.

##### Fig G. Thailand, life expectancy of the model population, by age. The life expectancy at birth was estimated to be 76 years. This value was fairly close to the life expectancy of 77.7 years reported for Thailand by the World Health Organization [14].


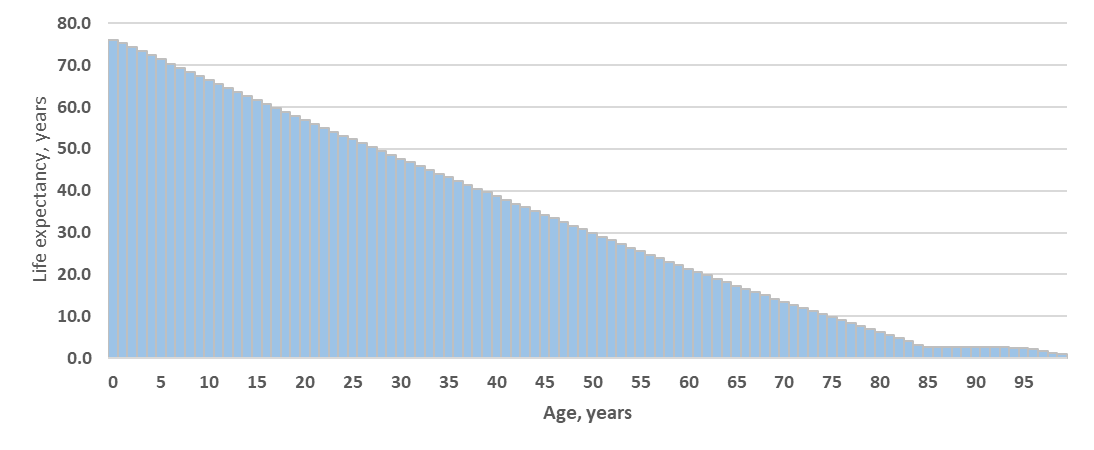


## Vector population

Modeling of the **vector population size** is described in the “Modeling of vector population” section (Section 1.2) in S1 Material. The study by Bartley et al. (2002) for Thailand [16] and its adaptation by Coudeville and Garnett (2012) [17] for Southern Vietnam assumed that the average annual **vector-to-host ratio** was equal to 2. Following the same assumption, the size of the vector population was assumed to vary on average proportionally to the size of the host population,^[[1]](#footnote-1)^ consistent with the predator-prey paradigm. Vector birth rates were adjusted to ensure that the vector population size would remain proportional to the size of the host population.

An important factor in the modeling of the vector population is the **seasonality**, which was calibrated to match the observed incidence (see Section 1.2).

## Dengue epidemiology

### Dengue incidence

The incidence of dengue in Thailand used to inform the model is described in Section 1.1.

### Severity structure

To estimate the proportions of infections by severity, the model required inputs on the probability of being symptomatic, hospitalized, or severe following infection, as well as the probability of dengue-related death.

Table 6 summarizes the probabilities that were used in the adaptation for Thailand. Sources and calculations for each value in the table are described further in this section.

###### Table 6. Thailand, severity structure inputs.

| **Probability** | **Primary**  **infection** | **Secondary**  **infection** | **Postsecondary infection** |
| --- | --- | --- | --- |
| Symptomatic disease, with an infection | 30% | 60% | 10% |
| Hospitalization, with symptomatic disease | 15.9% | 30.0% | 7.5% |
| Severe disease, with symptomatic disease | 6.3% | 11.8% | 2.9% |
| Dengue-caused death, with severe disease | 0.259% | 0.259% | 0.259% |

**The probabilities of symptomatic disease with dengue infection were set to 30% for primary infections, 60% for secondary infections, and 10% for postsecondary infections, as the values commonly used in the models** included in Flasche et al*.* [18].

The country-specific **probability of being hospitalized with a symptomatic case** is challenging to estimate, as it reflects both the severity of disease and local healthcare practices; the latter may vary considerably from country to country. To overcome this limitation, the probability of hospitalization was manually adjusted by comparing the output of model calibration and the average annual number of hospitalizations observed in Thailand, which was used as the calibration target.

To estimate the **average annual number of hospitalizations**, the number of reported cases and the proportions of those that were hospitalized were derived from the National Disease Surveillance (Report 506) and from the Annual Epidemiology Surveillance Report, respectively [13,19]. These data allowed for calculating the number of reported hospitalizations at each year (only the period 2013–2019 was used, as the proportion of hospitalized cases was not available for the years 2011–2012 or the year 2020). To account for underreporting, this number was then adjusted using the **expansion factor of 2.94** derived from Wichmann et al. (2011) [4] specifically for cases treated in an inpatient setting. The average annual number of hospitalizations (accounting for underreporting) was estimated at 177,067 (see Table 7 for the details of the calculations).

###### Table 7. Thailand, annual number of hospitalized cases (with and without expansion), by year.

| **Year** | **Number reported cases (DF + DHF + DSS)** | **% hospitalized** | **Number of reported hospitalizations^a^** | **Number of hospitalizations, accounting for underreporting^b^** |
| --- | --- | --- | --- | --- |
| 2013 | 154,444 | 63.21 | 97,624 | 287,015 |
| 2014 | 41,082 | 65.65 | 26,970 | 79,293 |
| 2015 | 144,952 | 64.22 | 93,088 | 273,679 |
| 2016 | 63,931 | 61.85 | 39,541 | 116,251 |
| 2017 | 53,961 | 62.70 | 33,834 | 99,471 |
| 2018 | 87,212 | 62.37 | 54,394 | 159,919 |
| 2019 | 131,157 | 58.05 | 76,137 | 223,842 |
|  |  |  | **Average** | **177,067** |
| ^a^Number of reported cases × % hospitalized.  ^b^Number of hospitalizations × expansion factor (2.94).  DF, dengue fever; DHF, dengue hemorrhagic fever; DSS, dengue septic shock syndrome. | | | | |

To find the probability of being hospitalized that would allow for the model to predict 177,067 hospitalizations per year, the model was first calibrated using the probability of hospitalization derived from Flasche et al. [18]. These probabilities were equal to 11.1%, 20.9%, and 5.2% for primary, secondary, and postsecondary infections, respectively. However, with these values, the number of hospitalizations predicted by the model was considerably lower than the target number.

To bring the model output closer to the target value, the probability of hospitalization was back-calculated while preserving the same ratio between each type of infection (primary, secondary, and postsecondary), reported in Table 8.

###### Table 8. Thailand, probability of hospitalization with symptomatic dengue (before and after adjustment).

| **Probability of hospitalization with symptomatic dengue** | **Primary**  **infection** | **Secondary**  **infection** | **Postsecondary**  **infection** |
| --- | --- | --- | --- |
| Original values (as in UWA and UND) | 11.1% | 20.9% | 5.2% |
| Ratio to primary infection | 1.00 | 1.88 | 0.47 |
| Revised values | 15.9% | 30.0% | 7.5% |
| UND, University of Notre Dame; UWA, University of Western Australia. | | | |

The **probability of severe disease with symptomatic dengue** was calculated using the data from Sabchareon et al. (2012) [5]. This study reported the occurrence of 40 cases of dengue hemorrhagic fever (DHF) among 338 total dengue cases, which allowed for estimating the probability of severe dengue at 11.8% (see Table 9 for details of the calculation). However, this estimate could only be applied to secondary infections, in line with the definitions used by authors.

###### Table 9. Thailand, % DHF among dengue cases.

| **Parameter** | **Value** |
| --- | --- |
| Secondary DHF | 40 |
| Secondary DF | 126 |
| Secondary UF | 172 |
| Secondary DHF + DF + UF | 338 |
| % DHF out of DHF + DF + UF | 11.8 |
| DF, dengue fever; DHF, dengue hemorrhagic fever; UF, undifferentiated fever.  Source: Sabchareon et al. (2012) [5]. | |

The probability of hospitalization and probability of severe disease with symptomatic secondary infection being equal to 30% and 11.8%, respectively, implied that 39% of hospitalized cases were severe (assuming that all severe cases are hospitalized). In the absence of data for primary and postsecondary infections, the same proportion was applied to the respective probabilities of being hospitalized, resulting in the probability of severe dengue with symptomatic disease of 6.3%, 11.8%, and 2.9%, for primary, secondary, and postsecondary infections, respectively.

Finally, to estimate **the probability of dengue-caused death with severe disease** in Thailand, the following data from the National Disease Surveillance (Report 506) [20] were used:

- The number of DHF and dengue shock syndrome (DSS) cases, reflecting the total number of severe cases; and
- The number of dengue fever (DF), DHF, and DSS deaths, reflecting the total number of deaths.

The average ratio of deaths to severe cases was estimated at 0.259% (Table 10). This value was applied uniformly to primary, secondary, and postsecondary infections.

###### Table 10. Thailand, probability of death with severe dengue.

| **Year** | **Number of severe cases (A)** | | | **Number of deaths (B)** | | | | **% death among severe cases (B/A)** |
| --- | --- | --- | --- | --- | --- | --- | --- | --- |
|  | **DHF** | **DSS** | **Total** | **DHF death** | **DSS death** | **DF death** | **Total** |  |
| 2011 | 38,639 | 1,402 | 40,041 | 16 | 47 | 0 | 63 | 0.2 |
| 2012 | 37,675 | 1,341 | 39,016 | 26 | 55 | 0 | 81 | 0.2 |
| 2013 | 64,645 | 2,297 | 66,942 | 32 | 98 | 6 | 136 | 0.2 |
| 2014 | 17,354 | 558 | 17,912 | 6 | 43 | 0 | 49 | 0.3 |
| 2015 | 56,908 | 1,391 | 58,299 | 36 | 103 | 9 | 148 | 0.3 |
| 2016 | 24,748 | 717 | 25,465 | 11 | 48 | 5 | 64 | 0.3 |
| 2017 | 21,508 | 610 | 22,118 | 21 | 46 | 4 | 71 | 0.3 |
| 2018 | 31,177 | 959 | 32,136 | 31 | 78 | 6 | 115 | 0.4 |
| 2019 | 42,185 | 1,106 | 43,291 | 29 | 105 | 8 | 142 | 0.3 |
| 2020 | 20,908 | 552 | 21,460 | 18 | 27 | 6 | 51 | 0.2 |
|  |  |  |  |  |  |  | **Average** | **0.259** |
| DF, dengue fever; DHF, dengue hemorrhagic fever; DSS, dengue shock syndrome. | | | | | | | | |

There were 2 limitations to this estimation. First, due to a lack of data, no expansion factors were applied to either severe or fatal cases to account for potential underreporting. This was equivalent to assuming that severe and fatal cases would be equally underreported, which may not be the case.

Second, the model assumed that a dengue-caused death was only possible with a severe case. However, the data reported a small number of deaths due to DF (see the column “DF death” in Table 10), which was not included in the estimation of severe cases. Given the very small values, the underlying model assumptions were not revised.

### Dengue natural history

Natural history parameters, such as the duration of cross-protection, of the incubation period (in both hosts and vectors) and of the viremia period in hosts were informed by the published models of dengue transmission [16,17,21-28]. The values are summarized in Section 3.

**Symptomatic infections were assumed to be 2 times more transmissible than asymptomatic infections,** based on the precedent in Ferguson et al. (2016) [29] and Rodriguez-Barraquer et al*.* (2014) [30].

## Efficacy of TAK-003

### Clinical overview

DEN-301, also known as the TIDES trial, is a phase 3, double-blind, randomized, placebo-controlled trial with 2 parallel groups ([NCT02747927](https://clinicaltrials.gov/ct2/show/NCT02747927)) [31]. The trial was divided into 3 time periods (parts 1, 2, and 3) for surveillance of febrile illness with potential dengue etiology, as follows:

- Part 1 assessed the vaccine efficacy of 2 doses of TAK-003 for the prevention of virologically confirmed dengue (VCD) induced by any DENV serotype from 30 days after the secondary injection until 12 months after the second dose (the primary endpoint).
- Part 2 evaluated the vaccine efficacy by disease severity, serotype, and baseline serostatus from 30 days after the second dose until 18 months after the second dose (secondary endpoints).
- Part 3 continued to assess vaccine efficacy and long-term safety by monitoring participants for an additional 3 years, to complete a total of 4.5 years of follow-up (exploratory analyses) [32].

The target sample of 20,099 healthy children and adolescents aged between 4 and 16 years was randomized to receive either TAK-003 or placebo in a 2:1 ratio (13,401 administered TAK-003 and 6,698 administered sham placebo). Of these, 19,021 participants (94.6%) comprised the per-protocol set that was used for efficacy analyses (6,317 in the placebo group, 12,704 in the TAK-003 group). Randomization was stratified by region (Asia-Pacific and Latin America) and age range (children aged 4–5 years, 6–11 years, and 12–16 years) to ensure that each age range had the appropriate ratio of TAK-003 to placebo in each region.

### TAK-003 efficacy

The trial data showed that vaccine efficacy was variable by individual serotype [33]. The trial data were available stratified by year since vaccination, by serostatus at vaccination, and by serotype. However, the trial was not powered to detect differences in vaccine efficacy by serotype, and some of the case counts were low or 0, so the vaccine efficacy was not quantifiable at this level of granularity. Therefore, the overall vaccine efficacy against nonhospitalized symptomatic dengue was estimated in the post hoc analyses by year since vaccination and by serostatus at vaccination only.

The counts were estimated for both trial arms by year since vaccination (for years 1–4 after the second dose of vaccine) and separately for seronegative and seropositive individuals at vaccination (without stratification by infecting serotype). These counts were then used to calculate the vaccine efficacy based on the following equation:

${VE}_{s,y}=1-{RR}_{s,y}$,

where ${VE}_{s,y}$ is the vaccine efficacy against nonhospitalized dengue at year $y$ and for serostatus $s$ and ${RR}_{s,y}$ is the relative risk of nonhospitalized dengue in vaccine arm at year $y$ and for serostatus $s$.

This process resulted in a total of 8 estimates of vaccine efficacy, 1 for each year (1, 2, 3, and 4) and each serostatus (negative or positive), presented in Table 11.

###### Table 11. Point estimates for efficacy against nonhospitalized dengue, by serostatus at vaccination.

| **Seronegative at vaccination** | | | | **Seropositive at vaccination** | | | |
| --- | --- | --- | --- | --- | --- | --- | --- |
| **First year** | **Second year** | **Third year** | **Fourth year** | **First year** | **Second year** | **Third year** | **Fourth year** |
| 56% | 43% | 32% | 33% | 75% | 52% | 38% | 50% |

The efficacy was then extrapolated beyond the trial duration by fitting a power trend to the efficacy estimates, separately for each serostatus. The power trend line was fitted to serostatus-specific efficacy against nonhospitalized symptomatic dengue data using R function *lm* from R package *stats*, which fits linear models (Table 12).

###### Table 12. Vaccine efficacy against nonhospitalized dengue, by serostatus at vaccination.

| Year post vaccination | Efficacy in  seronegative recipients | Efficacy in  seropositive recipients |
| --- | --- | --- |
| 1 | 56.2% | 72.0% |
| 2 | 40.5% | 53.1% |
| 3 | 34.8% | 46.2% |
| 4 | 31.4% | 42.1% |
| 5 | 29.2% | 39.2% |
| 6 | 27.5% | 37.1% |
| 7 | 26.1% | 35.5% |
| 8 | 25.0% | 34.1% |
| 9 | 24.1% | 32.9% |
| 10 | 23.3% | 31.9% |
| 11 | 22.6% | 31.1% |
| 12 | 22.0% | 30.3% |
| 13 | 21.5% | 29.6% |
| 14 | 21.0% | 29.0% |
| 15 | 20.6% | 28.4% |
| 16 | 20.2% | 27.9% |
| 17 | 19.8% | 27.4% |
| 18 | 19.4% | 27.0% |
| 19 | 19.1% | 26.6% |
| 20 | 18.8% | 26.2% |
| 21 | 18.5% | 25.8% |
| 22 | 18.3% | 25.5% |
| 23 | 18.0% | 25.2% |
| 24 | 17.8% | 24.9% |
| 25 | 17.6% | 24.6% |
| 26 | 17.4% | 24.3% |
| 27 | 17.2% | 24.1% |
| 28 | 17.0% | 23.8% |
| 29 | 16.8% | 23.6% |
| 30 | 16.6% | 23.4% |

In the base case analyses, it was assumed that efficacy against nonhospitalized dengue infection was boosted by each asymptomatic or symptomatic breakthrough infection. In individuals who were seronegative at vaccination in the model, efficacy against nonhospitalized symptomatic dengue infections estimated for seronegative recipients was used (curve A in Fig H). Following the first breakthrough infection, vaccine efficacy in these individuals would be boosted to the level of protection of those who were seropositive at vaccination (curve B in Fig H). With the following breakthrough infections, the individuals were assumed to remain on the same efficacy curve (B in Fig H), but to be boosted back to its initial point (i.e., top of the curve, year 1). This process could be repeated up to 4 times, once for each serotype (see Fig F, panel A, in S1 Material), after which individuals were considered completely immune.

In individuals who were seropositive at vaccination, efficacy against nonhospitalized symptomatic dengue infections estimated for seropositive recipients was used (curve B in Fig H). Each breakthrough infection (regardless of symptoms or severity) was assumed to boost their level of protection back to the top of the curve (i.e., to the level of protection observed in the first year after the vaccination). The final efficacy curves stratified by the baseline serostatus and by infection post vaccination are summarized in in Fig H. The effect of repeated infections on the vaccination efficacy (natural boosting) is illustrated in Fig 3 of the manuscript.

##### Fig H. Vaccine efficacy against nonhospitalized dengue, by serostatus and infection post vaccination. Curve A shows efficacy for individuals seronegative at vaccination; curve B shows efficacy for individuals seropositive at vaccination (see Fig 2 in the manuscript). Note: Boosting includes a vaccination or a breakthrough infection.


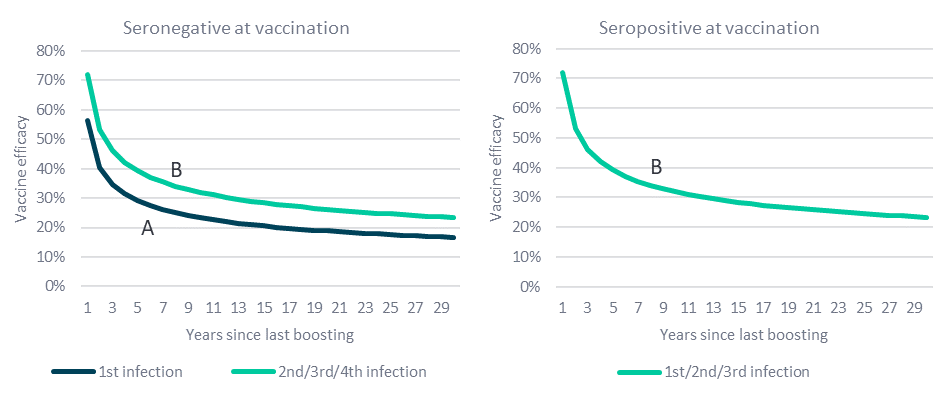


In the base case, it was assumed that vaccine efficacy against hospitalized dengue is constant, due to low degree of variability observed from year to year in the DEN-301 trial (see Table 13 below).

###### Table 13. Vaccine efficacy against hospitalized dengue, by year and overall.

| **Year since second dose** | **1** | **2** | **3** | **4** | **4.5** | **Overall** |
| --- | --- | --- | --- | --- | --- | --- |
| Vaccine efficacy against hospitalized VCD | 95.4% | 76.2% | 70.8% | 96.4% | 93.7% | 84.4% |
| VCD, virologically confirmed dengue. | | | | | | |

In the sensitivity analysis scenarios, both lower and upper confidence intervals (CIs) were tested and a scenario where the power curve was fitted to vaccine efficacy observed in the trial was implemented (S3 Material, Scenarios 8, 9, and 10).

Fig I presents the exponential curve fitted to vaccine efficacy against hospitalized dengue observed in the DEN-301 trial [34].

##### Fig I. Power curve fitted to vaccine efficacy against hospitalized dengue observed in the trial.


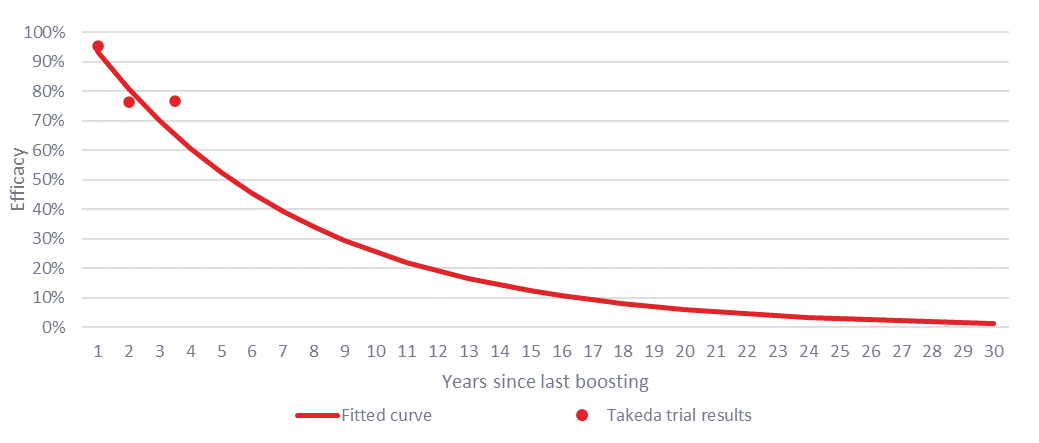


Existing evidence does not allow for precise estimation of the vaccine efficacy against asymptomatic infections due to the challenges in the definition and identification of asymptomatic infections. Thus, the approach for TAK-003 was based on the principle of the assumptions used in the modeling for CYD-TDV, supported by TAK-003–specific data.

**Vaccine efficacy against asymptomatic infection** was assumed to be half of the efficacy against symptomatic nonhospitalized infections (see “Determination of TAK-003 efficacy” section in the “Methods” of the manuscript).

One of the explored scenarios considered **vaccine efficacy stratified by both serostatus and serotype**. Due to low case counts at this level of granularity from the DEN-301 trial with low or zero case counts for some of the subgroups (Table 14).

The curves for efficacy against nonhospitalized dengue, by serotype and serostatus at vaccination, were estimated by adjusting the serostatus-specific vaccine efficacy curves (used in the base case analyses and described earlier in this section; see Fig H) with a multiplier. This multiplier was calculated as the ratio of the cumulative 48-month post–second dose serotype and serostatus-specific efficacy and corresponding cumulative 48-month post–second dose serostatus-specific efficacy. Results of the nonhospitalized vaccine efficacy against the serotypes DENV-3 (point estimate 14%; 95% CI –70% to 56%) and DENV-4 (–95%; 95% CI –819% to 58%) in the seronegative population were considered inconclusive, with the 95% CI crossing 0; therefore, the multiplier was set to 0 (i.e., zero efficacy against these 2 serotypes in seronegative participants). The cumulative nonhospitalized vaccine efficacies used in the calculation are presented in Table 14.

###### Table 14. Cumulative 48-month post–second dose vaccine efficacy against symptomatic nonhospitalized dengue infection, by serotype and serostatus at vaccination.

| **Parameter** | **Seronegative at vaccination** | | | | | **Seropositive at vaccination** | | | | |
| --- | --- | --- | --- | --- | --- | --- | --- | --- | --- | --- |
|  | **DENV-1** | **DENV-2** | **DENV-3** | **DENV-4** | **Overall** | **DENV-1** | **DENV-2** | **DENV-3** | **DENV-4** | **Overall** |
| Point estimate (95% CI) | 32%  (5% to 51%) | 84%  (64% to 93%) | 14%  (–70% to 56%) | -95%  (–819% to 58%) | 40%  (22% to 54%) | 49%  (34% to 61%) | 69%  (53% to 79%) | 45%  (25% to 59%) | 64%  (20% to 84%) | 53%  (44% to 60%) |
| Efficacy value used in the scenario of serostatus and serotype-specific efficacy | 32% | 73%^a^ | 0% | 0% | 40% | 49% | 73%^a^ | 45% | 64% | 53% |
| Point estimates coincide with values computed strictly from trial data; applied values coincide with values that were assumed after application of assumption described in text.  ^a^The point estimates for vaccine efficacy against symptomatic nonhospitalized DENV-2 infection was higher in seronegative participants at vaccination than in seropositive participants at vaccination; thus, the vaccine efficacy against DENV-2 infection was estimated without serostatus breakdown as a conservative estimate.  CI, confidence interval; DENV, dengue virus. | | | | | | | | | | |

The efficacy curves for seronegative and seropositive participants at vaccination stratified by virus serotype are reported in Fig J.

##### Fig J. Modified vaccine efficacy against nonhospitalized dengue, by serostatus at vaccination and virus serotype. Note: Curve for DENV-3 in seronegative participants at vaccination coincides with the curve for DENV-4 in seronegative participants at vaccination and is therefore not presented on the graph. DENV, dengue virus.


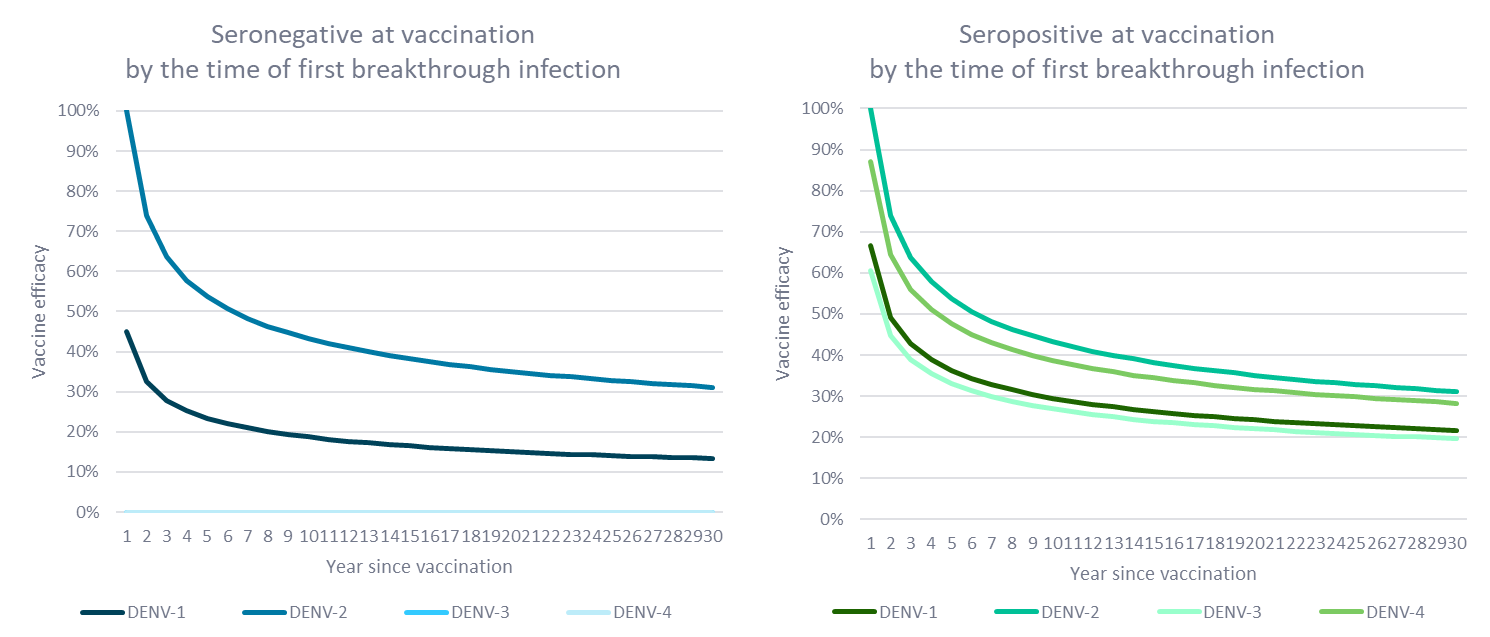


For hospitalized dengue, the stratification of vaccine efficacy by serostatus and serotype was performed as follows. For each serotype, the efficacy against hospitalized dengue was informed by the cumulative efficacy over 54 months. As in the base case where the efficacy was stratified by serostatus only, the efficacy stratified by serotype was assumed to remain stable over time. Data from the DEN-301 trial showed that vaccine efficacy against hospitalized dengue caused by DENV-1 and DENV-2 were numerically higher in participants who were seronegative at vaccination compared with participants who were seropositive at vaccination. Conservatively and for simplicity, the vaccine efficacy against DENV-1 and DENV-2 were not stratified by serostatus, and pooled estimates combining seronegative and seropositive participants were used.

The vaccine efficacy against DENV-3 hospitalized cases in seronegative participants at vaccination was inconclusive (point estimate of –79%; 95% CI –541% to 50%). The negative point estimate of vaccine efficacy against DENV-3 may have been confounded by overall low case counts and a high rate of hospitalization at Sri Lankan trial sites, likely to allow close monitoring. Analysis of the placebo group in the DEN-301 trial showed that Sri Lankan sites had a considerably higher background hospitalization rate (68.0%) among dengue cases compared with all the other countries that participated in the trial (for which rates ranged from 4.4% to 39.1%) [32]. If data for Sri Lanka were excluded from the estimation, the point estimate of the vaccine efficacy against hospitalized dengue caused by DENV-3 in participants seronegative at vaccination became positive, although was still not statistically significant (efficacy of 19%; 95% CI –239% to 81%). Based on this observation, the efficacy of TAK-003 against hospitalized dengue caused by DENV-3 in seronegative participants was assumed to be zero.

The same assumption of no efficacy was made for the hospitalized dengue caused by DENV-4 in seronegative participants, as only one case was observed in the placebo group with a vaccine efficacy point estimate of 100% in the timeframe of first dose to 54 months after the second dose.

For seropositive participants, the efficacy against hospitalized dengue caused by DENV-3 and DENV-4 was informed by the point estimates derived from DEN-301 (Table 15).

###### Table 15. Assumed vaccine efficacy against hospitalized dengue, by serostatus and dengue serotype.

| **Parameter** | **Seronegative at vaccination** | | | | **Seropositive at vaccination** | | | |
| --- | --- | --- | --- | --- | --- | --- | --- | --- |
|  | **DENV-1** | **DENV-2** | **DENV-3** | **DENV-4** | **DENV-1** | **DENV-2** | **DENV-3** | **DENV-4** |
| Point estimates (95% CI) | 82.6% (52% to 94%) | 100.0% (NA) | –79.1%  (–541% to 50%) | Lack of data | 71.7% (44% to 86%) | 97.9% (91% to 99%) | 71.4% (32% to 88%) | 100.0% (NA) |
| Efficacy value used in the scenario of serostatus and serotype-specific efficacy | 75.8%^a^ | 98.5%^a^ | 0% | 0% | 75.8%^a^ | 98.5%^a^ | 71.4% | 100.0% |
| Point estimates coincide with values computed strictly from trial data; applied values coincide with values that were assumed after application of assumption described in text.  ^a^The point estimates for vaccine efficacy against symptomatic nonhospitalized DENV-1 and DENV-2 infections were higher in seronegative participants at vaccination than in seropositive participants at vaccination; thus, the vaccine efficacies against DENV-1 and DENV-2 infection were estimated without serostatus breakdown as conservative estimates.  CI, confidence interval; DENV, dengue virus; NA, not analyzed. | | | | | | | | |

## Costs

This section provides a detailed description of the cost inputs used in the analyses. All costs were converted to 2021 values in US dollars.

### Costs of treatment

**Direct medical costs** were estimated based on data derived from Fitzpatrick et al. (2017) [35]. Expert opinion from a local physician was sought to fill in the data gaps as explained below. **Direct nonmedical costs** were estimated using the data from Suaya et al*.* (2009) [36]. The summary of inputs used in the base case analyses is provided in Table 16.

###### Table 16. Thailand, direct medical and nonmedical cost (per case), by case severity.

| **Case severity** | **Direct medical cost, US$** | **Direct nonmedical cost, US$** |
| --- | --- | --- |
| Nonhospitalized | 70.91 | 17.76 |
| Hospitalized mild | 657.76 | 80.56 |
| Hospitalized severe | 1,114.73 | 80.56 |

#### Direct medical costs

The direct medical costs shown in **Table 16** were estimated by multiplying the resource use specific to case severity by the corresponding unit costs. **Unit costs** were derived from the Fitzpatrick et al. study (2017) [35] and included ambulatory visits and hospital bed days; values are provided in Table 17.

###### Table 17. Thailand, direct medical costs, per unit.

| **Resource type** | **2013 US$** | **2021 US$** |
| --- | --- | --- |
| Ambulatory visit | 18.29 | 19.27 |
| Hospitalization bed day | 141.55 | 149.11 |
| Hospitalization bed day, specialist | 169.24 | 178.28 |
| Costs reported in Fitzpatrick et al. (2017) [35] were inflated to 2021 values. | | |

The estimates of the **resource use** associated with dengue cases of different severity were derived from the same study by Fitzpatrick et al. (2017) [35]. They were combined with an expert opinion by assuming that the number of hospital days for a fatal severe case would be 2 times higher than for a nonfatal severe case. The final estimates are provided in Table 18.

###### Table 18. Thailand, resource use (per case), by case severity (as in Fitzpatrick et al. [2017] [35]).

| **Resource type** | **Nonhospitalized** | **Hospitalized mild** | **Hospitalized severe nonfatal** | **Hospitalized severe fatal** |
| --- | --- | --- | --- | --- |
| Ambulatory visit | 3.68 | 4.42 | 4.42 | 4.42 |
| Hospitalization bed day | – | 3.84 | – | – |
| Hospitalization bed day, specialist | – | – | 5.76 | 11.52^a^ |
| ^a^Assumed to be 2 times higher than for a nonfatal severe case (based on the local expert opinion). | | | | |

The final values used in the model for direct medical costs (as shown in Table 16) were calculated by combining values from Table 17 and Table 18.

#### Direct nonmedical costs

The direct nonmedical costs were derived from the study by Suaya et al. (2019) [36]. This study reported the patient’s out-of-pocket payments for transportation, food, lodging, and other miscellaneous expenses associated with seeking medical care or visiting hospitalized family members (Table 19).

###### Table 19. Thailand, direct nonmedical cost (per case), by case severity (as in Suaya et al. [2009] [36]).

| **Treatment setting** | **2005 US$** | **2021 US$** |
| --- | --- | --- |
| Ambulatory | 13.36^a^ | 17.76 |
| Hospitalized | 60.59 | 80.56 |
| The original study provided the values in international dollars (values of 2005). They were converted to US dollars using the ratio of 3.07 reported in the publication. The resulting values were then inflated to 2021 values.  ^a^Value for Malaysia was used because the cohort for Thailand included only hospitalized children.  Source: Suaya et al. (2009) [36]. | | |

### Indirect costs

The indirect costs for each scenario included **2 main categories**:

- Cost of **school absenteeism**; and
- **Productivity loss** due to work absenteeism by either the patient or the patient’s caregiver.

The details of the estimation are provided in the next subsections.

#### Cost of school absenteeism

The cost of school absenteeism was estimated based on the cost of 1 day of schooling (equal to $2.53 after inflating the values provided in Kongsin et al. [2010] [37]), and on the number of school days lost to dengue cases of different severity.

The number of school days lost was derived from studies by Suaya et al. (2019) [36] (for nonhospitalized cases) and Tozan et al. (2017) [38] (for hospitalized cases). Because all the dengue-caused deaths in the model were assumed to occur in hospitalized severe cases, it appeared reasonable to assume that the number of school days lost to a fatal case would equal the length of hospital stay. The final values are summarized in Table 20.

School absenteeism was assumed to be only applicable to dengue cases occurring in children aged 6–15 years.

###### Table 20. Thailand, number of school days lost (per case), by case severity.

| **Case severity** | **Value** | **Source** |
| --- | --- | --- |
| Nonhospitalized | 4.2 | Suaya et al. (2009) [36] |
| Hospitalized mild | 6.3 | Tozan et al. (2017) [38] |
| Hospitalized severe | 8.6 | Tozan et al. (2017) [38], assumption^a^ |
| ^a^Number of school days lost was assumed equal to length of hospital stay for fatal cases (Table 18). | | |

#### Productivity loss due to work absenteeism

The productivity loss due to work absenteeism was estimated based on average daily wage and on the number of workdays lost (by patient or patient’s caregiver) to dengue cases of different severities. For fatal dengue cases, the productivity loss was estimated over the remaining life expectancy at the age of death.

The **average daily wage** in the base case analyses was $14.99; it was calculated based on average monthly wage in Thailand, as reported by Trading Economics for the period from October 2019 to January 2021 [39]. This daily wage was multiplied by an **employment rate** of 98.6% (based on an unemployment rate of 1.4% reported by the International Labour Organization) [40].

The **number of workdays lost by patient** was informed by studies by Suaya et al. (2019) [36] (for nonhospitalized cases) and Tozan et al. (2017) [38] (for hospitalized cases). Similarly for schooldays lost (see previous section), the number of workdays lost to a fatal case was assumed to be equal to length of hospital stay. The summary of final model inputs is provided in Table 21.

###### Table 21. Thailand, number of workdays lost by patient (per case), by case severity.

| **Case severity** | **Value** | **Source** |
| --- | --- | --- |
| Nonhospitalized | 6.6 | Suaya et al. (2009) [36] |
| Hospitalized mild | 6.6 | Tozan et al. (2017) [38] |
| Hospitalized severe | 7.6^a^ | Tozan et al. (2017) [38] |
| ^a^The number of workdays lost for a hospitalized severe case was estimated as a weighted average of the length of hospital stay for fatal and nonfatal cases (see Table 18). | | |

The estimates in Table 21 were only applied to dengue cases occurring in **individuals of working age** (assuming that in Thailand people start work at age 18 years [41] and retire at age 60 years [42]).

For the cases occurring in individuals younger than 18 years, productivity losses were assumed to be incurred by caregivers. The number of workdays lost by a caregiver was assumed to be the same as the number of school days lost by the sick child (Table 20). No productivity loss (for either patient or caregiver) was assumed for cases occurring after the age of 60 years.

The estimation also included the **lifetime of productivity lost to dengue-caused death.** For each fatal dengue case, the number of years of work lost was estimated by assuming that the deceased would have been employed between the ages of 18 and 60 years. The income lost at each year of work was estimated based on the average daily wage and employment rate described above, as well as on an average of 242 workdays a year [43].

### Cost of persistent dengue

The cost of persistent dengue was estimated based on the probability of its occurrence, duration, and associated monthly cost.

**Probability of occurrence** was informed by the literature review performed by Zeng et al. (2018), who estimated that 33.79% of dengue cases resulted in persistent symptoms [44]. Given the high estimate (1 in 3 cases resulting in persistent symptoms) and the heterogeneity of the studies included into Zeng’s review, other sources were used to better define the model parameters.

Teixeira et al. (2017) presented the results of a study that aimed to characterize persistent dengue symptoms in 113 individuals in Brazil [45]. The study concluded that progression to persistent symptoms was significantly associated with older age (relative risk of 2.1 in individuals aged 30–60 years, compared with individuals younger than 30 years). Based on these 2 sources, it was assumed that **persistent dengue occurred in 33.79% of symptomatic dengue cases in people aged ≥30 years**.

No specific estimates were found for the cost of persistent dengue. According to Teixeira et al. (2017) [45], the symptoms resolved over time, with no specific therapeutic interventions. The **monthly cost** of persistent dengue was assumed to be $9.48, based on the estimate for nonmedical cases (i.e., cases treated outside of professional healthcare) provided by Shepard et al. (2016) [46].

The **duration of persistent dengue** assumed in the model is discussed further in Section 2.6.

### Cost of vaccination

The cost of **vaccine administration** was assumed to be $2.3 per dose based on the data derived from Meeyai et al. (2015) [47]. The cost of **vaccine acquisition** in the base case analyses was assumed to be $30 per dose.

## Quality of life

The summary of the quality-of-life inputs used in this adaptation is provided in Table 22. The sources (and, when appropriate, intermediate calculations) for each item are described further in this section.

###### Table 22. Thailand, quality-of-life inputs.

| **Dengue severity** | **Disability duration** | **Disability weight** |
| --- | --- | --- |
| Mild dengue | 6 days | 0.197 |
| Severe dengue | 14 days | 0.545 |
| Persistent dengue | 2.69 months | 0.219 |

The duration of mild and severe dengue was assumed to be 6 and 14 days, respectively, and was taken from Stanaway et al. (2016) [48]. The duration of persistent dengue was assumed to be 2.69 months based on a systematic review done by Zeng et al. (2018) [44].

The disability weights of mild and severe dengue of 0.197 and 0.545, respectively, were taken from the WHO report, “The global burden of disease,” from 2004 [49]. The disability weight for persistent dengue of 0.219 was obtained from Salomon et al. (2015*)* [50].

# Summary of parameters

The parameters used in the model are summarized in Table 23.

###### Table 23. Summary of parameters used in the model.

| **Notation** | **Parameter** | **Value** | **Source** |
| --- | --- | --- | --- |
| **Parameters for host population** | | | |
| $N_{i}^{H}$ | Size of host population by age group | 66,486,458 | National Disease Surveillance (Report 506) [13] |
| $\frac{1}{\rho}$ | Duration of viremia in hosts (days) | 4.5 days | Entomology studies, modeling studies [16,17,21-23] |
| $\frac{1}{\xi_{H}}$ | Duration of latency period in hosts (days) | 5 days | Entomology studies, modeling studies [17,24,25] |
| $\mu_{i}$ | Host mortality rate | Table 5 | See Section 2.1 |
| $B^{H}$ | Number of new births | 872,649 | See Section 2.1 |
| **Parameters for host infection (without vaccination)** | | | |
| $\zeta_{CP}$ | Presence of cross-protection | Included | Modeling studies [16,26-28] |
| $\frac{1}{\varphi_{CP}}$ | Duration of cross-protection | 6 months | Modeling studies [16,26-28] |
| $\gamma_{CP}$ | Relative risk of a new infection during the cross-protection period:  $\gamma_{CP}=0$ indicates perfect cross-protection  $\gamma_{CP}=1$ indicates no cross-protection  $0<\gamma_{CP}<1$ indicates imperfect cross-protection | 0 | Assumption |
| $\theta^{klm}$ | Modifying parameter applied to the force of infection in the submodel without vaccination | Calculated, see “Infection process in host” section (Section 1.1.2) in S1 Material |  |
| $\lambda_{s, i}^{H}\left( t \right)$ | Force of infection due to serotype $s$ for a host aged $i$ at time $t$ | Calculated, see “Infection process in host” section (Section 1.1.2) in S1 Material |  |
| $\beta_{i}$ | Coefficient used to modify the force of infection for a host aged $i$ | Calibrated^a^ |  |
| $\psi_{sympt}$ | Infectiousness of a symptomatic infection relative to an asymptomatic infection | 2 | Modeling studies [30] |
| $\Psi_{s,i}^{klm}$ | Infectiousness of hosts currently infectious of serotype *s* and status for the other 3 serotypes being described by the indices *k*, *l*, and *m* in the cohort aged $i$ | Calculated, see “Modeling of vector population” (Section 1.2) section in S1 Material | See Section 1.2 |
| $p_{s,n,i}^{sympt\vert inf}$ | Probability of symptomatic cases with serotype $s$ of type $n$ in the cohort aged $i$ | Table 6 | Review of modeling studies |
| $p_{s,n,i}^{hosp\vert sympt}$ | Probability of a hospitalization given symptomatic disease with serotype $s$ of type $n$ in the cohort aged $i$ | Table 6 | See Section 2.3.2 |
| $p_{s,n,i}^{sev\vert hosp}$ | Probability of a severe case given hospitalized disease by infection type $n$ and serostatus $s$ in the cohort aged $i$ | Table 6 | See Section 2.3.2 |
| $p_{s,n,i}^{death\vert sev}$ | Probability of dengue-caused death given severe disease with serotype $s$ of type $n$ in the cohort aged $i$ | Table 6 | See Section 2.3.2 |
| **Host compartments (without vaccination)** | | | |
| $H_{i}^{jklm}\left( t \right)$ | Size of host compartment by age, for whom $j$*,* $k$*,* $l$, and $m$ indicate their status for DENV-1, DENV-2, DENV-3, and DENV-4, respectively, at time *t* | Calculated |  |
| **Host processes (without vaccination)** | | | |
| $D_{i}^{jklm}(t)$ | Movements due to the demographic processes (births, aging, and all-cause deaths) in the compartment $H_{i}^{jklm}$ at time $t$ | Calculated |  |
| $I_{s,i}^{jklm}\left( t \right)$ | Movements due to the infection process for serotype $s$ at time $t$, in the compartment $H_{i}^{jklm}$ at time $t$ | Calculated |  |
| **Parameters for vector population** | | | |
| $b$ | Average daily number of bites by an adult female vector | 0.7 per day | Modeling study [51] |
| $\mu_{V}$ | The daily death rate of a vector | Calculated as the inverse of the vector expectancy |  |
| $1/\mu_{V}$ | Vector life expectancy | 14 days | Entomology studies [24,52] |
| $R_{VH}$ | Vector-to-host ratio | 2 | Entomology studies [53-56] |
| $B_{V}\left( t \right)$ | Recruitment rate for female adult vectors at  time $t$ | Calculated as the inverse of the average life span |  |
| $\bar{B_{V}}$ | Average recruitment rate (without seasonality) | Calculated |  |
| $\zeta_{seas}$ | Binary variable indicating the presence or absence of seasonality | 1 | Assumption |
| $a_{seas}$ | Seasonality parameter, amplitude of sinusoidal function | 0.34 | See Section 1.2 |
| $b_{seas}$ | Seasonality parameter, horizontal shift of sinusoidal function | –0.51 | See Section 1.2 |
| **Parameters for vector infection** | | | |
| $\lambda_{s}^{V}(t)$ | Vector force of infection for the serotype *s* at time *t* | Calculated, see “Modeling of vector population” section (Section 1.2) in S1 Material |  |
| $\beta^{VH}$ | Probability of effective virus transmission from a vector infectious of serotype $s$ to a susceptible host given a bite | Calibrated | See Section 1.3 |
| $1/\xi_{V}$ | Extrinsic incubation period (duration of latency for vectors) | 10 days | Entomology studies, modeling studies [24,57,58] |
| **Vector compartments** | | | |
| $SV\left( t \right)$ | Compartment of susceptible vectors at time *t* | Calculated |  |
| ${EV}^{s}\left( t \right)$ | Compartment of vectors exposed to serotype $s$ at time *t* | Calculated |  |
| ${IV}^{s}\left( t \right)$ | Compartment of vectors infected with serotype $s$ at time *t* | Calculated |  |
| **Vaccine effects** | | | |
| $\theta_{v,s,i}^{klm}(t)$ | Modifying parameter applied to the force of infection in the submodel with vaccination | Calculated, see “Impact of vaccination on the acquisition of infection” section (Section 2.2.2) in S1 Material |  |
| $\psi_{asympt}^{vac}$ | Infectiousness of an asymptomatic infection in a vaccinated individual relative to infection in an unvaccinated individual | 1 | Assumption |
| $\psi_{sympt}^{vac}$ | Infectiousness of a symptomatic infection relative to an asymptomatic infection in a vaccinated individual relative to infection in an unvaccinated individual | 1 | Assumption |
| $\Psi_{s,i}^{klmv}\left( t \right)$ | Relative infectiousness of the individuals in each compartment $H_{i}^{3klmv}$, as determined by the presence of symptoms, as well as the vaccination status, at time $t$ | Calculated |  |
| $\eta_{s}^{x,as}\left( y \right)$ | Efficacy against asymptomatic infection for serotype $s$, serostatus $x$ at vaccination, and time since vaccination$y$ | Half the efficacy against nonhospitalized infection | See Section 2.4.2 |
| $\eta_{s}^{x,snh}\left( y \right)$ | Efficacy against symptomatic nonhospitalized infection for serotype $s$, serostatus $x$ at vaccination, and time since vaccination$y$ |  | See Section 2.4.2 |
| $\eta_{s}^{x,sh}\left( y \right)$ | Efficacy against symptomatic hospitalized infection for serotype $s$, serostatus $x$ at vaccination, and time since vaccination$y$ | 84.4% | See Section 2.4.2 |
| $\eta_{s,e}^{x,as}\left( y \right)$ | Efficacy against asymptomatic infection for serotype $s$, serostatus $x$ at vaccination, and time since last episode/vaccination$y$ for an individual for whom that would be the *e*^th^ infection | Half the efficacy against nonhospitalized infection | See Section 2.4.2 |
| $\eta_{s,e}^{x,snh}\left( y \right)$ | Efficacy against symptomatic nonhospitalized infection for serotype $s$, serostatus $x$ at vaccination, and time since last episode/vaccination$y$ for an individual for whom that would be the *e*^th^ infection |  | See Section 2.4.2 |
| $\eta_{s,e}^{x,sh}\left( y \right)$ | Efficacy against symptomatic hospitalized infection for serotype $s$, serostatus $x$ at vaccination, and time since last episode/vaccination$y$ for an individual for whom that would be the *e*^th^ infection | 84.4% | See Section 2.4.2 |
| $\overline{\eta}_{s,i}^{x,as}\left( y \right)$ | Level of protection (efficacy/effectiveness) against asymptomatic dengue infection for an individual aged $i$, serotype $s$, at time $y$, serostatus $x$ at vaccination | Calculated |  |
| $\overline{\eta}_{s,i}^{x,snh}\left( y \right)$ | Level of protection (efficacy/effectiveness) against symptomatic nonhospitalized dengue infection for an individual aged $i$, serotype $s$, at time $t$, serostatus $x$ at vaccination | Calculated |  |
| $\overline{\eta}_{s,i}^{x,sh}\left( y \right)$ | Level of protection (efficacy/effectiveness) against symptomatic hospitalized dengue infection for an individual aged $i$, serotype $s$, at time $t$, serostatus $x$ at vaccination | Calculated |  |
| $\overline{\eta}_{s,i}^{x,inf}\left( y \right)$ | Level of protection (efficacy/effectiveness) against infection for an individual aged $i$, serotype $s$, at time $t$, serostatus $x$ at vaccination | Calculated |  |
| $\delta$ | Defines whether the symptomatic infections averted with vaccination become asymptomatic instead ($\delta=1$) or are prevented entirely ($\delta=0$) | Calculated |  |
| $\overline{p}_{s,i}^{x, as}(y)$ | Average weighted probability of asymptomatic infection with serotype $s$ in the fraction of the cohort aged $i$ who had serostatus $x$ at vaccination | Calculated |  |
| $\overline{p}_{s,i}^{x,snh}(y)$ | Average weighted probability of symptomatic nonhospitalized infection with serotype $s$ in the fraction of the cohort aged $i$ who had serostatus $x$ at vaccination | Calculated |  |
| $\overline{p}_{s,i}^{x,sh}(y)$ | Average weighted probability of symptomatic hospitalized infection with serotype $s$ in the fraction of the cohort aged $i$ who had serostatus $x$ at vaccination | Calculated |  |
| **Host compartments (with vaccination)** | | | |
| $H_{i}^{jklmv}\left( t \right)$ | Size of host compartment by age, for whom $j$*,* $k$*,* $l$, and $m$ indicate their status for DENV-1, DENV-2, DENV-3, and DENV-4, respectively, with vaccination status $v$, at time *t* | Calculated |  |
| **Host processes (with vaccination)** | | | |
| $D_{i}^{jklmv}(t)$ | Movements due to the demographic processes (births, aging, and all-cause deaths) in the compartment $H_{i}^{jklm}$, with vaccination status $v$, at time $t$ | Calculated |  |
| $I_{s,i}^{jklmv}\left( t \right)$ | Movements due to the infection process for serotype $s$ at time $t$, in the compartment $H_{i}^{jklm}$, with vaccination status $v$, at time $t$ | Calculated |  |
| $V_{i}^{jklmv}\left( t \right)$ | Movements due to the vaccination in the compartment $H_{i}^{jklmv}$ at time $t$ | Calculated |  |
| **Parameters that define vaccination strategy** | | | |
| $\chi_{i}(t)$ | Vaccination coverage for age cohort $i$ at time $t$ | See “Vaccination strategies” section in the “Methods” of the main manuscript body | Assumption |
| **Parameters used in calibration** | | | |
| $EF$ | Expansion factor | Model input |  |
| $n_{I}^{rep}$ | Rate of reported incidence of age group $I$ | Table 3 | See Section 1.1 |
| $n_{I}^{obs}$ | Rate of observed incidence of age group $I$ | Calculated |  |
| $n_{m}^{obs}$ | Rate of observed incidence in month $m$ | Calculated |  |
| ${sp}_{i}^{p}\left( t \right)$ | Seroprevalence for hosts aged $i$, at time $t$, with seroprevalence status $p$ | Calculated |  |
| **DALYs^b^** | | | |
| $N$ | Number of cases | Calculated |  |
| $M$ | Number of deaths | Calculated |  |
| $DW$ | Disability weight equal to 0 for perfect health and 1 for death | Calculated |  |
| $A$ | Age at disease onset or at death | Calculated |  |
| $L$ | Duration of the disease or remaining life expectancy at the age of death | Fig G | See Section 2.1 |
| $C$*,* $\beta$ | Constants used in a standard age-weighting formula | 0, 0 | Assumption that DALYs are not weighted by age (see “Estimation of quality-of-life outcomes” section (Section 3) in S1 Material) |
| $r$ | Discount rate | 3% | Assumption |
| $x$ | Concerned age | Calculated |  |
| $a$ | Age to which the burden is assigned | Model input |  |
| **Costs** | | | |
| $n_{vac}$ | Number of administered vaccination doses | 2 | Assumption |
| $c_{vac}$ | Cost of 1 dose | $30 | Assumption |
| $c_{vac\_adm}$ | Cost of vaccine administration | $2.3 per dose | [47] |
| $c_{dir\_med}^{sympt\_non\_hosp}\left( i \right)$ | Direct medical costs per dengue case that is symptomatic nonhospitalized, in hosts aged $i$ | $70.91 | [35] |
| $c_{dir\_med}^{hosp\_mild}\left( i \right)$ | Direct medical costs per dengue case that is symptomatic hospitalized mild, in hosts aged $i$ | $657.76 | [35] |
| $c_{dir\_med}^{hosp\_severe}\left( i \right)$ | Direct medical costs per dengue case that is symptomatic hospitalized severe, in hosts aged $i$ | $1,114.73 | [35] |
| $c_{dir\_non\_med}^{sympt\_non\_hosp}\left( i \right)$ | Direct nonmedical costs per dengue case that is symptomatic nonhospitalized, in hosts aged $i$ | $17.76 | [36] |
| $c_{dir\_non\_med}^{hosp\_mild}\left( i \right)$ | Direct nonmedical costs per dengue case that is symptomatic hospitalized mild, in hosts aged $i$ | $80.56 | [36] |
| $c_{dir\_non\_med}^{hosp\_severe}\left( i \right)$ | Direct nonmedical costs per dengue case that is symptomatic hospitalized severe, in hosts aged $i$ | $80.56 | [36] |
| ${dur}_{pers}$ | Duration of persistent dengue | 2.69 months | [44] |
| $c_{pers}$ | Monthly cost of persistent dengue | $9.48 | [46] |
| $p_{i}^{pers}$ | Proportion of cases resulting in persistent dengue in hosts aged $i$ | 33.79% in people aged ≥30 years | [44,45] |
| ${wd}_{patient}^{sympt\_non\_hosp}\left( i \right)$ | Number of workdays lost by patient per dengue case that is symptomatic nonhospitalized, in a host aged $i$ | 6.6 | [36] |
| ${wd}_{patient}^{hosp\_mild}\left( i \right)$ | Number of workdays lost by patient per dengue case that is symptomatic hospitalized mild, in a host aged $i$ | 6.6 | [35] |
| ${wd}_{patient}^{hosp\_severe}\left( i \right)$ | Number of workdays lost by patient per dengue case that is symptomatic hospitalized severe, in a host aged $i$ | 7.61 | [35] |
| ${wd}_{caregiver}^{sympt\_non\_hosp}\left( i \right)$ | Number of workdays lost by caregiver per dengue case that is symptomatic nonhospitalized, in a host aged $i$ | 4.2 | [36] |
| ${wd}_{caregiver}^{hosp\_mild}\left( i \right)$ | Number of workdays lost by caregiver per dengue case that is symptomatic hospitalized mild, in a host aged $i$ | 6.3 | [35] |
| ${wd}_{caregiver}^{hosp\_severe}\left( i \right)$ | Number of workdays lost by caregiver per dengue case that is symptomatic hospitalized severe, in a host aged $i$ | 8.61 | [35] |
| $c_{daily wage}$ | Average daily wage | $14.99 | [39] |
| $p^{empl}$ | Employment rate | 98.6% | [40] |
| $c_{prod}^{fatal}\left( i \right)$ | Cost of mortality-related productivity, in a host aged $i$ | Calculated |  |
| ${wd}^{annual}$ | Number of workdays in the year | 242 | [43] |
| ${wy}^{left}\left( i \right)$ | Number of lost work years from the age of patient death to the retirement age for a host aged $i$ that would be left in the case of no fatality of the dengue case | Calculated |  |
| $i^{we}$ | End of working age | 60 | [42] |
| $i^{ws}$ | Start of working age | 18 | [41] |
| $L\left( i \right)$ | Life expectancy for a host aged $i$ | Fig G | See Section 2.1 |
| ^a^The parameter $\beta_{i}$ is calibrated using a simpler static model (see Section 1.3).  ^b^The notation is as in the original publication (see “Estimation of quality-of-life outcomes” section [Section 3] in S1 Material).  DALY, disability-adjusted life-year. | | | |

# References

**1.** Lu X, Bambrick H, Pongsumpun P, Dhewantara PW, Toan DTT, Hu W. Dengue outbreaks in the COVID-19 era: Alarm raised for Asia. PLoS Negl Trop Dis. 2021; 15(10):e0009778. <https://doi.org/10.1371/journal.pntd.0009778> PMID: 34624031; PubMed Central PMCID: PMC8500420.

**2.** Undurraga EA, Halasa YA, Shepard DS. Use of expansion factors to estimate the burden of dengue in Southeast Asia: a systematic analysis. PLoS Negl Trop Dis. 2013; 7(2):e2056. <https://doi.org/10.1371/journal.pntd.0002056> PMID: 23437407; PubMed Central PMCID: PMC3578761.

**3.** Limkittikul K, Brett J, L'Azou M. Epidemiological trends of dengue disease in Thailand (2000-2011): a systematic literature review. PLoS Negl Trop Dis. 2014; 8(11):e3241. <https://doi.org/10.1371/journal.pntd.0003241> PMID: 25375766; PubMed Central PMCID: PMC4222696.

**4.** Wichmann O, Yoon IK, Vong S, Limkittikul K, Gibbons RV, Mammen MP, et al. Dengue in Thailand and Cambodia: An assessment of the degree of underrecognized disease burden based on reported cases. PLoS Negl Trop Dis. 2011; 5(3):e996. <https://doi.org/10.1371/journal.pntd.0000996> PMID: 21468308; PubMed Central PMCID: PMC3066139.

**5.** Sabchareon A, Sirivichayakul C, Limkittikul K, Chanthavanich P, Suvannadabba S, Jiwariyavej V, et al. Dengue infection in children in Ratchaburi, Thailand: A cohort study. I. Epidemiology of symptomatic acute dengue infection in children, 2006-2009. PLoS Negl Trop Dis. 2012; 6(7):e1732. <https://doi.org/10.1371/journal.pntd.0001732> PMID: 22860141; PubMed Central PMCID: PMC3409110.

**6.** Nealon J, Taurel AF, Capeding MR, Tran NH, Hadinegoro SR, Chotpitayasunondh T, et al. Symptomatic dengue disease in five Southeast Asian countries: epidemiological evidence from a dengue vaccine trial. PLoS Negl Trop Dis. 2016; 10(8):e0004918. <https://doi.org/10.1371/journal.pntd.0004918> PMID: 27532617; PubMed Central PMCID: PMC4988713.

**7.** Ministry of Public Health (Thailand). National Disease Surveillance (Report 506) 2021 [cited 2021 July 16]. Available from: <http://doe.moph.go.th/surdata>.

**8.** National Statistic Office Thailand [cited 2021 July 16]. Available from: <http://statbbi.nso.go.th/staticreport/page/sector/th/01.aspx>.

**9.** Steffensen J. Interpolation. 2nd ed. Mineola, NY: Dover Publications; 2006.

**10.** Mhaskar H, Pai D. Fundamentals of Approximation Theory: CRC Press; 2000.

**11.** Moré JJ. The Levenberg-Marquardt algorithm: Implementation and theory. Numerical Analysis Lecture Notes in Mathematics, vol 630. Berlin, Heidelberg: Springer; 1978.

**12.** Guzzetta G, Poletti P, Del Fava E, Ajelli M, Scalia Tomba GP, Merler S, et al. Hope-Simpson's progressive immunity hypothesis as a possible explanation for herpes zoster incidence data. Am J Epidemiol. 2013; 177(10):1134-42. <https://doi.org/10.1093/aje/kws370> PMID: 23548754.

**13.** Global Health Data Exchange (GHDx). Thailand National Disease Surveillance Report 506 Dengue Fever, Dengue Hemorrhagic Fever, and Dengue Shock Syndrome 2018 [updated 2020 March 27; cited 2023 January 16]. Available from: <https://ghdx.healthdata.org/record/thailand-national-disease-surveillance-report-506-dengue-fever-dengue-hemorrhagic-fever-and-0>.

**14.** World Health Organization. Global Health Observatory data repository: Life tables by country - Thailand 2019 [cited 2023 January 16]. Available from: <https://apps.who.int/gho/data/view.main.61640?lang=en>.

**15.** National Statistical Office. Number of population from registration classified by age, sex, region and province, year 2020. Thailand [cited 2023 January 16]. Available from: <http://statbbi.nso.go.th/staticreport/page/sector/en/01.aspx>.

**16.** Bartley LM, Donnelly CA, Garnett GP. The seasonal pattern of dengue in endemic areas: Mathematical models of mechanisms. Trans R Soc Trop Med Hyg. 2002; 96(4):387-97. <https://doi.org/10.1016/S0035-9203(02)90371-8> PMID: 12497975.

**17.** Coudeville L, Garnett GP. Transmission dynamics of the four dengue serotypes in southern Vietnam and the potential impact of vaccination. Plos One. 2012; 7(12):e51244. <https://doi.org/10.1371/journal.pone.0051244> PMID: 23251466; PubMed Central PMCID: PMC3519629.

**18.** Flasche S, Jit M, Rodríguez-Barraquer I, Coudeville L, Recker M, Koelle K, et al. The long-term safety, public health impact, and cost-effectiveness of routine vaccination with a recombinant, live-attenuated dengue vaccine (Dengvaxia): A model comparison study. PLOS Medicine. 2016; 13(11):e1002181. <https://doi.org/10.1371/journal.pmed.1002181> PMID: 27898668; PubMed Central PMCID: PMC5127514.

**19.** Bureau of Epidemiology. Annual epidemiology surveillance report [cited 2024 January 16]. Available from: <https://apps-doe.moph.go.th/boeeng/annual.php>.

**20.** Bureau of Epidemiology. Annual epidemiology surveillance report 2021 [cited 2024 March 20]. Available from: <https://apps-doe.moph.go.th/boeeng/annual.php>.

**21.** Chao DL, Halstead SB, Halloran ME, Longini IM, Jr. Controlling dengue with vaccines in Thailand. PLoS Negl Trop Dis. 2012; 6(10):e1876. <https://doi.org/10.1371/journal.pntd.0001876> PMID: 23145197; PubMed Central PMCID: PMC3493390.

**22.** Gubler DJ, Suharyono W, Tan R, Abidin M, Sie A. Viraemia in patients with naturally acquired dengue infection. Bull World Health Organ. 1981; 59(4):623-30. PMID: 6976230; PubMed Central PMCID: PMC2396101.

**23.** Vaughn DW, Green S, Kalayanarooj S, Innis BL, Nimmannitya S, Suntayakorn S, et al. Dengue viremia titer, antibody response pattern, and virus serotype correlate with disease severity. J Infect Dis. 2000; 181(1):2-9. <https://doi.org/10.1086/315215> PMID: 10608744.

**24.** Chikaki E, Ishikawa H. A dengue transmission model in Thailand considering sequential infections with all four serotypes. J Infect Dev Ctries. 2009; 3(9):711-22. <https://doi.org/10.3855/jidc.616> PMID: 19858573.

**25.** Wearing HJ, Rohani P. Ecological and immunological determinants of dengue epidemics. Proc Natl Acad Sci U S A. 2006; 103(31):11802-7. <https://doi.org/10.1073/pnas.0602960103> PMID: 16868086; PubMed Central PMCID: PMC1544250.

**26.** Karl S, Halder N, Kelso JK, Ritchie SA, Milne GJ. A spatial simulation model for dengue virus infection in urban areas. BMC Infect Dis. 2014; 14(1):447. <https://doi.org/10.1186/1471-2334-14-447> PMID: 25139524; PubMed Central PMCID: PMC4152583.

**27.** Aguiar M, Kooi B, Stollenwerk N. Epidemiology of dengue fever: a model with temporary cross-immunity and possible secondary infection shows bifurcations and chaotic behaviour in wide parameter regions. Math Model Nat Phenom. 2008; 3(4):48-70. <https://doi.org/10.1051/mmnp:2008070>.

**28.** Knerer G, Currie CSM, Brailsford SC. Impact of combined vector-control and vaccination strategies on transmission dynamics of dengue fever: a model-based analysis. Health Care Manag Sci. 2015; 18(2):205-17. <https://doi.org/10.1007/s10729-013-9263-x> PMID: 24370922.

**29.** Ferguson NM, Rodriguez-Barraquer I, Dorigatti I, Mier YT-RL, Laydon DJ, Cummings DA. Benefits and risks of the Sanofi-Pasteur dengue vaccine: Modeling optimal deployment. Science. 2016; 353(6303):1033-6. <https://doi.org/10.1126/science.aaf9590> PMID: 27701113; PubMed Central PMCID: PMC5268127.

**30.** Rodriguez-Barraquer I, Mier-y-Teran-Romero L, Schwartz IB, Burke DS, Cummings DAT. Potential opportunities and perils of imperfect dengue vaccines. Vaccine. 2014; 32(4):514-20. <https://doi.org/10.1016/j.vaccine.2013.11.020> PMID: 24269318; PubMed Central PMCID: PMC4142437.

**31.** ClinicalTrials.gov. Efficacy, safety and immunogenicity of Takeda's Tetravalent Dengue Vaccine (TDV) in Healthy Children (TIDES) 2021 [cited 2024 March 19]. Available from: <https://classic.clinicaltrials.gov/ct2/show/NCT02747927>.

**32.** Tricou V, Yu D, Reynales H, Biswal S, Saez-Llorens X, Sirivichayakul C, et al. Long-term efficacy and safety of a tetravalent dengue vaccine (TAK-003): 4.5-year results from a phase 3, randomised, double-blind, placebo-controlled trial. Lancet Glob Health. 2024; 12(2):e257-e70. <https://doi.org/10.1016/S2214-109X(23)00522-3> PMID: 38245116.

**33.** Rivera L, Biswal S, Saez-Llorens X, Reynales H, Lopez-Medina E, Borja-Tabora C, et al. Three-year efficacy and safety of Takeda's dengue vaccine candidate (TAK-003). Clin Infect Dis. 2022; 75(1):107-17. <https://doi.org/10.1093/cid/ciab864> PMID: 34606595; PubMed Central PMCID: PMC9402653.

**34.** Plotkin SA. Correlates of protection induced by vaccination. Clin Vaccine Immunol. 2010; 17(7):1055-65. <https://doi.org/10.1128/cvi.00131-10> PMID: 20463105; PubMed Central PMCID: PMC2897268.

**35.** Fitzpatrick C, Haines A, Bangert M, Farlow A, Hemingway J, Velayudhan R. An economic evaluation of vector control in the age of a dengue vaccine. PLoS Negl Trop Dis. 2017; 11(8):e0005785. <https://doi.org/10.1371/journal.pntd.0005785> PMID: 28806786; PubMed Central PMCID: PMC5573582.

**36.** Suaya JA, Shepard DS, Siqueira JB, Martelli CT, Lum LC, Tan LH, et al. Cost of dengue cases in eight countries in the Americas and Asia: a prospective study. Am J Trop Med Hyg. 2009; 80(5):846-55. PMID: 19407136.

**37.** Kongsin S, Jiamton S, Suaya JA, Vasanawathana S, Sirisuvan P, Shepard DS. Cost of dengue in Thailand. WHO Regional Office for South-East Asia 2010 [cited 2024 September 17]. Available from: <https://iris.who.int/handle/10665/170969>.

**38.** Tozan Y, Ratanawong P, Sewe MO, Wilder-Smith A, Kittayapong P. Household costs of hospitalized dengue illness in semi-rural Thailand. PLoS Negl Trop Dis. 2017; 11(9):e0005961. <https://doi.org/10.1371/journal.pntd.0005961> PMID: 28937986; PubMed Central PMCID: PMC5627959.

**39.** Trading Economics. Thailand average monthly wages 2021 [cited 2023 January 16]. Available from: <https://tradingeconomics.com/thailand/wages>.

**40.** The World Bank. Unemployment, total (% of total labor force) (modeled ILO estimate) - Thailand 2022 [cited 2023 January 16]. Available from: <https://data.worldbank.org/indicator/SL.UEM.TOTL.ZS?locations=TH>.

**41.** Panwa Accounting Auditing & Business Services Bangkok Phuket - Thailand. Labor rules and regulations in Thailand [cited 2023 January 16]. Available from: <http://www.panwagroup.net/business/index2.html>.

**42.** Tilleke & Gibbins. New retirement regime for Thai private sector employees 2018 [cited 2023 January 16]. Available from: <https://www.tilleke.com/insights/new-retirement-regime-thai-private-sector-employees/>.

**43.** ExcelNotes. Working days in Thailand in 2022 [cited 2023 January 16]. Available from: <https://excelnotes.com/working-days-thailand-2022/>.

**44.** Zeng W, Halasa-Rappel YA, Durand L, Coudeville L, Shepard DS. Impact of a nonfatal dengue episode on disability-adjusted life years: A systematic analysis. Am J Trop Med Hyg. 2018; 99(6):1458-65. <https://doi.org/10.4269/ajtmh.18-0309> PMID: 30277202; PubMed Central PMCID: PMC6283510.

**45.** Teixeira LAS, Nogueira F, Nascentes GAN. Prospective study of patients with persistent symptoms of dengue in Brazil. Rev Inst Med Trop Sao Paulo. 2017; 59:e65. <https://doi.org/10.1590/s1678-9946201759065> PMID: 28876417; PubMed Central PMCID: PMC5587034.

**46.** Shepard DS, Undurraga EA, Halasa YA, Stanaway JD. The global economic burden of dengue: A systematic analysis. Lancet Infect Dis. 2016; 16(8):935-41. <https://doi.org/10.1016/S1473-3099(16)00146-8> PMID: 27091092.

**47.** Meeyai A, Praditsitthikorn N, Kotirum S, Kulpeng W, Putthasri W, Cooper BS, et al. Seasonal influenza vaccination for children in Thailand: A cost-effectiveness analysis. PLoS Med. 2015; 12(5):e1001829. <https://doi.org/10.1371/journal.pmed.1001829> PMID: 26011712; PubMed Central PMCID: PMC4444096.

**48.** Stanaway JD, Shepard DS, Undurraga EA, Halasa YA, Coffeng LE, Brady OJ, et al. The global burden of dengue: an analysis from the Global Burden of Disease Study 2013. Lancet Infect Dis. 2016; 16(6):712-23. <https://doi.org/10.1016/S1473-3099(16)00026-8> PMID: 26874619; PubMed Central PMCID: PMC5012511.

**49.** World Health Organization. The global burden of disease: 2004 update [cited 2023 January 16]. Available from: <https://www.who.int/publications/i/item/9789241563710>.

**50.** Salomon JA, Haagsma JA, Davis A, de Noordhout CM, Polinder S, Havelaar AH, et al. Disability weights for the Global Burden of Disease 2013 study. Lancet Glob Health. 2015; 3(11):e712-23. <https://doi.org/10.1016/s2214-109x(15)00069-8> PMID: 26475018.

**51.** Atkinson MP, Su Z, Alphey N, Alphey LS, Coleman PG, Wein LM. Analyzing the control of mosquito-borne diseases by a dominant lethal genetic system. Proc Natl Acad Sci U S A. 2007; 104(22):9540-5. <https://doi.org/10.1073/pnas.0610685104> PMID: 17519336; PubMed Central PMCID: PMC1876161

**52.** Cummings DA, Iamsirithaworn S, Lessler JT, McDermott A, Prasanthong R, Nisalak A, et al. The impact of the demographic transition on dengue in Thailand: insights from a statistical analysis and mathematical modeling. PLoS Med. 2009; 6(9):e1000139. <https://doi.org/10.1371/journal.pmed.1000139> PMID: 19721696; PubMed Central PMCID: PMC2726436.

**53.** Gubler DJ. Dengue and dengue hemorrhagic fever: its history and resurgence as a global public health problem. Dengue and dengue hemorrhagic fever. London, UK: CAB International; 1997.

**54.** Yasuno M, Tonn RJ. A study of biting habits of *Aedes aegypti* in Bangkok, Thailand. Bull World Health Organ. 1970; 43(2):319-25. PMID: 5312528; PubMed Central PMCID: PMC2427649.

**55.** Trpis M, Hausermann W. Dispersal and other population parameters of *Aedes aegypti* in an African village and their possible significance in epidemiology of vector-borne diseases. Am J Trop Med Hyg. 1986; 35(6):1263-79. <https://doi.org/10.4269/ajtmh.1986.35.1263> PMID: 3789275.

**56.** Jeffery JA, Thi Yen N, Nam VS, Nghia le T, Hoffmann AA, Kay BH, et al. Characterizing the *Aedes aegypti* population in a Vietnamese village in preparation for a *Wolbachia*-based mosquito control strategy to eliminate dengue. PLoS Negl Trop Dis. 2009; 3(11):e552. <https://doi.org/10.1371/journal.pntd.0000552> PMID: 19956588; PubMed Central PMCID: PMC2780318.

**57.** Alphey N, Alphey L, Bonsall MB. A model framework to estimate impact and cost of genetics-based sterile insect methods for dengue vector control. PLos One. 2011; 6(10):e25384. <https://doi.org/10.1371/journal.pone.0025384> PMID: 21998654; PubMed Central PMCID: PMC3187769.

**58.** Andraud M, Hens N, Beutels P. A simple periodic-forced model for dengue fitted to incidence data in Singapore. Math Biosci. 2013; 244(1):22-8. <https://doi.org/10.1016/j.mbs.2013.04.001> PMID: 23608712.

1. In the analyses, a stable host population was considered which translates into a constant average (annual seasonality put aside) vector population. [↑](#footnote-ref-1)
