# Supplementary material for: Vaccination strategies, public health impact and cost-effectiveness of dengue vaccine TAK-003: A modeling case study in Thailand
Source: PLoS Med. 2025 Jun 17;22(6):e1004631. doi: 10.1371/journal.pmed.1004631 (PMC12173404; doi:10.1371/journal.pmed.1004631)
Supplement: S3 File — (DOCX) [file pmed.1004631.s003.docx]

**Assessing the optimal vaccination strategies for dengue vaccine TAK-003, and its public health impact and
cost-effectiveness: A case study in Thailand**

S3 Material: Model results

This supplement (3) describes additional results not reported in the manuscript. There are 2 additional supplements:

- **S1 Material** describes the structure of the epidemiological model used in the study in detail.
- **S2 Material** describes the model fitting and validation process, the model inputs, and the source and rationale for the use of these values.

**Contents**

[1. Dengue incidence in the absence of vaccination (as simulated by the model) 4](#_Toc178262817)

[2. Cohort optimization 5](#_Toc178262818)

[3. Pragmatic scenario 12](#_Toc178262819)

[3.1. Impact of coverage rates 12](#_Toc178262820)

[3.2. Scenario analyses 13](#_Toc178262821)

[3.2.1. Description of scenarios 13](#_Toc178262822)

[3.2.2. Public health impact 15](#_Toc178262823)

[3.2.3. Cost-effectiveness 17](#_Toc178262824)

**List of figures**

[Fig A. Proportion of seropositive individuals, by age. 4](#_Toc178663985)

[Fig B. Panel A shows incidence rate of symptomatic dengue, by age. Panel B shows incidence rate of hospitalized dengue, by age. 4](#_Toc178663986)

[Fig C. Incidence rate of symptomatic dengue (before adjustment to underreporting), by calendar month. 5](#_Toc178663987)

**List of tables**

[Table 1. Proportion of infections avoided for vaccination strategy versus no vaccination (over 20 years). 5](#_Toc178262781)

[Table 2. Proportion of infections avoided for vaccination strategy versus no vaccination (over 10 years). 7](#_Toc178262782)

[Table 3. Incremental costs and effectiveness for vaccination strategy versus no vaccine at 20-year timeframe (discount rate 3%). Costs are reported in million US dollars. 8](#_Toc178262783)

[Table 4. Incremental costs and effectiveness for vaccination strategies versus no vaccine at 10-year timeframe (discount rate 3%). Costs are reported in million US dollars. 9](#_Toc178262784)

[Table 5. Cost-effective threshold pricing analysis at discount rate of 3% over 20 years for dominance or with WTP threshold of US $7,000 (1 × GDP)/DALY avoided. 10](#_Toc178262785)

[Table 6. Proportion of cases avoided with R11 over 20 years, by coverage rate. 12](#_Toc178262786)

[Table 7. List of scenarios tested for the comparison of routine vaccination versus no vaccination. 13](#_Toc178262787)

[Table 8. Number of infections over 20 years scenario analysis for R11. 15](#_Toc178262788)

[Table 9. Number (percentage) of infections avoided (over 20 years, undiscounted): scenario analysis for R11. 16](#_Toc178262789)

[Table 10. Cost-saving and effectiveness for routine vaccination strategy versus no vaccine at 20-year timeframe (discount rate 3%): scenario analyses. Costs are reported in million US dollars. 17](#_Toc178262790)

[Table 11. Threshold price analysis at discount rate of 3% over 20 years for dominance or with WTP threshold of US $7,000 (1 × GDP)/DALY avoided. 18](#_Toc178262791)

# Dengue incidence in the absence of vaccination (as simulated by the model)

The seroprevalence profile is reported in Fig A, the dengue incidence profiles are reported in Fig B, and the seasonality of incidence is reported in Fig C.

##### Fig A. Proportion of seropositive individuals, by age.


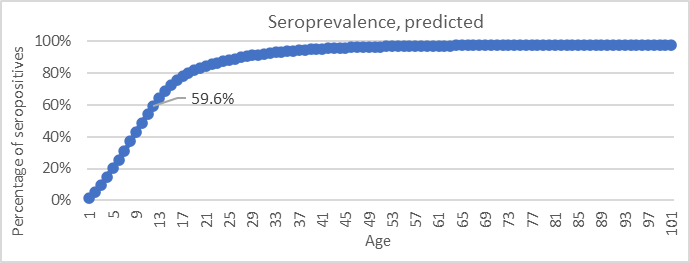


##### Fig B. Panel A shows incidence rate of symptomatic dengue, by age. Panel B shows incidence rate of hospitalized dengue, by age.


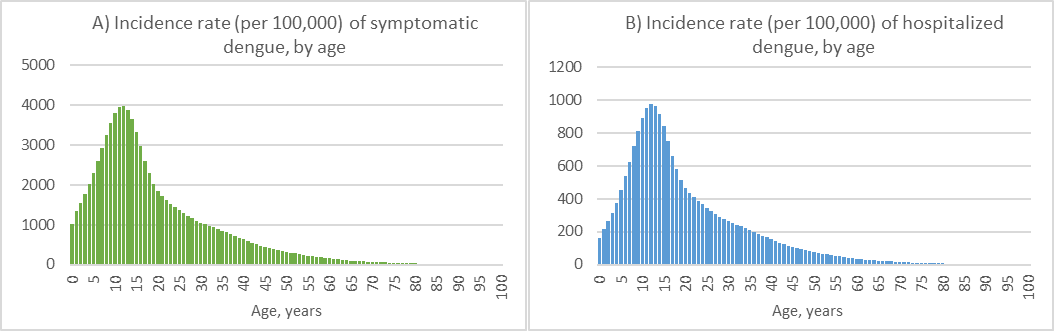


##### Fig C. Incidence rate of symptomatic dengue (before adjustment to underreporting), by calendar month.


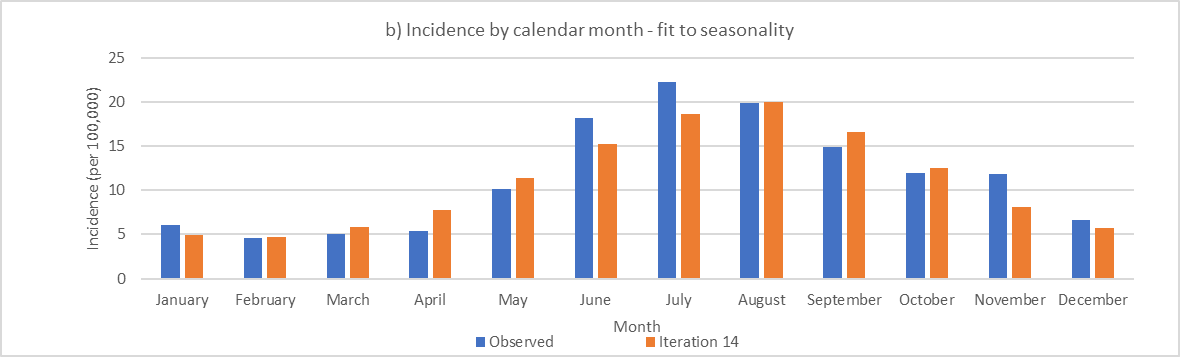


# Cohort optimization

This section presents the results of the 24 vaccination strategies (3 sizes of vaccination cohorts each with 8 routine ages) simulated as part of the cohort optimization analysis. The results are for the number of infections and hospitalizations are presented over 20 years (Table 1) and 10 years (Table 2). The costs and disability-adjusted life-years associated with each strategy are reported in Table 3 (20 years) and Table 4 (10 years). The strategy with the highest impact in a specific category is highlighted in bold. Threshold analysis for the unit cost of TAK-003 is reported in Table 5.

###### Table 1. Proportion of infections avoided for vaccination strategy versus no vaccination (over 20 years).

| **Strategy** | **Total infections** | **Asymptomatic** | **Symptomatic, total** | **Symptomatic, hospitalized** | **Symptomatic, nonhospitalized** | **Dengue-caused deaths** |
| --- | --- | --- | --- | --- | --- | --- |
| **No catch-up, routine vaccination at age** | | | | | | |
| 4 years | 34.53% | 31.18% | 40.67% | 47.47% | 38.57% | 47.47% |
| 5 years | 35.30% | 31.79% | 41.74% | 48.95% | 39.53% | 48.95% |
| 6 years | 35.81% | 32.15% | 42.53% | 50.09% | 40.20% | 50.09% |
| 7 years | **36.03%** | **32.24%** | 42.97% | 50.85% | **40.55%** | 50.85% |
| 8 years | 35.92% | 32.04% | **43.04%** | **51.15%** | 40.54% | **51.15%** |
| 9 years | 35.47% | 31.54% | 42.67% | 50.93% | 40.13% | 50.93% |
| 10 years | 34.65% | 30.74% | 41.84% | 50.14% | 39.28% | 50.14% |
| 11 years | 33.46% | 29.61% | 40.52% | 48.75% | 37.99% | 48.75% |
| **Catch-up of 5 cohorts, routine vaccination at age** | | | | | | |
| 4 years | 41.11% | 36.24% | 50.04% | 60.63% | 46.79% | 60.63% |
| 5 years | **41.34%** | **36.33%** | 50.52% | 61.47% | 47.15% | 61.47% |
| 6 years | 41.31% | 36.20% | **50.68%** | 61.94% | **47.22%** | 61.94% |
| 7 years | 41.03% | 35.85% | 50.53% | **62.00%** | 47.00% | **62.00%** |
| 8 years | 40.50% | 35.30% | 50.04% | 61.63% | 46.47% | 61.63% |
| 9 years | 39.65% | 34.48% | 49.15% | 60.75% | 45.58% | 60.75% |
| 10 years | 38.45% | 33.35% | 47.81% | 59.32% | 44.26% | 59.32% |
| 11 years | 36.84% | 31.87% | 45.96% | 57.28% | 42.47% | 57.28% |
| **Catch-up of 10 cohorts, routine vaccination at age** | | | | | | |
| 4 years | 46.14% | 40.45% | 56.59% | 69.67% | 52.57% | 69.67% |
| 5 years | **46.42%** | **40.72%** | **56.87%** | 70.01% | **52.83%** | 70.01% |
| 6 years | 46.40% | 40.71% | 56.85% | **70.02%** | 52.79% | **70.02%** |
| 7 years | 46.04% | 40.37% | 56.46% | 69.65% | 52.40% | 69.65% |
| 8 years | 45.31% | 39.66% | 55.68% | 68.86% | 51.62% | 68.86% |
| 9 years | 44.21% | 38.61% | 54.49% | 67.63% | 50.45% | 67.63% |
| 10 years | 42.78% | 37.27% | 52.91% | 65.93% | 48.90% | 65.93% |
| 11 years | 41.02% | 35.63% | 50.92% | 63.73% | 46.97% | 63.73% |
| Data presented are at the population level of Thailand over a 20-year time horizon with no discounting. Catch-up vaccination is administered in the first years of vaccine introduction. For example, the row “4 years” in the cluster “Catch-up of 5 cohorts, routine vaccination at age” represents the results of the strategy with annual routine vaccination of children aged 4 years and catch-up vaccination of children aged 5–9 years.  The strategy with the highest impact in a specific category is highlighted in bold. | | | | | | |

###### Table 2. Proportion of infections avoided for vaccination strategy versus no vaccination (over 10 years).

| **Strategy** | **Total infections** | **Asymptomatic** | **Symptomatic, total** | **Symptomatic, hospitalized** | **Symptomatic, nonhospitalized** | **Dengue-caused deaths** |
| --- | --- | --- | --- | --- | --- | --- |
| **No catch-up, routine vaccination at age** | | | | | | |
| 4 years | 27.92% | 26.08% | 31.30% | 34.21% | 30.40% | 34.21% |
| 5 years | 30.42% | 28.43% | 34.06% | 37.25% | 33.08% | 37.25% |
| 6 years | 32.78% | 30.66% | 36.66% | 40.10% | 35.60% | 40.10% |
| 7 years | 34.84% | 32.62% | 38.92% | 42.57% | 37.79% | 42.57% |
| 8 years | 36.45% | 34.14% | 40.68% | 44.51% | 39.51% | 44.51% |
| 9 years | 37.43% | 35.06% | 41.78% | 45.75% | 40.56% | 45.75% |
| 10 years | **37.63%** | **35.22%** | **42.05%** | **46.12%** | **40.79%** | **46.12%** |
| 11 years | 36.97% | 34.55% | 41.40% | 45.52% | 40.13% | 45.52% |
| **Catch-up of 5 cohorts, routine vaccination at age** | | | | | | |
| 4 years | 51.47% | 48.52% | 56.89% | 62.14% | 55.28% | 62.14% |
| 5 years | 53.80% | 50.78% | 59.35% | 64.77% | 57.68% | 64.77% |
| 6 years | 55.60% | 52.52% | 61.24% | 66.82% | 59.52% | 66.82% |
| 7 years | 56.77% | 53.65% | 62.49% | 68.20% | 60.73% | 68.20% |
| 8 years | **57.26%** | **54.10%** | **63.04%** | **68.85%** | **61.25%** | **68.85%** |
| 9 years | 57.00% | 53.82% | 62.82% | 68.72% | 61.00% | 68.72% |
| 10 years | 55.96% | 52.77% | 61.80% | 67.77% | 59.96% | 67.77% |
| 11 years | 54.12% | 50.94% | 59.95% | 65.94% | 58.11% | 65.94% |
| **Catch-up of 10 cohorts, routine vaccination at age** | | | | | | |
| 4 years | 69.05% | 66.11% | 74.43% | 80.02% | 72.71% | 80.02% |
| 5 years | 69.95% | 67.03% | 75.30% | 80.90% | 73.58% | 80.90% |
| 6 years | 70.44% | 67.53% | 75.77% | 81.38% | 74.04% | 81.38% |
| 7 years | **70.48%** | **67.57%** | **75.83%** | **81.47%** | **74.09%** | **81.47%** |
| 8 years | 70.04% | 67.10% | 75.42% | 81.13% | 73.66% | 81.13% |
| 9 years | 69.03% | 66.05% | 74.49% | 80.30% | 72.70% | 80.30% |
| 10 years | 67.39% | 64.35% | 72.94% | 78.89% | 71.11% | 78.89% |
| 11 years | 65.07% | 61.98% | 70.73% | 76.83% | 68.86% | 76.83% |
| Data presented are at the population level of Thailand over a 10-year time horizon with no discounting. Catch-up vaccination is administered in the first years of vaccine introduction. For example, the row “4 years” in the cluster “Catch-up of 5 cohorts, routine vaccination at age” represents the results of the strategy with annual routine vaccination of children aged 4 years and catch-up vaccination of children aged 5–9 years.  The strategy with the highest impact in a specific category is highlighted in bold. | | | | | | |

###### Table 3. Incremental costs and effectiveness for vaccination strategy versus no vaccine at 20-year timeframe (discount rate 3%). Costs are reported in million US dollars.

| **Strategy** | **Direct medical cost** | **Direct nonmedical cost** | **Productivity loss** | **Cost of school absenteeism** | **Vaccine and administration** | **Total cost** | **Total DALYs** |
| --- | --- | --- | --- | --- | --- | --- | --- |
| **No catch-up, routine vaccination at age** | | | | | | | |
| 4 years | –1,214 | –151 | –435 | –27 | 685 | –1,143 | –66,106 |
| 5 years | –1,260 | –156 | –452 | –29 | 684 | –1,212 | –68,471 |
| 6 years | –1,297 | –161 | –465 | –30 | 684 | –1,269 | –70,366 |
| 7 years | –1,324 | –164 | –474 | **–31** | 684 | –1,309 | –71,689 |
| 8 years | **–1,339** | **–165** | **–479** | –31 | 684 | **–1,330** | **–72,324** |
| 9 years | –1,338 | –165 | –479 | –30 | 683 | –1,330 | –72,165 |
| 10 years | –1,322 | –163 | –473 | –29 | 683 | –1,304 | –71,122 |
| 11 years | –1,287 | –159 | –461 | –28 | 683 | –1,252 | –69,153 |
| **Catch-up of 5 cohorts, routine vaccination at age** | | | | | | | |
| 4 years | –1,637 | –202 | –581 | –36 | 908 | –1,549 | –88,057 |
| 5 years | –1,668 | –205 | –592 | –37 | 907 | –1,596 | –89,566 |
| 6 years | –1,688 | –208 | –599 | **–38** | 907 | –1,627 | –90,499 |
| 7 years | **–1,696** | **–209** | **–602** | –38 | 907 | **–1,638** | **–90,814** |
| 8 years | –1,691 | –208 | –600 | –37 | 907 | –1,630 | –90,444 |
| 9 years | –1,670 | –205 | –593 | –36 | 906 | –1,599 | –89,298 |
| 10 years | –1,633 | –200 | –580 | –35 | 906 | –1,542 | –87,283 |
| 11 years | –1,578 | –193 | –561 | –33 | 905 | –1,459 | –84,333 |
| **Catch-up of 10 cohorts, routine vaccination at age** | | | | | | | |
| 4 years | –1,927 | –237 | –685 | –41 | 1,130 | –1,760 | –103,420 |
| 5 years | –1,940 | –239 | –690 | –42 | 1,130 | –1,780 | –104,176 |
| 6 years | **–1,942** | **–239** | **–692** | **–42** | 1,130 | **–1,786** | **–104,415** |
| 7 years | –1,934 | –238 | –689 | –41 | 1,129 | –1,774 | –104,067 |
| 8 years | –1,914 | –235 | –683 | –40 | 1,129 | –1,744 | –103,069 |
| 9 years | –1,881 | –231 | –671 | –39 | 1,128 | –1,694 | –101,381 |
| 10 years | –1,835 | –225 | –654 | –37 | 1,128 | –1,624 | –98,976 |
| 11 years | –1,775 | –218 | –633 | –35 | 1,127 | –1,533 | –95,816 |
| Data presented are at the population level of Thailand over a 20-year time horizon with 3% discount rate.  The total cost includes all the subcategories displayed in the table and reflects a societal perspective. The incremental costs are calculated versus no vaccination; a negative incremental cost indicates that the vaccination strategy is less costly and a positive incremental cost indicates that the vaccination strategy is more costly. The incremental DALYs are calculated versus no vaccination; a negative number indicates that the vaccination strategy is more effective than no vaccination and a positive number indicates that the vaccination strategy is less effective than no vaccination.  The strategy with the highest impact in a specific category is highlighted in bold.  DALY, disability-adjusted life-year. | | | | | | | |

###### Table 4. Incremental costs and effectiveness for vaccination strategies versus no vaccine at 10-year timeframe (discount rate 3%). Costs are reported in million US dollars.

| **Strategy** | **Direct medical cost** | **Direct nonmedical cost** | **Productivity loss** | **Cost of school absenteeism** | **Vaccine and administration** | **Total cost** | **Total DALYs** |
| --- | --- | --- | --- | --- | --- | --- | --- |
| **No catch-up, routine vaccination at age** | | | | | | | |
| 4 years | –527 | –66 | –194 | –11 | 393 | –406 | –29,218 |
| 5 years | –574 | –72 | –212 | –13 | 392 | –479 | –31,872 |
| 6 years | –620 | –78 | –229 | –15 | 392 | –549 | –34,373 |
| 7 years | –659 | –83 | –244 | –15 | 392 | –609 | –36,576 |
| 8 years | –691 | –87 | –256 | –16 | 392 | –657 | –38,321 |
| 9 years | –711 | –89 | –263 | **–16** | 392 | –689 | –39,443 |
| 10 years | **–718** | **–90** | **–266** | –16 | 392 | **–699** | **–39,796** |
| 11 years | –710 | –89 | –263 | –16 | **392** | –687 | –39,279 |
| **Catch-up of 5 cohorts, routine vaccination at age** | | | | | | | |
| 4 years | –989 | –125 | –366 | –22 | 616 | –886 | –54,998 |
| 5 years | –1,034 | –130 | –383 | –23 | 615 | –955 | –57,487 |
| 6 years | –1,069 | –135 | –397 | –24 | 615 | –1,009 | –59,432 |
| 7 years | –1,092 | –138 | –406 | **–24** | 615 | –1,046 | –60,750 |
| 8 years | **–1,104** | **–139** | **–411** | –24 | 615 | **–1,064** | **–61,372** |
| 9 years | –1,103 | –139 | –411 | –24 | 615 | –1,061 | –61,242 |
| 10 years | –1,087 | –137 | –405 | –23 | 614 | –1,038 | –60,313 |
| 11 years | –1,057 | –133 | –394 | –22 | **614** | –992 | –58,565 |
| **Catch-up of 10 cohorts, routine vaccination at age** | | | | | | | |
| 4 years | –1,295 | –164 | –486 | –27 | 838 | –1,134 | –72,445 |
| 5 years | –1,310 | –166 | –492 | –28 | 838 | –1,158 | –73,298 |
| 6 years | –1,318 | –167 | –496 | **–28** | 838 | –1,171 | –73,757 |
| 7 years | **–1,320** | **–167** | **–497** | –28 | 837 | **–1,174** | **–73,814** |
| 8 years | –1,314 | –166 | –495 | –27 | 837 | –1,165 | –73,435 |
| 9 years | –1,299 | –164 | –489 | –27 | 837 | –1,143 | –72,559 |
| 10 years | –1,275 | –161 | –480 | –26 | 836 | –1,106 | –71,116 |
| 11 years | –1,240 | –156 | –467 | –25 | **836** | –1,053 | –69,076 |
| Data presented are at the population level of Thailand over a 10-year time horizon with 3% discount rate.  The total cost includes all the subcategories displayed in the table and reflects a societal perspective. The incremental costs are calculated versus no vaccination; a negative incremental cost indicates that the vaccination strategy is less costly and a positive incremental cost indicates that the vaccination strategy is more costly. The incremental DALYs are calculated versus no vaccination; a negative number indicates that the vaccination strategy is more effective than no vaccination and a positive number indicates that the vaccination strategy is less effective than no vaccination.  The strategy with the highest impact in a specific category is highlighted in bold.  DALY, disability-adjusted life-year. | | | | | | | |

###### Table 5. Cost-effective threshold pricing analysis at discount rate of 3% over 20 years for dominance or with WTP threshold of US $7,000 (1 × GDP)/DALY avoided.

| **Strategy** | **Threshold price (per dose)** | |
| --- | --- | --- |
|  | **Vaccination with TAK-003 is dominant compared with no vaccination (more effective and cost saving)** | **Vaccination with TAK-003 is cost-effective under the WTP threshold of $7,000 per DALY averted** |
| **No catch-up, routine vaccination at age** | | |
| 4 years | 83.93 | 105.76 |
| 5 years | 87.21 | 109.83 |
| 6 years | 89.91 | 113.17 |
| 7 years | 91.83 | 115.53 |
| 8 years | **92.85** | **116.77** |
| 9 years | 92.84 | 116.71 |
| 10 years | 91.66 | 115.19 |
| 11 years | 89.22 | 112.12 |
| **Catch-up of 5 cohorts, routine vaccination at age** | | |
| 4 years | 85.11 | 107.04 |
| 5 years | 86.81 | 109.12 |
| 6 years | 87.92 | 110.48 |
| 7 years | **88.36** | **111.00** |
| 8 years | 88.07 | 110.63 |
| 9 years | 86.98 | 109.27 |
| 10 years | 85.00 | 106.78 |
| 11 years | 82.06 | 103.12 |
| **Catch-up of 10 cohorts, routine vaccination at age** | | |
| 4 years | 80.30 | 100.99 |
| 5 years | 80.89 | 101.73 |
| 6 years | **81.06** | **101.96** |
| 7 years | 80.74 | 101.58 |
| 8 years | 79.90 | 100.55 |
| 9 years | 78.51 | 98.82 |
| 10 years | 76.53 | 96.38 |
| 11 years | 73.95 | 93.18 |
| Data presented are at the population level of Thailand over a 20-year time horizon with 3% discount rate.  The strategy with the highest impact in a specific category is highlighted in bold.  DALY, disability-adjusted life-year; GDP, gross domestic product; WTP, willingness-to-pay. | | |

# Pragmatic scenario

## Impact of coverage rates

The impact of coverage rate on the number of cases of dengue and hospitalizations averted is reported in Table 6.

###### Table 6. Proportion of cases avoided with R11 over 20 years, by coverage rate.

| **Coverage rate** | **Symptomatic cases** | **Hospitalized cases** |
| --- | --- | --- |
| 0% | 0% | 0% |
| 10% | 4% | 6% |
| 20% | 9% | 12% |
| 30% | 14% | 18% |
| 40% | 19% | 24% |
| 50% | 25% | 31% |
| 60% | 30% | 37% |
| 70% | 35% | 43% |
| 80% | 41% | 49% |
| 87% | 44% | 53% |
| 90% | 45% | 54% |
| R11 strategy is routine vaccination at age 11 years without catch-up. Data presented are at the population level of Thailand over a 20-year time horizon with no discounting. | | |

## Scenario analyses

### Description of scenarios

A list of the scenarios, with an explanation of how they differ from the base case, is reported in Table 7.

###### Table 7. List of scenarios tested for the comparison of routine vaccination versus no vaccination.

| Number | Scenario name | Scenario description | Base case description |
| --- | --- | --- | --- |
| Scenarios around boosting of efficacy and efficacy against asymptomatic infection | | | |
| 1 | Boosting of efficacy against asymptomatic infection | Boosting of efficacy against asymptomatic dengue included | Boosting of efficacy against asymptomatic dengue not included |
| 2 | No boosting of effect | Natural boosting of efficacy against symptomatic dengue not included | Natural boosting of efficacy against symptomatic dengue included |
| 3 | No efficacy against asymptomatic infection | No efficacy against asymptomatic dengue | Efficacy against asymptomatic dengue equal to half of efficacy against nonhospitalized dengue |
| 4 | No efficacy against asymptomatic infection and no boosting of efficacy | Natural boosting of efficacy against symptomatic dengue not included  No efficacy for asymptomatic dengue | Natural boosting of efficacy against symptomatic dengue included  Efficacy for asymptomatic dengue equal to half of efficacy against nonhospitalized dengue |
| Scenarios around efficacy against nonhospitalized infection | | | |
| 5 | Efficacy against nonhospitalized, low case | Efficacy against nonhospitalized dengue reduced to lower confidence interval (S2 Material, Section 2.4) | Base case efficacy against nonhospitalized dengue (S2 Material, Section 2.4) |
| 6 | Efficacy against nonhospitalized, high case | Efficacy against nonhospitalized dengue increased to upper confidence interval (S2 Material, Section 2.4) | Base case efficacy against nonhospitalized dengue (S2 Material, Section 2.4) |
| **Serostatus and serotype-specific efficacy** | | | |
| 7 | Serostatus- and serotype-specific efficacy | Efficacy against nonhospitalized and hospitalized dengue differentiated by serotype (in addition to differentiation by serostatus; S2 Material, Section 2.4) | Base case efficacy against nonhospitalized and hospitalized dengue (S2 Material, Section 2.4) |
| **Scenarios around efficacy against hospitalized infection** | | | |
| 8 | Efficacy against hospitalized, low case | Efficacy against hospitalized dengue reduced to lower confidence interval (S2 Material, Section 2.4) | Base case efficacy against hospitalized dengue (S2 Material, Section 2.4) |
| 9 | Efficacy against hospitalized, high case | Efficacy against hospitalized dengue increased to upper confidence interval (S2 Material, Section 2.4) | Base case efficacy against hospitalized dengue (S2 Material, Section 2.4) |
| 10 | Efficacy against hospitalized, waning | Efficacy against hospitalized dengue wanes over time (S2 Material, Section 2.4) | Efficacy against hospitalized dengue is constant over time |
| Scenarios around indirect effect | | | |
| 11 | Same transmissibility of symptomatic and asymptomatic infection | Symptomatic infections are as transmissible as asymptomatic infections | Symptomatic infections are twice as transmissible as asymptomatic infections |
| 12 | Same transmissibility of symptomatic and asymptomatic infection, no efficacy against asymptomatic infection | Symptomatic infections are as transmissible as asymptomatic infections  No efficacy for asymptomatic dengue | Symptomatic infections are twice as transmissible as asymptomatic infections  Efficacy for asymptomatic dengue equal to half of efficacy against nonhospitalized dengue |
| **Scenario around costs perspective** | | | |
| 13 | Payer perspective | Payer perspective | Societal perspective |

### Public health impact

The absolute results of scenario analyses in terms of impact on public health (cases of dengue and hospitalizations) are reported in Table 8. The incremental results are reported in Table 9.

###### Table 8. Number of infections over 20 years scenario analysis for R11.

| **Scenario^a^** | **Total infections** | **Asymptomatic** | **Symptomatic, total** | **Symptomatic, hospitalized** | **Symptomatic, nonhospitalized** | **Dengue-caused deaths** |
| --- | --- | --- | --- | --- | --- | --- |
| No vaccination (base case) | 41,273,513 | 26,718,297 | 14,555,216 | 3,425,852 | 11,129,364 | 3,500 |
| No vaccination^b^ | 42,496,088 | 27,625,327 | 14,870,761 | 3,497,971 | 11,372,791 | 3,574 |
| Base case: R11 | 26,179,217 | 18,020,095 | 8,159,122 | 1,626,637 | 6,532,485 | 1,662 |
| **Scenarios around boosting of efficacy and efficacy against asymptomatic infection** | | | | | | |
| 1 | 25,351,055 | 17,411,513 | 7,939,542 | 1,582,275 | 6,357,267 | 1,617 |
| 2 | 26,930,167 | 18,452,441 | 8,477,725 | 1,671,662 | 6,806,064 | 1,708 |
| 3 | 32,808,174 | 23,019,390 | 9,788,784 | 1,989,908 | 7,798,876 | 2,033 |
| 4 | 33,637,304 | 23,472,653 | 10,164,651 | 2,039,421 | 8,125,230 | 2,084 |
| **Scenarios around efficacy against nonhospitalized infection** | | | | | | |
| 5 | 28,958,890 | 19,804,460 | 9,154,430 | 1,791,886 | 7,362,544 | 1,831 |
| 6 | 22,194,914 | 15,398,874 | 6,796,040 | 1,390,492 | 5,405,548 | 1,421 |
| **Serostatus and serotype-specific efficacy** | | | | | | |
| 7 | 27,640,428 | 18,841,089 | 8,799,339 | 1,822,421 | 6,976,918 | 1,862 |
| **Scenarios around efficacy against hospitalized infection** | | | | | | |
| 8 | 26,537,090 | 18,222,017 | 8,315,073 | 1,697,311 | 6,617,763 | 1,734 |
| 9 | 25,923,527 | 17,874,883 | 8,048,645 | 1,577,204 | 6,471,440 | 1,611 |
| 10 | 27,271,102 | 18,653,042 | 8,618,060 | 1,823,798 | 6,794,262 | 1,863 |
| **Scenarios around indirect effect** | | | | | | |
| 11 | 34,952,393 | 24,214,215 | 10,738,178 | 2,160,822 | 8,577,356 | 2,208 |
| 12 | 42,496,088 | 30,048,061 | 12,448,028 | 2,570,716 | 9,877,312 | 2,626 |
| R11 strategy is routine vaccination at age 11 years without catch-up. Data presented are at the population level of Thailand over a 20-year time horizon with no discounting.  ^a^Scenario numbers correspond to those listed in Table 7: List of scenarios tested for the comparison of routine vaccination versus no vaccination.  ^b^Assuming the same transmissibility for symptomatic and asymptomatic spread. | | | | | | |

###### Table 9. Number (percentage) of infections avoided (over 20 years, undiscounted): scenario analysis for R11.

| **Scenario^a^** | **Total infections** | **Asymptomatic** | **Symptomatic, total** | **Symptomatic, hospitalized** | **Symptomatic,  nonhospitalized** | **Dengue-caused deaths** |
| --- | --- | --- | --- | --- | --- | --- |
| Base case: R11 | 15,094,296 (37%) | 8,698,202 (33%) | 6,396,094 (44%) | 1,799,215 (53%) | 4,596,879 (41%) | 1,838 (53%) |
| **Scenarios around boosting of efficacy and efficacy against asymptomatic infection** | | | | | | |
| 1 | 15,922,458 (39%) | 9,306,784 (35%) | 6,615,674 (45%) | 1,843,577 (54%) | 4,772,097 (43%) | 1,884 (54%) |
| 2 | 14,343,346 (35%) | 8,265,855 (31%) | 6,077,491 (42%) | 1,754,191 (51%) | 4,323,300 (39%) | 1,792 (51%) |
| 3 | 8,465,339 (21%) | 3,698,907 (14%) | 4,766,432 (33%) | 1,435,944 (42%) | 3,330,488 (30%) | 1,467 (42%) |
| 4 | 7,636,209 (19%) | 3,245,644 (12%) | 4,390,565 (30%) | 1,386,431 (40%) | 3,004,134 (27%) | 1,416 (40%) |
| **Scenarios around efficacy against nonhospitalized infection** | | | | | | |
| 5 | 12,314,623 (30%) | 6,913,836 (26%) | 5,400,786 (37%) | 1,633,966 (48%) | 3,766,820 (34%) | 1,669 48%) |
| 6 | 19,078,599 (46%) | 11,319,423 (42%) | 7,759,176 (53%) | 2,035,360 (59%) | 5,723,816 (51%) | 2,079 (59%) |
| **Serostatus and serotype-specific efficacy** | | | | | | |
| 7 | 13,633,085  (33%) | 7,877,208  (29%) | 5,755,877  (40%) | 1,603,432  (47%) | 4,152,445  (37%) | 1,638 (47%) |
| **Scenarios around efficacy against hospitalized infection** | | | | | | |
| 8 | 14,736,423 (36%) | 8,496,280 (32%) | 6,240,143 (43%) | 1,728,542 (50%) | 4,511,601 (41%) | 1,766 (50%) |
| 9 | 15,349,985 (37%) | 8,843,414 (33%) | 6,506,571 (45%) | 1,848,648 (54%) | 4,657,923 (42%) | 1,889 (54%) |
| 10 | 14,002,411 (34%) | 8,065,255 (30%) | 5,937,156 (41%) | 1,602,054 (47%) | 4,335,102 (39%) | 1,637 (47%) |
| **Scenarios around indirect effect** | | | | | | |
| 11 | 7,543,695 (18%) | 3,411,112 (12%) | 4,132,583 (28%) | 1,337,148 (38%) | 2,795,435 (25%) | 1,366 (38%) |
| 12 | 0  (0%) | –2,422,734  (–9%) | 2,422,734 (16%) | 927,255  (27%) | 1,495,479 (13%) | 947  (27%) |
| R11 strategy is routine vaccination at age 11 years without catch-up. Data presented are at the population level of Thailand over a 20-year time horizon with no discounting.  ^a^Scenario numbers correspond to those listed in Table 7. | | | | | | |

### Cost-effectiveness

The cost-effectiveness of each strategy in the scenario analysis is reported in Table 10. The threshold analysis for the cost of 1 dose of TAK-003 for each scenario is reported in Table 11.

###### Table 10. Cost-saving and effectiveness for routine vaccination strategy versus no vaccine at 20-year timeframe (discount rate 3%): scenario analyses. Costs are reported in million US dollars.

| **Scenario^a^** | **Direct medical cost** | **Direct nonmedical cost** | **Productivity loss** | **Cost of school absence** | **Cost of vaccine and admin** | **Total cost savings** | **Total DALYs** |
| --- | --- | --- | --- | --- | --- | --- | --- |
| Base case: R11 | 1,388 | 171 | 499 | 30 | –743 | 1,346 | 74,744 |
| **Scenarios around boosting of efficacy and efficacy against asymptomatic infection** | | | | | | | |
| 1 | 1,423 | 176 | 513 | 31 | –743 | 1,400 | 76,887 |
| 2 | 1,348 | 166 | 479 | 29 | –743 | 1,280 | 72,366 |
| 3 | 1,075 | 131 | 375 | 24 | –743 | 863 | 56,065 |
| 4 | 1,030 | 124 | 352 | 23 | –743 | 786 | 53,378 |
| **Scenarios around efficacy against nonhospitalized infection** | | | | | | | |
| 5 | 1,237 | 150 | 431 | 26 | –743 | 1,102 | 65,816 |
| 6 | 1,598 | 201 | 592 | 35 | –743 | 1,682 | 87,287 |
| **Serostatus and serotype-specific efficacy** | | | | | | | |
| 7 | 1,246 | 154 | 450 | 27 | –743 | 1,135 | 67,194 |
| **Scenarios around efficacy against hospitalized infection** | | | | | | | |
| 8 | 1,341 | 166 | 485 | 29 | –743 | 1,278 | 72,292 |
| 9 | 1,421 | 175 | 509 | 31 | –743 | 1,393 | 76,465 |
| 10 | 1,265 | 158 | 461 | 29 | –743 | 1,170 | 68,331 |
| **Scenarios around indirect effect** | | | | | | | |
| 11 | 994 | 119 | 337 | 22 | –743 | 730 | 50,565 |
| 12 | 630 | 72 | 199 | 15 | –743 | 174 | 29,167 |
| **Scenario around costs perspective** | | | | | | | |
| 13 | 1,388 | 0 | 0 | 0 | –743 | 645 | 74,744 |
| R11 strategy is routine vaccination at age 11 years without catch-up. Data presented are at the population level of Thailand over a 20-year time horizon with 3% discount rate.  The total cost includes all the subcategories displayed in the table and reflects societal perspective. The incremental costs are calculated versus no vaccination; a negative incremental cost indicates that the vaccination strategy is less costly. The incremental DALYs are calculated versus no vaccination; a negative number indicates that the vaccination strategy is more effective than no vaccination.  **^a^**Scenario numbers correspond to those listed in Table 7**:** List of scenarios tested for the comparison of routine vaccination versus no vaccination.  DALY, disability-adjusted life-year. | | | | | | | |

###### Table 11. Threshold price analysis at discount rate of 3% over 20 years for dominance or with WTP threshold of US $7,000 (1 × GDP)/DALY avoided.

| **Scenario^a^** | **Threshold price** | |
| --- | --- | --- |
|  | **Vaccination with TAK-003 is dominant compared to no vaccination (more effective and cost saving)** | **Vaccination with TAK-003 is cost-effective under the WTP threshold of $7,000 per DALY averted** |
| Base case: R11 | 88.53 | 111.29 |
| **Scenarios around boosting of efficacy and efficacy against asymptomatic infection** | | |
| 1 | 90.9 | 114.31 |
| 2 | 85.66 | 107.69 |
| 3 | 67.53 | 84.6 |
| 4 | 64.2 | 80.45 |
| **Scenarios around efficacy against nonhospitalized infection** | | |
| 5 | 77.93 | 97.96 |
| 6 | 103.18 | 129.75 |
| **Serostatus and serotype-specific efficacy** | | |
| 7 | 79.35 | 99.80 |
| **Scenarios around efficacy against hospitalized infection** | | |
| 8 | 85.61 | 107.61 |
| 9 | 90.58 | 113.86 |
| 10 | 80.91 | 101.71 |
| **Scenarios around indirect effect** | | |
| 11 | 61.77 | 77.16 |
| 12 | 37.55 | 46.43 |
| **Scenario around costs perspective** | | |
| 13 | 58.07 | 80.82 |
| R11 strategy is routine vaccination at age 11 years without catch-up. Data presented are at the population level of Thailand over a 20-year time horizon with 3% discount rate.  **^a^**Scenario numbers correspond to those listed in Table 7.  DALY, disability-adjusted life-year; GDP, gross domestic product; WTP, willingness-to-pay. | | |
